# Extraordinary kinetic inertness of lanthanide(iii) complexes of pyridine-rigidified 18-membered hexaazamacrocycles with four acetate pendant arms†

**DOI:** 10.1039/d5sc01893e

**Published:** 2025-05-30

**Authors:** Jan Faltejsek, Peter Urbanovský, Vojtěch Kubíček, Jana Havlíčková, Ivana Císařová, Jan Kotek, Petr Hermann

**Affiliations:** a Department of Inorganic Chemistry, Faculty of Science, Universita Karlova (Charles University) Hlavova 2030 12843 Prague 2 Czech Republic petrh@natur.cuni.cz +420-22195-1263

## Abstract

Large polyazamacrocycles are used for the complexation of large metal ions. However, their coordination chemistry has not been frequently studied until now. An eighteen-membered macrocycle with two rigidifying pyridine rings and four aliphatic amino groups substituted with four acetic acid pendants, H_4_pyta, provides a large ligand cavity and coordination number (CN) up to 10. Trivalent lanthanides were chosen to study the effect of metal ion size on the properties of H_4_pyta complexes. The complexes were formed under relatively mild conditions and two isomers were observed, depending on the Ln(iii) ion, in different mutual ratios during the synthesis. Going to smaller Ln(iii) ions, the CN decreases from 10 to 9. Stability constants of Ln(iii)–H_4_pyta complexes with CN 10 are comparable with those of Ln(iii)–H_4_dota complexes despite the lower overall basicity of H_4_pyta. In the ten-coordinated isomers, Ln(iii) ions are perfectly 3D-wrapped inside the ligand cavity, and the ligand is minimally distorted. It leads to extreme kinetic inertness of the complexes. Dissociation of the Ln(iii)–H_4_pyta complexes in 5 M HClO_4_ and at 90 °C is very slow and requires up to several hours; the inertness is 10^2^–10^4^ times higher than that of the Ln(iii)–H_4_dota complexes. The solid-state structures point to the symmetric wrapping of metal ions and CN 10 being responsible for the stability of species multiply protonated on the coordinated acetate groups. The results suggest that H_4_pyta can be considered a leading scaffold for the future development of ligands intended for large metal ion binding in nuclear medicine, *e.g.* for α-emitting radioisotopes from the bottom of the periodic table.

## Abstract 2

With Ln^3+^ ions, the H_4_pyta forms isomeric complexes having CN 10 or 9, and the ten-coordinated complexes are up to 10^4^ times more kinetically inert than the Ln(iii)–H_4_dota complexes.

## Graphical abstract

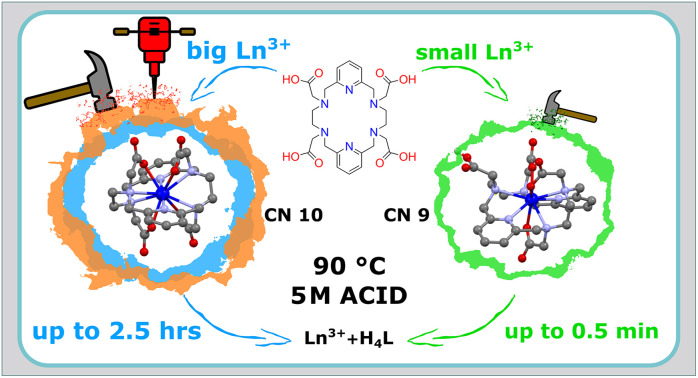

## Introduction

Over the last few years, several metal-based radiopharmaceuticals, *e.g.* Lutathera® or Pluvicto®, have started to be used as a standard treatment for some cancers.^1,2^ This has initiated the wide development of nuclear medicine employing the potential of radioisotopes of metallic elements across the periodic table. The metal radioisotopes are applied as cations and, therefore, must be bound by ligands/chelators into complexes which prevent non-specific deposition of the free radiometal ions in body tissues. To ensure their *in vivo* stability, the complexes must be thermodynamically stable and – more importantly – kinetically inert. In addition, the complexes used in radiopharmacy should be formed rapidly and under mild conditions due to (frequently) short half-lives of the metal radioisotopes and sensitivity of the used biological vectors (*e.g.* antibodies). Over time, the stability requirements have led to almost exclusive utilisation of pre-organized macrocyclic ligands with coordinating pendant arms for complexation of the metal radioisotopes.^3,4^ Macrocycles with different cavity sizes have been used according to the metal ion-specific demands. Derivatives of the small hexadentate H_3_nota are suitable for small octahedral metal ions such as Ga(iii). Analogues of the larger octadentate H_4_dota form stable and inert complexes with lanthanide(iii) ions. A strong equatorial ligand field of four nitrogen atoms in the cyclam derivatives leads to very stable and inert complexes of divalent copper. Among various ligand skeletons, the structure of H_4_dota is the most universal one, and many approved diagnostic and/or therapeutic probes use this particular ligand skeleton.

**Fig. 1:**
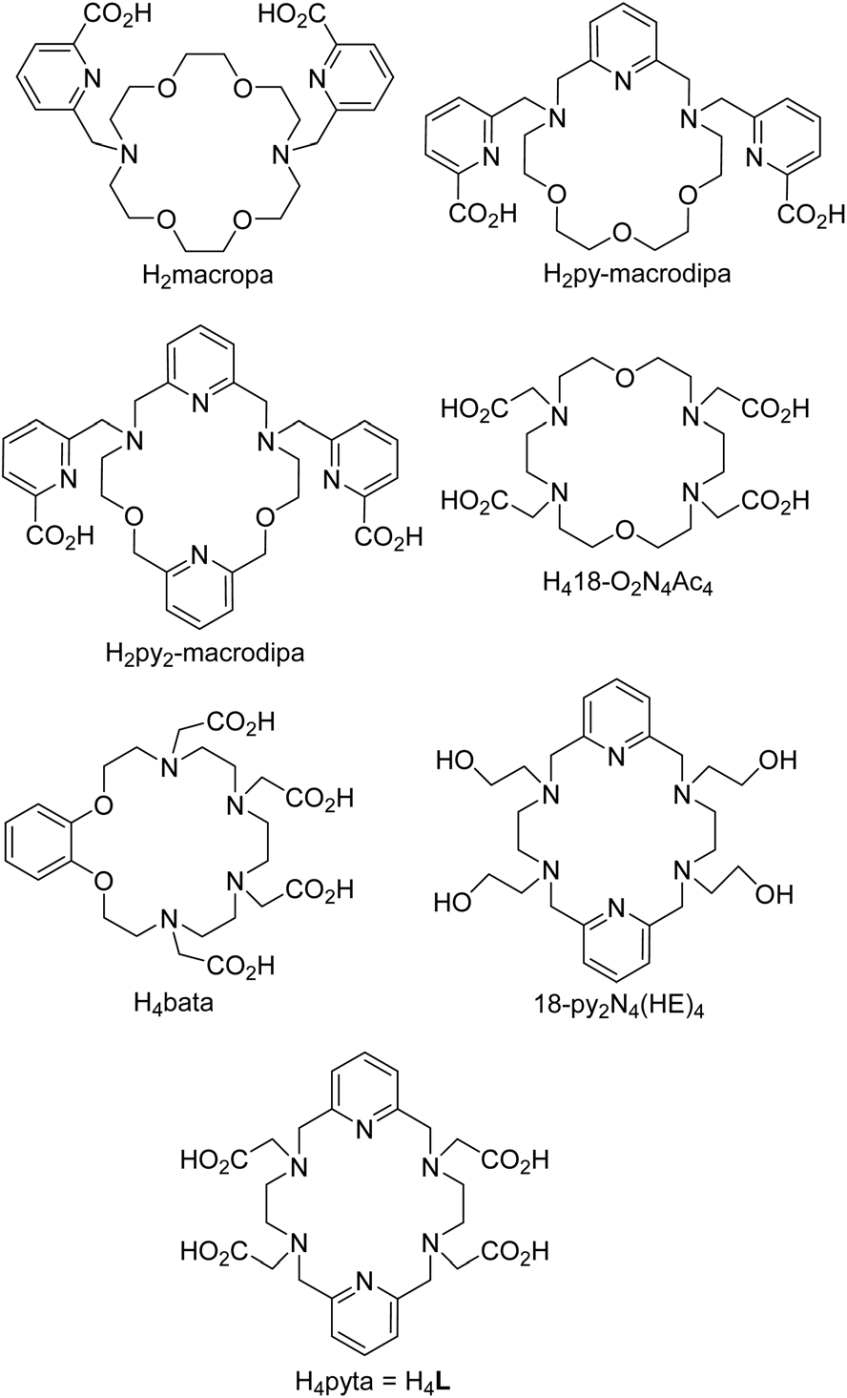
Structures of ligands mentioned in the text.

Recently, the interest in therapeutic radiopharmaceuticals has moved to α-particle-based therapy as this modality offers several advantages.^5^ The emitted α-particles deposit a huge amount of energy within only a short range in tissues (0.05–0.2 mm) which, if the tracer is bioaccumulated in cancer cells, minimises damage to the healthy tissues. Therefore, α-particle-based drugs are very much suitable for the treatment of small metastases. The α-emitting radioisotopes are mostly isotopes of elements from the bottom of the periodic table (*e.g.* isotopes of Pb, Bi, Ra or Ac). Common chelators are not usually able to bind their large ions effectively and with high *in vivo* stability of the formed complexes; anyway, H_4_dota derivatives are the most widespread chelators for these α-emitters. The large metal ions require a ligand cavity larger than that of H_4_dota and a coordination number (CN) higher than eight. Therefore, 18-membered macrocycles with six donor atoms and several coordinating pendant arms have been suggested and investigated. Among them, diazatetraoxo-18-crown modified with two picolinate pendants, H_2_macropa (Fig. 1), is shown to be highly promising.^6,7^ It exhibits thermodynamic selectivity for large Ln(iii) ions (the highest stability constant is observed for the Ce(iii) ion) as its ligand cavity is large and flexible and, together with the pendant arms, offers ten donor atoms (CN 10).^8^ It forms stable complexes even with Ac(iii).^6,9^ In a search for a ligand with improved properties, its pyridine-rigidified derivatives H_2_py-macrodipa and H_2_py_2_-macrodipa (Fig. 1) were prepared and their complexes studied.^10,11^ The selectivity for the large metal ions was preserved, but no significant improvement of properties of the complexes was observed. Other decadentate ligands investigated as carriers for the large metal radioisotopes have been 18-O_2_N_4_Ac_4_ (Fig. 1) or a similar benzene-rigidified ligand H_4_bata (Fig. 1).^12–14^

However, etheric oxygen atoms are not optimal donor atoms, and macrocycles containing more or only nitrogen donors should form more stable complexes. The ligand H_4_pyta (Fig. 1) having two pyridines, four aliphatic amino groups and four acetate oxygen donor atoms seems to be a more suitable chelator as it offers large 18-membered macrocycles, four charged oxygen donors and two pyridines rigidifying the whole structure. The chelator is not new; it was prepared in the mid-nineties, but it has been only sparingly investigated until now. One paper dealt with fluorescence properties of Eu(iii) and Tb(iii) complexes and with a relaxometric study of a Gd(iii) complex.^15^ In another work, stability constants of complexes of the ligand with some di/trivalent metal ions were obtained.^16^ A structural study in the solid state and solution showed coordination of all donor atoms in complexes of large Ln(iii) ions (*i.e.* forming complexes with CN 10). On the contrary, one pendant arm remains non-coordinated for small Ln(iii) ions, leading to CN 9.^17^

We decided to carry out more detailed investigations to determine whether the ligand is suitable as a possible carrier of metal radioisotopes. Surprisingly, we observed an extreme kinetic inertness of its Ln(iii) complexes, and the results are described in this work. During work on this project, two papers dealing with complexes of H_4_pyta appeared. The first one suggested H_4_pyta as a good chelator for several radioisotopes of trivalent metal ions and the other one contained a detailed study the of Pb(ii)–H_4_pyta complex.^18,19^

## Results and discussion

### Syntheses and isomerism of the complexes

The parent 18-membered polyazamacrocycle, 18-py_2_N_4_, and the title ligand, H_4_pyta, were prepared following modified published procedures, leading to higher yields (for detailed procedures, see the ESI†). Solid-state structures of the parent amine H_4_pyta and its tetrahydrochloride are discussed in the ESI.† To synthesize the Ln(iii)–H_4_pyta complexes, chosen conditions were the most common ones employed for the synthesis of Ln(iii)–H_4_dota and other complexes of macrocyclic ligands – ligand : LnCl_3_ in a molar ratio of 1 : 1.1, pH 6–7, and 60 °C (for the detailed procedure, see the ESI†). After full complexation (*i.e.* after full equilibration), mixtures of two isomers were usually detected by HPLC except for the La(iii)–Nd(iii) complexes where only one isomer was formed. The mutual ratio of the isomers depends on the Ln(iii) ion, consistent with the literature.^17^ These conditions are also close to the ones generally used for radiolabeling with lanthanide radioisotopes. Under different synthetic conditions (*e.g.* pH), the final equilibrated mixtures may have a different composition (see also below), mainly for ions in the middle of the lanthanide series where both isomers are accessible.

To analyse the geometry of the isomers, the isomeric Eu(iii) complexes were chosen as they show the highest thermodynamic stability and extreme kinetic inertness (see below). The molecular structures of both isomeric Eu(iii) complexes in the solid state were determined using single-crystal X-ray diffraction (for preparation of the single crystals, see the ESI†). One crystallographically independent complex molecule was found in the solid-state structure of the CN 10 isomer with the composition Na[Eu(pyta)]·13.5H_2_O. In contrast, the solid-state structure of the CN 9 complex in a phase with the composition [Eu(Hpyta)]·3H_2_O contains two independent molecules with very similar geometries. Selected structural parameters of the complexes are given in the ESI (Table S9†). The Eu–N coordination bond lengths in both isomers are similar (range: 2.58–2.68 Å and 2.57–2.70 Å for the CN 10 and CN 9 isomers, respectively), but the Eu–O distances are statistically significantly slightly shorter in the CN 9 isomer (2.33–2.35 Å) than in the CN 10 one (2.46–2.54 Å).

**Fig. 2:**
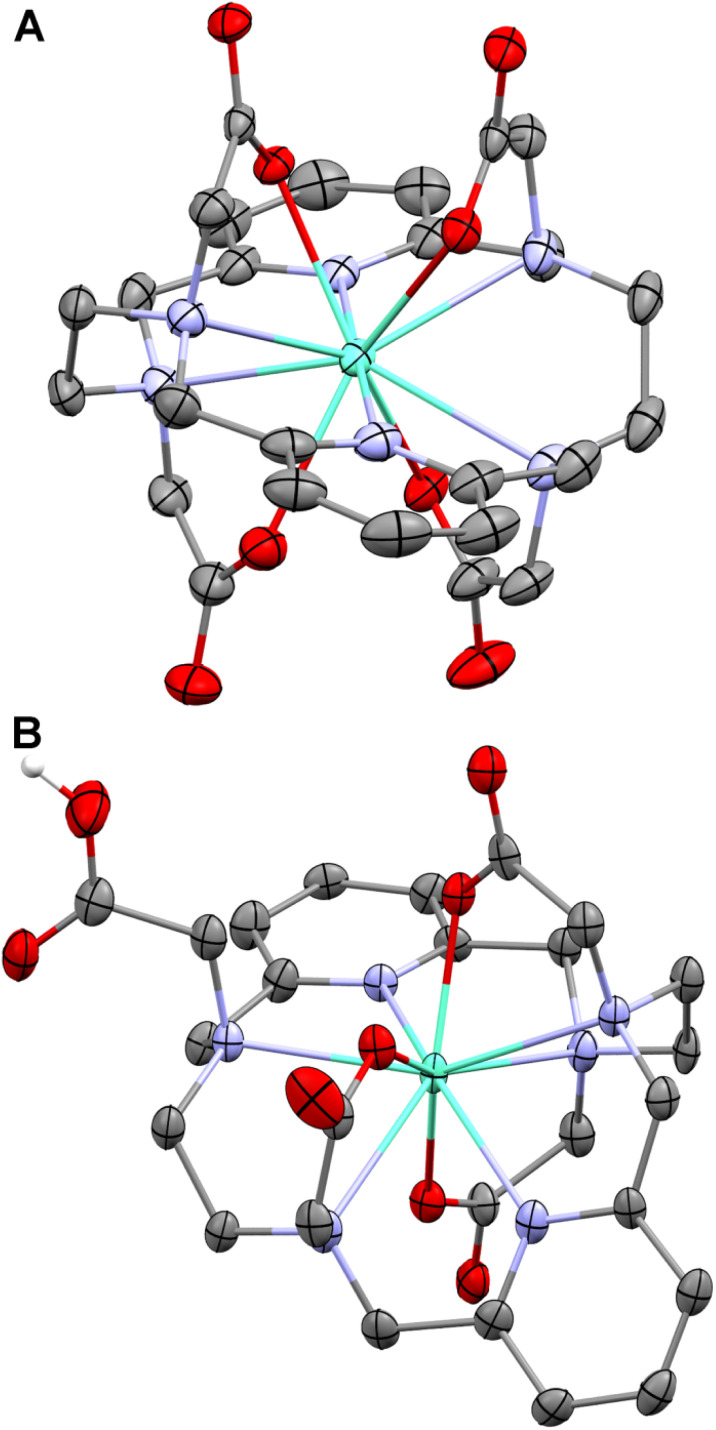
Molecular structures of the ^22^[Eu(pyta)]^−^ anion (CN 10) found in the crystal structure of Na^22^[Eu(pyta)]·13.5H_2_O (A) and one of the two structurally independent ^21^[Eu(Hpyta)] molecules (CN 9) present in the crystal structure of ^21^[Eu(Hpyta)]·3H2O (B). Superscripts 22 and 21 refer to complexes with CN 10 and 9, respectively (for more explanation, see the text). Carbon-bound hydrogen atoms are omitted for clarity. Colour codes: Eu: green, O: red, N: blue, C: dark grey, and H: white. A complete atom labelling scheme is given in the ESI (Fig. S8†).

The isomers significantly differ in the macrocycle conformation, which sterically determines the number of coordinated acetate pendant arms and their mutual orientation (Fig. 2). The isomer with CN 10 has roughly *D*_2_-symmetry (its symmetry corresponds to the shape of its NMR spectra; see the ESI† and ref. 17) with an almost linear arrangement of the N(pyridine1)–Eu–N(pyridine2) coordination bonds (angle, 179.3°), and the pyridine moieties are mutually only slightly tilted (angle, 21.4°). The nitrogen atoms do not form a fully regular plane due to large torsion angles along the aliphatic parts of the macrocycle. If assuming the mean N_6_-plane, the deviations of the aliphatic amino groups are symmetric (±∼0.9 Å), and the pyridine nitrogen atoms lie in the N_6_-plane. The central Eu(iii) ion lies almost perfectly in the centre of such this N_6_-plane. All acetate pendant arms are coordinated. The two arms bound to mutually “*trans*” ring amino groups are located on one side and the remaining two arms reside on the other side of the N_6_-plane.

The complex molecules of the CN 9 isomer are fully non-symmetric with formal *C*_1_ symmetry, consistent with the NMR spectra of the complexes (see the ESI† and ref. 17). The N(pyridine1)–Eu–N(pyridine2) coordination bonds are inclined with angles N1–Eu1–N10 = 148.0° and 147.2° for each independent molecule, respectively. Angles between planes of the pyridine rings of the individual macrocycles are 59.1° and 60.7°, respectively. The nitrogen atoms show large and irregular deviations from ideal N_6_-planes, and the central Eu(iii) ions lie 0.30 Å and 0.28 Å, respectively, away from such planes. Positions of the acetate pendants are very different if compared with the CN 10 isomer. Three pendant arms are directed to one side of the N_6_-plane, and two are coordinated and one is free (non-coordinated). The fourth acetate pendant arm (“*cis*” to the free one over the pyridine ring) is coordinated from the opposite side of the N_6_-plane. The complex species is monoprotonated, and the proton is attached to the non-coordinated acetate pendant arm.

In general, the macrocycle conformation of the CN 9 isomer is significantly more strained than the CN 10 one. It can be seen from a comparison of torsion angles along the macrocycle. For example, the torsion angle around the N(pyridine)–C(pyridine) bond should be ideally 180° due to the aromaticity of the pyridine fragment. The values found for the CN 10 isomer are in the range 174.3–177.0°, but for the CN 9 isomer, the values are more different from 180°, 168.3–178.7°. A difference is also evident in torsion angles of the N(pyridine)–C(pyridine)–CH_2_–N(aliphatic) fragments: for the CN 10 isomer, all torsion angles are similar and in a narrow range of 29.3–31.7°, whereas for the CN 9 isomer, the values are much more spread, 22.1–43.2°. Some differences are also observed between torsion angles of the ethylenediamine moieties, which should be 60° for an ideal staggered conformation. The values are slightly smaller (56.6–57.4°) for the CN 10 isomer, whereas they are somewhat bigger (61.6–65.1°) for the CN 9 isomer, compared to the ideal value.

For further discussion, we will label the isomers CN 10 and CN 9 as “22” and “21”, respectively, according to the location of the coordinated pendant arms. The label “22” means that 2 pendant arms are each coordinated to the metal ion on the opposite sides of the N_6_-plane, whereas the “21” isomer has 2 pendant arms bound on one side and 1 pendant arm bound on the other side of the N_6_-plane.

**Fig. 3:**
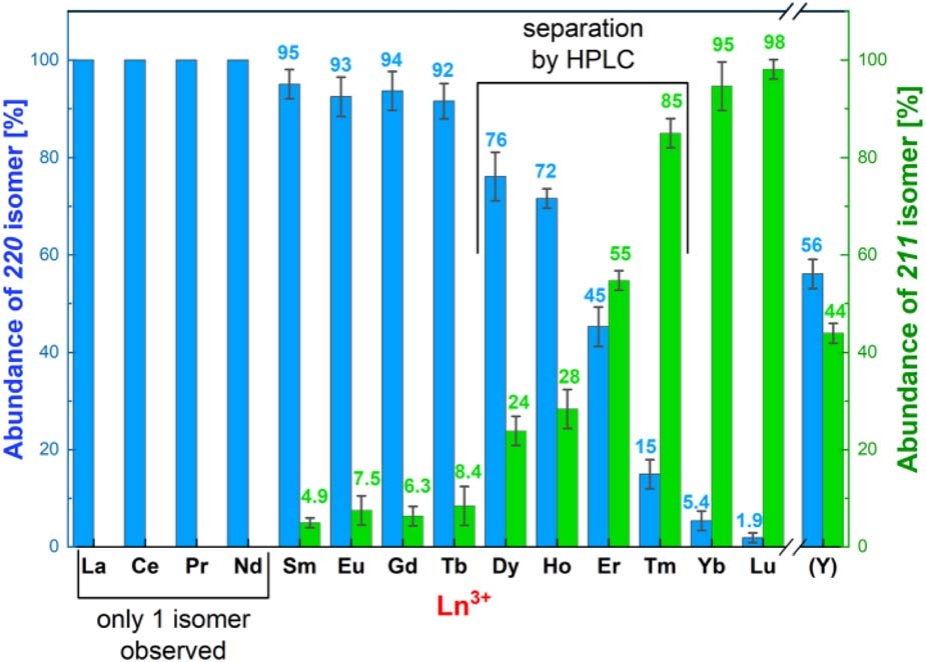
Compositions of the final reaction mixtures after synthesis (pH 6–7 and 60 °C) of the Ln(iii)–H_4_pyta complexes determined by analytical HPLC. The 22 isomers are in blue and the 21 isomers are in green. The error bars and the average abundances are based on three independent preparations.

Under used synthetic conditions, the 22 isomers were exclusively formed for the La(iii)–Nd(iii) complexes. An increasing abundance of the 21 isomers was observed for the other Ln(iii) ions and Y(iii) (Fig. 3). Except for the Dy(iii)–Er(iii) and Y(iii) complexes where abundances of both isomers were significant/comparable, precipitation of the complexes from the reaction mixtures led to isolation of solids (yields, 75–85%) containing pure 22 (La–Tb) or 21 (Yb and Lu) isomers, as proved by HPLC (Fig. S1 and S2 and Table S5†). Pure solid 22 and 21 isomers of the Dy(iii)–Tm(iii) and Y(iii) complexes were obtained after their separation by preparative HPLC followed by precipitation. The isolated complexes were characterized by MS (Table S5†) and ^1^H NMR (Table S6 and Fig. S3†), which confirmed the correct assignment of the 22/21 isomers based on their symmetry (*D*_2_*vs. C*_1_, respectively). The ^1^H NMR spectra of the paramagnetic complexes agreed with the published reports.^17^

To get information on the stability of the isomers in stock solutions used for kinetic/spectral measurements, mutual conversion of the isomers was semi-quantitatively followed for the Er(iii) complexes. At room temperature, the isomerization was very slow (days to weeks), and the process was faster in slightly acidic solutions (Fig. S4†). Thus, stock solutions can be used for the measurements, but their composition has to be regularly checked by HPLC.

### Equilibrium studies

Thermodynamic stability is one of the important parameters for evaluation of complexes considered for utilization in biology and medicine. First, protonation constants of H_4_pyta were determined and compared with related ligands (Table S10†). Seven protonation constants can be determined from the potentiometric data (log *K*_1–7_ 9.37, 8.77, 5.67, 4.57, 2.74, 1.76 and 1.05). Only four protonation constants of H_4_pyta were published previously.^16^ The species distribution diagram (Fig. S15†) and protonation scheme (Fig. S17†) of H_4_pyta are discussed in the ESI.† The overall basicity of the discussed ligands expressed as the basicity of two ring amine groups increases in the order of H_2_macropa < H_4_pyta < H_4_dota.

To get stability constants of selected Ln(iii) ions, an “out-of-cell” (“batch”) titration method had to be used as the necessary equilibration time was long. The constants are given in Table 1 (the full set of constants is listed in Table S11†) and representative distribution diagrams are shown in Fig. S19.† The complexes are fully formed above pH ∼3. HPLC was used to check the presence of the 22/21 isomers in the equilibrated solutions. In the La(iii), Ce(iii) and Nd(iii) systems, one isomer was observed. Only a small amount (<5%) of the 21 isomer was found for the Eu(iii) and Tb(iii) complexes and, thus, the stability constants obtained for these ions can be considered reasonably correct for the 22 isomer. In the case of the Er(iii) complex, solutions equilibrated at different pHs contain different, and significant, amounts of both isomers (Fig. S20†). Therefore, values of the stability constants of the complex are biased by the isomerization. Thermodynamic stability of the ^21^[Lu(pyta)]^−^ complex is significantly lower than the stability of the 22 complexes. Overall, the stability constants increase from La(iii) to Eu(iii) and then decrease. Although mutual differences in values of stability constants are small, the values point out that the Eu(iii) ion is probably the metal ion that best fits the H_4_pyta macrocyclic cavity size. This fact is in line with a trend observed for kinetic inertness (see below). The lower apparent stability constants of complexes of smaller Ln(iii) ions are thus given by an increasing abundance of the 21 isomer, but the constants cannot be quantified as the abundance of the isomers depends on the solution pH.

**Table 1:** Stability constants, log *K*_LnL_, of H_4_pyta complexes and comparison with constants of complexes of H_2_macropa, its pyridine analogues and H_4_dota (25 °C)

Ln(iii)	H_4_pyta^a^	H_4_pyta^b^^,^^c^	H_2_macropa^d^	H_2_py-macrodipa^e^	H_2_py_2_-macrodipa^f^	H_4_dota
La	24.78	22.1	14.99	14.31	16.68	∼22.9^g^
Ce	25.67		15.11	14.65	17.13	24.6^h^
Nd	25.87		14.16	14.51	17.11	∼23.0^g^
Eu	26.23	21.7 (Gd)	13.11	13.29	15.93	∼23.5^g^
Tb	25.60		11.79	11.95	14.76	∼24.5^g^
Er	24.13^i^		10.10	10.60	12.17	∼24.7^g^
Lu	23.15	21.7	8.25	11.54	11.19	26.4 (Yb)^h^

a *I* = 0.1 M (NMe_4_)Cl, this work.

b *I* = 0.1 M KCl, ref. 16.

c Protonated complexes were not included, leading to smaller values of the stability constants.

d *I* = 0.1 M KCl, ref. 8.

e *I* = 0.1 M KCl, ref. 10.

f *I* = 0.1 M KCl, ref. 11.

g *I* = 0.1 M NaCl, ref. 20.

h *I* = 0.1 M (NMe_4_)Cl, ref. 21.

i In the equilibrated solutions, different amounts of 22 and 21 isomers were simultaneously present at various pHs.

The log *K*_LnL_ values for the H_4_pyta complexes are higher than those published in the original work (La(iii) 22.1, Gd(iii) 21.7, and Lu(iii) 21.7).^16^ As the complexes are formed even in strongly acidic solutions and, therefore, the “acidic” ligand protonation constants are necessary to properly describe the metal ion–H_4_pyta systems, the values of the stability constants determined in the original work are probably incorrect. In addition, protonated complexes were not involved into chemical models, and a slow formation of the complexes was not considered in the original work. As expected, the H_4_pyta complexes are much more thermodynamically stable than those of H_2_macropa and its derivatives with the same CN due to the significantly higher basicity of the ring amino groups. Despite a lower number of donor atoms in H_4_dota complexes (CN 8), the H_4_dota complexes are only slightly less thermodynamically stable due to the much higher basicity of H_4_dota ring amino groups and high pre-organization of the ligand.

### Kinetic inertness studies

Kinetic inertness is a key parameter to be considered for complexes intended for *in vivo* utilization as it is the most decisive factor determining the fate of the complexes in the body. It is of large importance mainly for metal-based radiopharmaceuticals generally applied in tiny molar amounts, where all potentially competing metal ions and ligands present in body fluids are in a huge excess.

**Fig. 4:**
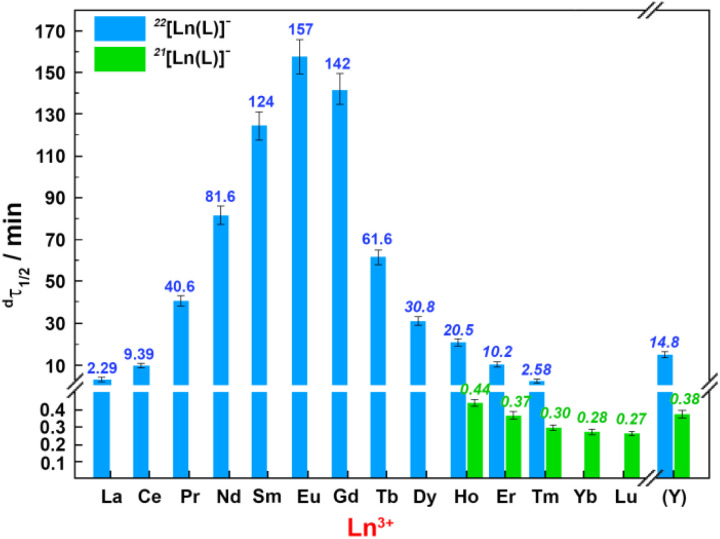
Half-lives ^d^*τ*_1/2_ (in minutes) of acid-assisted decomplexation of the [Ln(pyta)]^−^ complexes (spectrophotometry, 5.0 M HClO_4_, 90 °C). Simultaneous isomerisation and decomplexation were observed for Dy(iii)–Lu(iii) and Y(iii) complexes; their experimentally observed half-lives are in italics.

Preliminary experiments pointed to a very high kinetic inertness of the complexes even under very harsh conditions. Therefore, decomplexation of the Ln(iii) and Y(iii) complexes was followed in 5 M HClO_4_ at 90 °C. The results are compiled in Table S12† and graphically shown in Fig. 4. Examples of spectral changes and kinetic curves are given in the ESI (Fig. S21 and S22†). The complex decomposition can also be followed by HPLC (Fig. S23†), and the data obtained by spectrophotometry and HPLC are identical (Fig. S24 and Table S13†).

Decomplexation of all [Ln(pyta)]^−^ complexes was followed by spectrophotometry through a change of position of the pyridine absorption band of the free ligand (*λ*_max_: 260 nm) and the complexes (*λ*_max_: 266 nm); the 22 and 21 isomers have the same pyridine absorption. However, HPLC analysis of several manually taken samples during the decomplexation reactions showed that both pure isomers of the heavier Ln(iii) ions undergo simultaneous decomplexation and mutual isomerization. More detailed investigation of the process showed that isomerization is much more accelerated at higher temperatures and has various extents in solution with different proton concentrations. Decomplexations shown in Fig. 4 are therefore apparently “slower” for the 21 isomers due to the simultaneous formation of more inert 22 complexes and “faster” for the 22 isomers due to simultaneous conversion to more labile 21 complexes. Thus, the presented data of the kinetic inertness are only lower (22 isomers) and upper (21 isomers) estimates; however, the trend in the inertness of the 22 isomer over the lanthanide series is obvious, pointing out that the Eu(iii) complex shows the highest kinetic inertness.

As stated above, the isomerization process is more pronounced with increasing temperature. To avoid its influence, the decomplexation reactions were also followed at 50 °C where the rate of isomerization is negligible. The data show a huge difference in kinetic inertness between the 22 and 21 isomers (Tables 2, S13, S14, Fig. S23 and S24†). The 21 isomers (CN 9) decomposed significantly faster than the 22 isomers (CN 10), and this clearly shows that coordination of all pendant arms and minimalized ligand distortion increase the kinetic inertness. The decomplexation rates of the 22 isomers strongly depend on the size of the Ln(iii) ions; the Eu(iii) complex is almost three orders of magnitude more inert than the La(iii) complex. The kinetic data show, consistent with the thermodynamic results, that the size of the Eu(iii) ion best fits the H_4_pyta ligand cavity. The inertness of the isomer 21 is comparable for all Ln(iii) ions.

**Table 2:** Comparison of the observed decomplexation rate constants ^d^*k*_obs_ and half-lives for the [Ln(pyta)]^−^ complexes (5.0 M HClO_4_, 50 °C, and UV-vis); L = (pyta)^4−^

Complex	^22^[Ce(L)]^−^a	^22^[Eu(L)]^−^a	^22^[Ho(L)]^−^	^22^[Er(L)]^−^	^21^[Ho(L)]^−^	^21^[Er(L)]^−^	^21^[Yb(L)]^−^
^d^ *k*_obs_/s^−1^	3.0 × 10^−5^	4.7 × 10^−6^	2.0 × 10^−5^	3.0 × 10^−5^	2.11 × 10^−3^	2.21 × 10^−3^	2.73 × 10^−3^
^d^ *τ*_1/2_	6.4 h	41.0 h	9.6 h	6.4 h	5.5 min	5.2 min	4.2 min

a Data for 90 °C: ^22^[Ce(L)]^−^ complex: ^d^*k*_obs_ = 1.23 × 10^−3^ s^−1^ and ^d^*τ*_1/2_ = 9.39 min; ^22^[Eu(L)]^−^ complex: ^d^*k*_obs_ = 7.34 × 10^−5^ s^−1^ and ^d^*τ*_1/2_ = 157 min.

The results clearly show that the Ln(iii)–H_4_pyta complexes are among the most kinetically inert complexes described until now. In the literature, a very high kinetic inertness has been reported for Ln(iii) complexes of cross-bridged cyclam with two picolinate pendant arms where no decomplexation was observed in 2 M aq. HCl at 25 °C over 24 weeks.^22^ In the complexes, the metal ion is fully wrapped (CN 8) inside the ligand cavity of the highly pre-organized cross-bridged ligand. However, these complexes were prepared under very harsh conditions which are not compatible with radiolabelling of conjugates of the chelators with biomolecular vectors. The Ln(iii)–H_4_pyta complexes were obtained under significantly milder conditions, similar to those used for the synthesis of the Ln(iii)–H_4_dota complexes and radiopharmaceticals. Recently, the Y(iii) complex of the parent 18-py_2_N_4_ hexaazamacrocycle modified with three acetate pendant arms and one benzyl group has been studied.^23^ Its structure formally corresponds to the 21 isomer as the two coordinated acetates and benzyl group are turned toward one side of the N_6_-plane and the last acetate arm is metal-bound from the opposite side. Its kinetic inertness, *τ*_1/2_ 1.7 h in 1 M aq. HCl at 25 °C, is significantly lower than that of the H_4_pyta complexes. On the other hand, the 22 isomer of the Eu(iii) complex of the analogous tetrakis(2-hydroxyethyl) ligand 18-py_2_N_4_(HE)_4_ (Fig. 1) is also kinetically inert as no decomposition of the complex was observed after two weeks in 1 M aq. HCl at room temperature.^24^

**Fig. 5:**
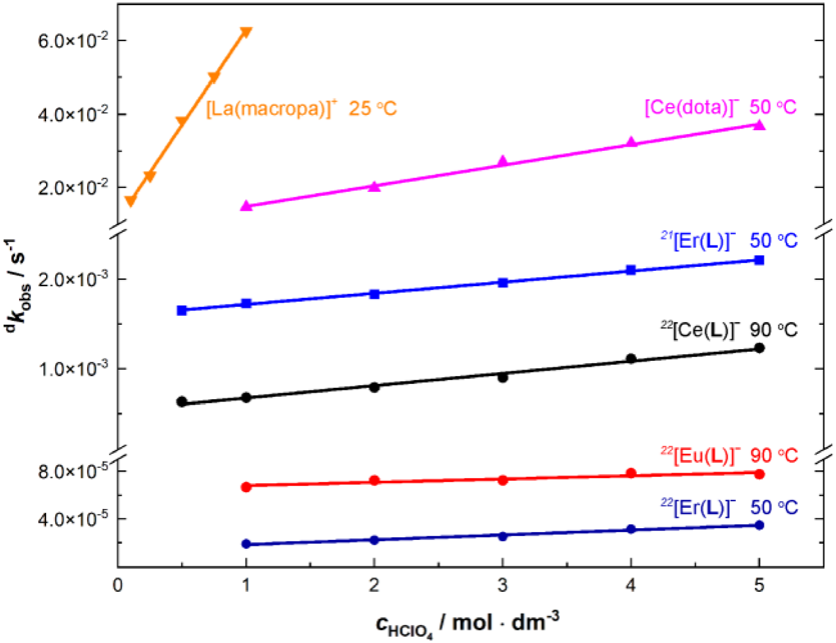
Comparison of decomplexation rates for the selected [Ln(pyta)]^−^, [Ce(dota)]^−^ and [La(macropa)]^+^ complexes at different solution acidities.

To get a better rationale for such a high kinetic inertness and to suggest a decomplexation mechanism, acid-assisted decomplexation was investigated in more detail for selected Ln(iii) complexes. Thus, the complexes of trivalent La, Ce, Eu, Er and Yb ions were studied at different acidities (0.5–5.0 M HClO_4_ or HCl, 90 °C; Table S15†) and at different temperatures (50–90 °C, 5.0 M HClO_4_; Table S14†). Examples of the experimental data are depicted in Fig. S25 and S26,† and the temperature dependencies were used to calculate decomplexation half-lives at room temperature. The observed linear dependencies of decomplexation rates on solution acidity (Fig. 5) could be successfully fitted to the most common rate law applied to the acid-assisted decomposition of complexes of macrocyclic ligands (eqn (1)).^25,26^

1 ^d^*k*_obs_ = ^d^*k*_0_ + ^d^*k*_H_·[H^+^]

**Scheme 1:**
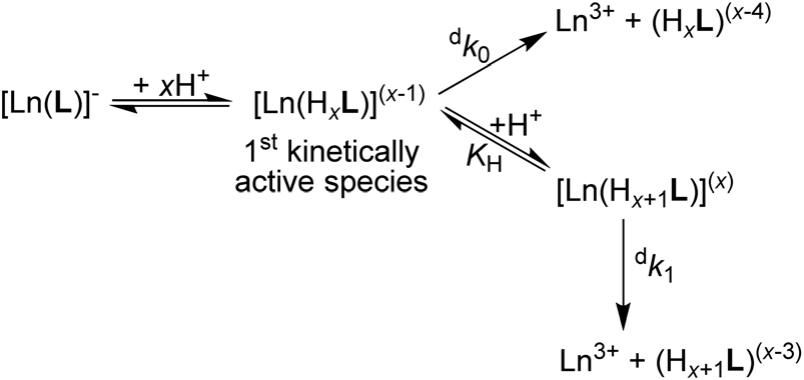
Decomplexation pathway of the [Ln(pyta)]^−^ complexes (large Ln(iii) ions) in strongly acidic solutions.

Here, ^d^*k*_0_ is a rate constant for “spontaneous” decomplexation of the first kinetically active species and ^d^*k*_H_ = *K*_H_·^d^*k*_1_ is a rate constant for decomplexation initiated by further protonation of the species (*K*_H_ is the corresponding protonation constant and ^d^*k*_1_ is the decomplexation rate constant of the corresponding protonated species; however, they cannot be individually determined from the current data). As the [Ln(pyta)]^−^ complexes decompose only in highly acidic solutions, (highly) protonated species are expected to be stable (see also below), and the kinetically active species should contain several bound protons. Then, the entire chemical process corresponding to eqn (1) can be described by Scheme 1.

Relative significance of particular complex dissociation pathways (characterized by ^d^*k*_0_ and ^d^*k*_H_) is similar for both isomers (Fig. 5 and Table S15†). Thus, we can suppose a similar decomplexation mechanism in the 22 and 21 isomers. The different inertness of the isomeric complexes can thus be attributed mainly to the different accessibility of the ring amine group for the protonation caused by dissimilar ligand conformation in the isomers (see above) and/or by the presence of the mobile non-complexed acetate pendant arm in the 21 isomer.

Decomplexation kinetics has been evaluated for several [Ln(dota)]^−^ complexes but only at room temperature.^27–29^ No kinetic data are available for Ln(iii) complexes of H_2_macropa or its derivatives. To get data for the complexes directly comparable with our results, decomplexation kinetics for [Ce(dota)]^−^ was first measured (Table 3) under conditions comparable to those for the [Ln(pyta)]^−^ complexes (1.0–5.0 M HClO_4_ and 50–90 °C), and the results are compiled in Tables S14 and S16† and shown in Fig. 5. In the 5 M acid and 50 °C, the [Ce(dota)]^−^ and ^22^[Ce(pyta)]^−^ complexes are decomposed with half-lives of ∼19 s and ∼6 h, respectively; the ^22^[Ce(pyta)]^−^ complex is about 1000-times more kinetically inert. The ^21^[Ln(pyta)]^−^ complexes with *τ*_1/2_ ∼5 min (Table 2) have about an order of magnitude higher kinetic inertness than the [Ce(dota)]^−^ complex. The decomplexation kinetic data were even more striking for the [La(macropa)]^+^ complex (Table S16†). In the 5 M acid and at 90 °C, the complex was decomposed immediately. Thus, the decomplexation was carried out in 0.1–1.0 M acid and at 25 °C leading to half-lives in the range 42–11 s, respectively; an extrapolation gives *τ*_1/2_ <2 s in 5.0 M HClO_4_ at 25 °C (Table 3). Under the same conditions, the [Ce(macropa)]^+^ complex was always fully decomposed even during a dead time of the measurement. The results clearly show that the ^22^[Ln(pyta)]^−^ complexes are extraordinarily kinetically inert, even much more than the [Ln(dota)]^−^ complexes, the current standard for highly kinetically inert complexes of lanthanide(iii) ions.

**Table 3:** Comparison of decomplexation half-times (^d^*τ*_1/2_) of the [Ln(pyta)]^−^ complexes with those of [Ce(dota)]^−^ and [La(macropa)]^+^ complexes

Conditions	Complex			
	^22^[Ce(pyta)]^−^	^22^[Eu(pyta)]^−^	[Ce(dota)]^−^	[La(macropa)]^+^
5 M HClO_4_ and 90 °C	9.39 min	157 min	2.4 s	<2 s^a^
1 M HClO_4_ and 90 °C	17.0 min	174 min	5.38 s	<2 s^a^
5 M HClO_4_ and 25 °C	3.3 days^b^	13.8 days^b^	1.8 min^b^	2.56 s^c^
1 M HClO_4_ and 25 °C	>4 days^d^	>15 days^d^	4.13 min^e^^,^^f^	11.1 s

a Decomposed during the dead time of the UV-vis measurement (about 10 s).

b Data extrapolated from the temperature dependence of *k*_obs_ (5.0 M HClO_4_ and 50–90 °C).

c Data extrapolated from *k*_obs_ measured at different acidities (0.1–1.0 M HClO_4_ and 25 °C).

d Rough estimates based on temperature and acidity dependencies.

e Measured in a separate experiment (1.0 M HClO_4_, *I* = 5.0 (H, Na)ClO_4_, and 25 °C).

f Half-times ^d^*τ*_1/2_ (1.0 M HClO_4_ and 25 °C) from the literature: [Ce(dota)]^−^ 4.62 min^29^ and [Eu(dota)]^−^ ∼3.5 h.^30^

### Protonation of the complexes

The ^22^[Ln(pyta)]^−^ complexes are highly inert even in very acidic solutions where multiply protonated complex species are to be present. However, it is impossible to determine the number of protons in the first kinetically active species (whose dissociation proceeds with the rate constant *k*_0_) from the kinetic data. Therefore, we tried to estimate the protonation state, and the corresponding protonation constant(s), of the complexes independently by potentiometric and NMR titrations. Protonation of kinetically inert complexes of macrocyclic polyazapolyacetates takes place on the coordinated carboxylate groups, and the corresponding log *K*_a_ is low (*e.g.* in the range log *K*_a_ 1–2 for the M(iii)–H_4_dota complexes).^30–32^ Two protonation constants of pre-formed [Ln(pyta)]^−^ complexes could be determined by potentiometric titrations. For the Ce(iii) complex (*i.e.* pure ^22^[Ce(pyta)]^−^ isomer), constants log *K*_1_ 2.03 and log *K*_2_ 1.74 were found. For the Lu(iii) complex (*i.e.* pure ^21^[Lu(pyta)]^−^ isomer), constants log *K*_1_ 3.60 and log *K*_2_ 1.56 were determined (for more information, see the ESI†). Values of the constants indicate protonation of the coordinated carboxylate group(s), except that for the first protonation constant of the ^21^[Lu(pyta)]^−^ complex it probably occurs on the free (non-coordinated) carboxylate group (see also Fig. 2, the solid-state structure of ^21^[Eu(Hpyta)]). These (di)protonated complexes are present in the solution above pH ∼ 1.

To check protonation in even more concentrated acids up to 10 M DCl, ^1^H NMR titration was carried out with the most inert ^22^[Eu(pyta)]^−^ complex which also provides well-resolved NMR spectra. As the shape and resolution of the spectra did not change while going to such very acidic media, the structure of the complex is preserved in the whole studied pH range. However, shifts of the signals change, mainly in acidic solutions below 0.5 M acid (Fig. S27†). It points to further protonation(s) on the coordinated carboxylate group(s), leading to tri- or tetraprotonated species on the carboxylate groups.

**Fig. 6:**
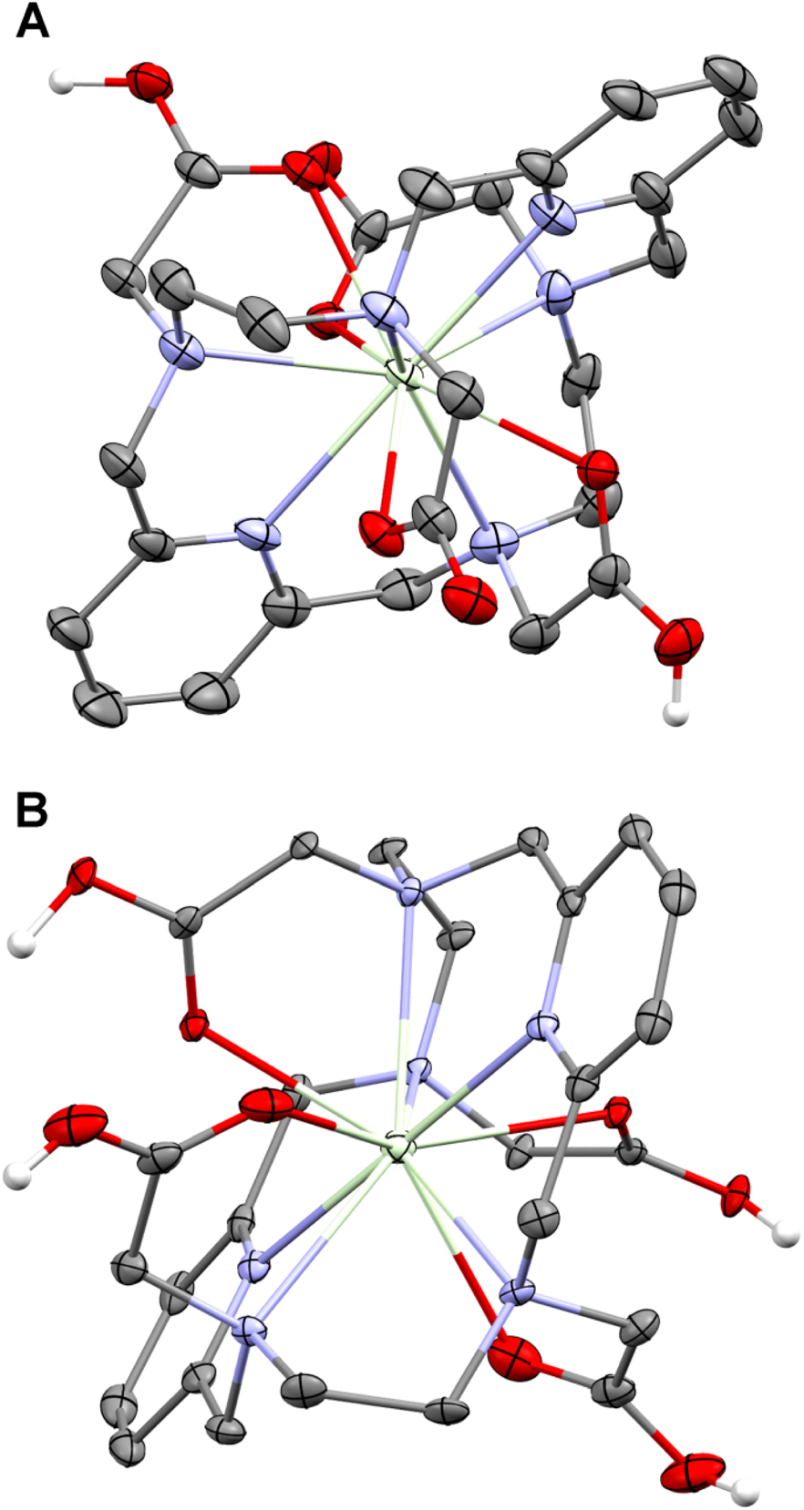
Molecular structures of the ^22^[Pr(H_2_pyta)]^+^ cation found in the crystal structure of ^22^[Pr(H_2_pyta)]Cl·5H_2_O (A) and the ^22^[Pr(H_3_pyta)]^2+^ cation present in the crystal structure of ^22^[Pr(H_3_pyta)]Cl_2_·3H_2_O; two of the O–H hydrogen atoms have half occupancy (B). Carbon-bound hydrogen atoms are omitted for clarity. Colour codes: Pr: green, O: red, N: blue, C: dark grey, and H: white. A complete atom labelling scheme is given in the ESI (Fig. S11 and S12†).

In addition, single crystals of protonated complexes were grown from acidic solutions, and their structures and protonation states were determined by X-ray diffraction. From a solution of the ^22^[Pr(pyta)]^−^ complex at pH ∼1, crystals with composition ^22^[Pr(H_2_pyta)]Cl·5H_2_O were obtained. From a solution well below pH 0 (3 M HCl), two different crystals having compositions ^22^[Pr(H_3_pyta)]Cl_2_·3H_2_O and [{Pr(H_2_O)_5_}_2_(H_4_pyta)]Cl_6_·9H_2_O were isolated. The second phase is a product of decomplexation during a long crystallization time and could be considered an out-of-cage complex as only pendant arms are coordinated to the Pr(iii) ion and ring amino groups are protonated; details are given in the ESI.† Selected geometric parameters of the complex species (Table S9†) and the crystallographic experimental data (Table S7) are given in the ESI.† The molecular structures of the di- and triprotonated complex cations are shown in Fig. 6.

In general, the molecular structures of both complex species are very similar to that of the 22 isomer of the Eu(iii) complex discussed above; the main difference is slightly longer coordination bonds due to the larger ionic radius of the Pr(iii) ion compared to the Eu(iii) ion. The ligand molecules in the [Pr(H_2_pyta)]^+^ and [Pr(H_3_pyta)]^2+^ complex species are doubly or triply, respectively, protonated on the non-coordinated oxygen atoms of the coordinated carboxylate pendant arms. In the case of ^22^[Pr(H_2_pyta)]Cl·5H_2_O, the non-protonated non-coordinated carboxylate oxygen atoms serve as the acceptors in a very strong intermolecular hydrogen bond system including protonated oxygen atoms of the neighbouring molecules (O⋯O distances are 2.461 and 2.476 Å). In the case of ^22^[Pr(H_3_L)]Cl_2_·3H_2_O, refinement of the protonation state was complicated by the crystallographic *C*_2_ symmetry of the complex species. Due to this fact, one-half of the complex molecule forms the structurally independent unit. In the independent unit, one of the non-coordinated oxygen atoms is fully protonated, whereas the second non-coordinated oxygen atom bears a half-occupied hydrogen atom. It leads to total triple-protonation of the complex species (Fig. S12†). A very short intermolecular hydrogen bond was found between the partially protonated pendant oxygen atoms from two neighbouring molecules (O⋯O 2.458 Å), forming an infinite chain of complex molecules (Fig. S13†). Thus, all protonated carboxylate groups are formally coordinated through their carbonyl groups in both solid-state structures. This fact is reflected by shorter C–O bond lengths in the C–O–Pr fragments compared to those in the C–O–H fragments (*i.e.* with the protonated oxygen atom, or those serving as the acceptor in the above-mentioned hydrogen bond system), see Table S9.† However, the Ln–O and Ln–N distances (Table S9†) are very similar in both crystal structures independent of the carboxylate protonation state and lie in the common range for such distances. These solid-state data confirm a minimal alternation of structures of the ^22^[Ln(pyta)]^−^ complexes with their protonation and suggest a possible presence of inert complex species polyprotonated on the coordinated carboxylate groups in the acidic solutions.

In complexes of macrocyclic ligands, some crystal structures showing protonated carboxylate group coordinated to a central metal ion (*i.e.* formally through its carbonyl group) have been observed.^23,33^ However, protonation of three coordinated carboxylates, or even “tetraprotonation” (if counting also the strong hydrogen bond), of one ligand was observed here for the first time. It points out that even all carboxylate groups in the ^22^[Ln(pyta)]^−^ complexes could be protonated in solution, without fast complex decomposition. Such extensive protonation of the coordinated carboxylates is also supported by the changes in ^1^H NMR spectra of the ^22^[Eu(pyta)]^−^ complex in strongly acidic solutions (see above).

### A suggestion of the decomplexation mechanism

As multiple protonation of the coordinated carboxylates occurs in solution as well as in the solid state, we can suggest a hypothesis about a mechanism of the acid-catalyzed decomplexation of the ^22^[Ln(pyta)]^−^ complexes. The complexes bind protons on the coordinated carboxylate groups, and the central metal ion in such species is still completely and symmetrically wrapped by ten ligand donor atoms. The mono- and diprotonated ^22^[Ln(H_*x*_pyta)]^0/1+^ species (*x* = 1 or 2, Scheme 1) present in moderately acidic solutions are probably not kinetically active (their decomplexation is extremely slow). In highly acidic solutions, the complexes can form tri/tetraprotonated species, ^22^[Ln(H_*x*_pyta)]^2+/3+^ (*x* = 3 or 4). It is generally accepted that the rate-determining step during the acid-assisted decomposition of polyazamacrocycle complexes is the protonation of the ring amines.^25^ The nitrogen atoms can be protonated by a proton transfer from the protonated carboxylate groups and/or by a direct attack of the amino groups. The highly positive charge of these protonated species significantly hampers access of proton(s) for the direct attack. Additionally, the amino group basicity of the title ligand is relatively low (compared with that of *e.g.* H_4_dota) and, thus, their affinity to protons is decreased, slowing down the overall decomplexation process. The symmetric “wrapping” of the metal ion by the ligand in the 22 isomer further decreases access to the amino groups as well as minimizing the ligand strain. The high number of chelate rings, the high coordination number (CN 10) as well as the presence of symmetrically arranged pyridine fragments also contribute to the rigidity of the species. On the other hand, the lower number of chelate rings (CN 9) coupled with the larger ligand twist should decrease the kinetic inertness of the 21 isomer. In the 21 isomer, the flexible non-coordinated and protonated pendant arm helps to transfer protons to more exposed macrocycle nitrogen atoms in the distorted structure. Consequently, decomplexation is faster.

## Conclusions

During investigation of Ln(iii) complexes of H_4_pyta, we observed the formation of isomers with CN 10 and CN 9 depending on the size of lanthanide(iii) ions. The ten-coordinated 22 isomers are highly symmetrical, and the ligand almost ideally wraps the metal ion by ten chelate rings leading to very sturdy complexes. It is demonstrated by the exceptional kinetic inertness of the complexes, which is significantly higher than the inertness of the [Ln(dota)]^−^ complexes. It is coupled with a relatively easy formation of the complexes. Recently, even more inert Ln(iii) complexes of an unsymmetrical H_2_do2a-derived ligand with a “bridge” over the Ln(iii) ion have been described.^34^ However, the rigidifying “bridge” was introduced by click chemistry after the metal ion complexation, and such complex synthesis is more complicated than that with H_4_pyta. According to the data, the size of trivalent europium best fits the ligand cavity. Even the complex of the largest lanthanide(iii) ion, La(iii), is highly kinetically inert. The high kinetic inertness of the 22 isomer should be preserved under other conditions as well, *e.g.* under *in vivo* conditions, as a full decomplexation (by any mechanism) requires simultaneous removal of ten chelate rings, and such a process is generally highly improbable. The kinetic inertness of the 21 isomer is significantly lower than the other one, but it is still comparable with the kinetic inertness of the [Ln(dota)]^−^ complexes. Finally, La(iii) is considered a surrogate of Ac(iii), and the kinetic inertness could be expected even for this very large metal ion.

The other aspect important for the radiochemistry of the title ligand, its derivatives and other 18-membered rings with coordinating pendant arms is the possible mutual 22/21 isomerization of the complexes. Isomerization should be considered during radiolabelling commonly performed at higher temperatures and in slightly acidic solutions. As the mutual interconversion of the isomers was observed for smaller lanthanide(iii) ions under various conditions and, mainly, at high temperatures, complexes of these ligands should be applied in radiochemistry after a careful evaluation of the isomerism with “cold” complexes.

The ligand can also be suitable for other large metal ions from the bottom of the periodic table, as recently confirmed for Pb(ii).^19^ We suppose that the easy formation and the enormous kinetic inertness will establish H_4_pyta as a “standard” ligand for large metal ions requiring CN 10. H_4_pyta can be considered a parent ligand of a new family of rigid chelators with large ligand cavities, which will be suitable for applications in molecular imaging, radiochemistry and nuclear medicine.

## Author contributions

J. F. – syntheses of the ligand/complexes, characterization of ligand/complexes, single crystal preparation, kinetic measurements, kinetic evaluation, manuscript writing; P. U. – synthesis and characterization of complexes, NMR measurements; V. K. – study concept, kinetic and potentiometric data evaluation, manuscript writing; J. H. – equilibrium studies, potentiometric data evaluation; I. C. – X-ray data acquisition, X-ray data fitting; J. K. – X-ray data fitting and interpretation, manuscript writing; P. H. – study concept, money acquisition, manuscript writing.

## Conflicts of interest

There are no conflicts to declare.

## Supplementary Material

SC-OLF-D5SC01893E-s001

SC-OLF-D5SC01893E-s002

## Data Availability

Crystallographic data for the compounds have been deposited at the CCDC (https://www.ccdc.cam.ac.uk/). The data supporting this article have been included as part of the ESI.†
