# Supplementary material for: Extraordinary kinetic inertness of lanthanide(iii) complexes of pyridine-rigidified 18-membered hexaazamacrocycles with four acetate pendant arms
Source: Chem Sci. 2025 May 30;16(27):12558–67. doi: 10.1039/d5sc01893e (PMC12153104; doi:10.1039/d5sc01893e)
Supplement: SC-016-D5SC01893E-s001 [file SC-016-D5SC01893E-s001.pdf]

Supplementary Information (SI) for Chemical Science.
This journal is © The Royal Society of Chemistry 2025

Supporting Information for

Extraordinary inertness of lanthanide(III) complexes of pyridine-rigidified 18-membered hexaazamacrocycle with four acetate pendant arms

Jan Faltejsek, Peter Urbanovský, Vojtěch Kubíček, Jana Havlíčková, Ivana Císařová, Jan Kotek, Petr Hermann*

Department of Inorganic Chemistry, Faculty of Science, Universita Karlova (Charles University), Hlavova 2030, 12843 Prague 2, Czech Republic. Tel: +420-22195-1263. E-mail: petrh@natur.cuni.cz

Content:

Syntheses and characterisations

General methods S2

Chromatographic separations (**Tables S1–S4**) S2–S3

Ligand synthesis (**Scheme S1**) S4–S6

Synthesis of complexes S6

Figure S1. HPLC chromatograms of the isomers and free ligand S6

Figure S2. HPLC retention times of the prepared isomeric complexes S7

Table S5. Analytical HPLC-UV-MS characterisation of isomers of complexes S7

Table S6. The ^{1}H NMR signals of selected ^{22}[Ln(pyta)]$^{-}$ complexes S8

Figure S3. Selected ^{1}H NMR spectra of pure isomers of the [Ln(pyta)]$^{-}$ complexes S9

Preparative HPLC separation of the isomers S10

Mutual isomerisation of the complexes S10

Figure S4. Time course of mutual isomerisation *22* and *21* isomers of the Er(III)–H_4pyta complex at pH 2 and 7 at 25 °C. S11

Solid-state structures – X-ray diffraction

Preparation of single crystals of the complexes S12

Experimental details on X-ray diffraction data fitting S12–S14

Table S7. Experimental crystal parameters S15

Table S8. Geometric parameters of hydrogen bonds in the solid-state structures of macrocycles S16

Table S9. Selected structural parameters found in the solid-state structures of complexes S17–S18

Figure S5. Molecular structure of 18-py$_2$N$_4$ S19

Figure S6. Molecular structure of H_4pyta S19

Figure S7. Molecular structure of (H_8**L**)$^{4+}$ cation S20

Figure S8. Molecular structures of the ^{22}[Eu(pyta)]$^{-}$ (CN 10) and ^{21}[Eu(Hpyta)] (CN 9) species S20

Figure S9. Crystal packing found in the crystal structure of ^{21}[Eu(Hpyta)]·3H_2O S21

Figure S10. Crystal packing found in the crystal structure of Na^{220}[Eu(pyta)]·13.5H_2O S21

Figure S11. Molecular structure of the ^{22}[Pr(H_2pyta)]$^{+}$ cation S23

Figure S12. Molecular structure of the ^{22}[Pr(H_3pyta)]$^{2+}$ cation S23

Figure S13. Chain {^{22}[Pr(H_3pyta)]$^{2+}$}$_n$ found in the crystal structure of ^{22}[Pr(H_3pyta)]Cl_2·3H_2O S24

Description of the solid-state structure of [{Pr(H_2O)$_5$}$_2$(H_4pyta)]Cl_6·9H_2O (**Figure S14**) S25

Equilibrium studies

Determination of protonation and stability constants – Experimental S26

Table S10. Protonation constants, $\beta_{\mathrm{H}h\mathrm{L}}$ and $\log K_a$, of H_4pyta; comparison with other ligands S27
Figure S15. Distribution diagram of H_4pyta S27
Discussion of H_4pyta protonation scheme S27–S28
Figure S16. The ^{1}H NMR titration of H_4pyta S28
Figure S17. Suggested protonation scheme of H_4pyta S28
Figure S18. The UV titration of H_4pyta S29
Table S11. Overall, $\beta_{\mathrm{H}h\mathrm{ML}}$, and derived, $\log K_a$ stability constants of Ln(III)–H_4pyta complexes S29
Figure S19. Distribution diagrams of Ce(III) (*22* isomer) and Lu(III) (*21* isomer) complexes of H_4pyta S29
Figure S20. Abundance of *22* / *21* isomers in the Er(III)–H_4pyta system in the equilibrated solutions S30
Protonation constants of the pre-formed complexes S30
Kinetic studies
UV-vis spectrophotometry S31
HPLC S32
Evaluation of the decomplexation kinetic data S32–S33
Table S12. Observed decomplexation rate constants $^{d}k_{obs}$ and half-lives $^{d}\tau_{½}$ of Ln(III)–H_4pyta complexes (5.0 M $HClO_4$, 90 °C) S34
Figure S21. Examples of spectral changes in the course of the decomplexation reactions (5.0 M $HClO_4$, 90 °C): ^{22}[Ce(pyta)]$^-$, ^{22}[Eu(pyta)]$^-$ and ^{21}[Yb(pyta)]$^-$ complexes. S35
Figure S22. Examples of change in absorbance in the course ofdecomplexation reactions of ^{22}[Er(pyta)]$^-$ and ^{21}[Er(pyta)]$^-$ complexes (5.0 M $HClO_4$, 90 °C) S35
Figure S23. Changes of spectral intensities and of HPLC chromatograms in the course of the decomplexation reaction (5.0 M $HClO_4$, 90 / 50 °C) S36
Figure S24. Experimentally determined abundances of the ^{22}[Ce(pyta)]$^-$ and ^{21}[Yb(pyta)]$^-$ complexes during decomplexation reaction using UV-Vis and HPLC (5.0 M $HClO_4$) S37
Table S13. Observed decomplexation rate constants $^{d}k_{obs}$ and half-lives $\tau_{½}$ of [Ln(pyta)]$^-$ complexes by UV-Vis and HPLC (5.0 M $HClO_4$; 50 or 90 °C) S37
Table S14. Observed decomplexation rate constants $^{d}k_{obs}$ and conditional activation parameters of the selected Ln(III)–H_4pyta and [Ce(H_2O)(dota)]$^-$ at different temperatures (5.0 M $HClO_4$) S38
Table S15. Observed decomplexation rate constants $^{d}k_{obs}$ of the Ln(III)–H_4pyta complexes at different acidities (90 °C) S38
Figure S25. Examples of fits of decomplexation data at different temperatures: ^{22}[Ce(pyta)]$^-$, ^{220}[Eu(pyta)]$^-$ and ^{211}[Yb(pyta)]$^-$ S39
Figure S26. Examples of decomplexation data for the ^{22}[Ce(pyta)]$^-$, ^{22}[Eu(pyta)]$^-$ and ^{21}[Yb(pyta)]$^-$ complexes at different acidities S39
Table S16. Decomplexation data for [Ce(H_2O)(dota)]$^-$ and [La(macropa)]$^+$ S40
Figure S27. The ^{1}H NMR titration of ^{22}[Eu(pyta)]$^-$ complex in highly acidic solutions S40
References S41

Syntheses and characterisations

General methods

All commercially available starting materials were used without further purification. Pyridine-2,6-dicarbaldehyde was synthesised according to previously published procedures.[1] The H_2macropa was a generous gift from Dr. C. Mamat (HZDR, Dresden, Germany). The H_4dota was purchased from CheMatech (France).

The ^{1}H (400 MHz)/^{13}C (100 MHz) NMR spectra of organic compounds were acquired at 25 °C (if not mentioned otherwise) on a Bruker 400 Avance III spectrometer, and the ^{1}H (300 MHz) NMR spectra of the complexes were obtained using a Varian S300 spectrometer. The NMR measurements were performed either in D_2O or $CDCl_3$. Chemical shifts were referenced to *t*-BuOH (δ_H 1.25 ppm, δ_C 30.3 ppm) for measurements in D_2O or to residual $CHCl_3$ (δ_H 7.26 ppm, δ_C 77.2 ppm) for $CDCl_3$ solutions. Broadband ^{1}H decoupling was used during ^{13}C spectra acquisition. Values of chemical shifts are given in ppm and the coupling constants are in Hz. The spectra were processed in the MestReNova program. The ESI-MS experiments were performed on a Waters Acquity QDa spectrometer with a quadrupole mass analyser in negative and positive modes (range 30–1250 *m/z*). Only dominant peaks assigned to the products are given. Elemental analyses were carried out at IOCB ASCR (Prague); C/H/N was determined on the PE 2400 II device and the other elements were obtained via X-ray fluorescence analysis on the SPECTRO iQ II device. Merck aluminium foils with silica gel 60 F-254 were used for TLC. The UV-vis spectra were obtained using a Specord 50 Plus (Analytic Jena AG) spectrophotometer (220–400 nm) with temperature maintained by the Peltier Block.

Chromatographic separations

Automated column chromatography at medium pressure ("flash" chromatography) was performed on a Sepacore (Büchi) equipment assembly consisting of a C-640 UV spectrometer, C-620 control unit, C-660 fraction collector, two C-605 pumps and four lamps with optionally adjustable wavelengths (200 nm, 210 nm, 254 nm and 264 nm were used). The column for final purification of H_4pyta (C18-AQ silica gel, 120 g, Sepacore, 4.2×22 cm, Büchi) was thoroughly washed with the 0.1% aq. HCl solution before each separation. The separation method is shown in Table S1. The fractions of 50-mL were collected.

The analytical HPLC of the complexes was carried out on the Waters Acquity QDa device using silica gel C18-AQ column Cortec STM-C18 (4.6×50 mm, particle diameter 2.7 μm) employing methods listed in Tables S2 and S3.

To separate isomers of the complexes, preparative HPLC block (2535 Quaternary gradient module, preparative degasser block, 2489 UV/Visible detector and FlexInject; all Waters) and column (Luna C18(2)-AQ silica gel, 250×21.2 mm, 100 Å, Phenomenex) were used. The chromatography was followed at 266 nm (absorption maximum of the complexes). The system was thoroughly washed with 0.1% aq. TFA solution before each separation. The separation method is shown in Table S4.

Table S1. The method G1 used for the “flash” preparative separation of H_4**L**.

$t_1 \rightarrow t_2$ [min]		0.1% HCl in H_2O [%]	0.1% HCl in ACN [%]
$0 \rightarrow 3$	isocratic	100	0
$3 \rightarrow 23$	gradient	$0 \rightarrow 100$	$100 \rightarrow 0$
$23 \rightarrow 25$	isocratic	0	100

Acetonitrile (Rotisolv, HPLC Gradient Grade), deionized water (Milli-Q, Millipore), flow rate: 12 ml/min.

Table S2. The method G2 used for analytical HPLC separation of H_4pyta and the $[Ln(pyta)]^-$ complexes.

t_1 / min $\rightarrow t_2$ / min		0.1% TFA in H_2O [%]	0.1% TFA in ACN [%]
$0 \rightarrow 2$	isocratic	100	0
$2 \rightarrow 8$	gradient	$100 \rightarrow 0$	$0 \rightarrow 100$
$8 \rightarrow 11$	isocratic	0	100
$11 \rightarrow 13$	gradient	$0 \rightarrow 100$	$100 \rightarrow 0$
$13 \rightarrow 15$	isocratic	100	0

Acetonitrile (Rotisolv, HPLC Gradient Grade), deionized water (Milli-Q, Millipore), flow rate: 1.2 ml/min.

Table S3. The method G3 used for analytical HPLC separation of the $[Ln(pyta)]^-$ complexes.

t_1 / min $\rightarrow t_2$ / min		0.1% TFA in H_2O [%]	0.1% TFA in ACN [%]
$0 \rightarrow 2$	isocratic	100	0
$2 \rightarrow 8$	gradient	$100 \rightarrow 90$	$0 \rightarrow 10$
$8 \rightarrow 11$	gradient	$90 \rightarrow 0$	$10 \rightarrow 100$
$11 \rightarrow 13$	gradient	$0 \rightarrow 100$	$100 \rightarrow 0$
$13 \rightarrow 15$	isocratic	100	0

Acetonitrile (Rotisolv, HPLC Gradient Grade), deionized water (Milli-Q, Millipore), flow rate: 1.2 ml/min.

Table S4. The method G4 used for preparative HPLC separation of the $[Ln(pyta)]^-$ complexes.

t_1 / min $\rightarrow t_2$ / min		0.1% TFA in H_2O [%]	0.1% TFA in ACN [%]
$0 \rightarrow 5$	isocratic	100	0
$5 \rightarrow 30$	gradient	$100 \rightarrow 90$	$0 \rightarrow 10$
$30 \rightarrow 35$	gradient	$90 \rightarrow 0$	$10 \rightarrow 100$
$35 \rightarrow 37$	isocratic	0	100

Acetonitrile (Rotisolv, HPLC Gradient Grade), deionized water (Milli-Q, Millipore), flow rate: 12 ml/min.

Syntheses

The parent 18-membered polyazamacrocycle[2] and H_4pyta[3] were prepared by the modified published procedures according to Scheme S1. Briefly, the 18-membered Schiff base was obtained by reaction of pyridine-2,6-dicarbaldehyde and ethylenediamine using a La(III)-template ($LaCl_3$) in water as a solvent (room temperature; concentrations - 0.04 M La(III), 0.08 M reactants) followed by $NaBH_4$ reduction. The La(III) ion was removed by precipitation as hydroxide and the macrocyclic amine was extracted into CH_2Cl_2. The synthesis was scaled up to ~10 grams of the macrocycle with a good yield and purity. Utilizing the La(III) cation in the template macrocyclization was proven to be more convenient than using the Ba(II) cation. In the original paper,[3] H_4pyta was prepared as hydrochloride by simple evaporation in a significantly lower overall yield.

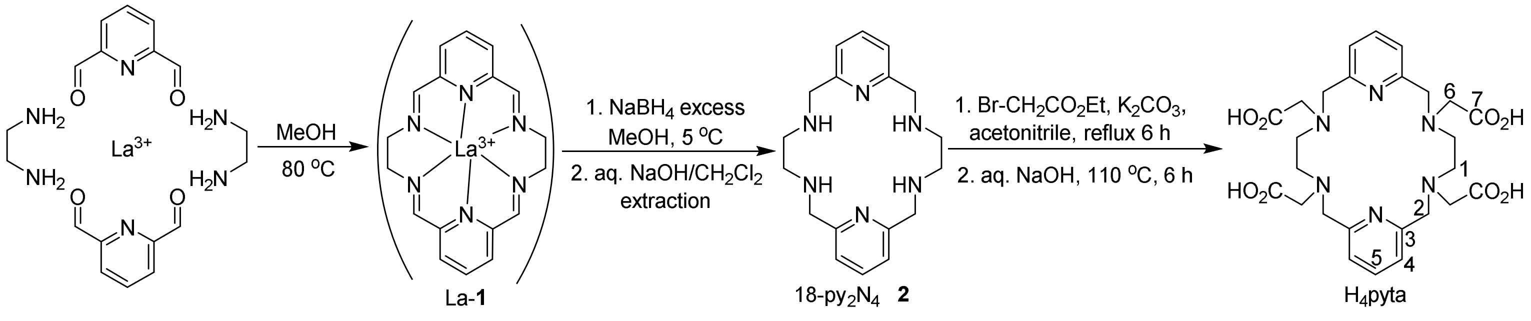

Scheme S1. Synthesis of H_4pyta.

<u>*Template synthesis of 18-py$_2$N$_4$ macrocycle (**2**)*</u>

In a round-bottom flask (500 mL), pyridine-2,6-dicarbaldehyde (2.60 g, 19.3 mmol, 1 equiv.) and $La(NO_3)_3·6H_2O$ (4.17 g, 9.63 mmol, 0.5 equiv.) were dissolved in MeOH (250 ml) at room temperature. Ethylenediamine (1.16 g, 19.3 mmol, 1 equiv.) was slowly added dropwise to the solution over approximately 3 min with continuous stirring. The reaction mixture was then heated to 80 °C for 4 h. After cooling to room temperature, the flask containing the heterogeneous mixture was placed in a NaCl/ice cooling bath. Then, portions (about 0.5 g) of $NaBH_4$ (in total 2.20 g, 57.8 mmol, 3 equiv.) were added to the reaction mixture in such a way that the temperature of the reaction mixture did not exceed 5 °C. After 30 min of stirring, a new portion of $NaBH_4$ (1.10 g, 28.9 mmol, 1.5 equiv.) was added to the reaction mixture. After 4 h at ~0 °C, the resulting homogeneous solution was evaporated to dryness under reduced pressure. The residue was taken up in a 20% (w/w) aq. NaOH (ca 250 mL) and the resulting white precipitate was removed by centrifugation (3000 rpm) for 15 min. The precipitate was then re-suspended in another portion of 20% NaOH (ca 100 mL) and the solid phase was separated again by centrifugation. Compound **2** was extracted from the combined alkaline aqueous solutions using dichloromethane (5×100 mL). After drying the combined organic fractions with anhydrous Na_2SO_4 and filtration of the solids, the filtrate was evaporated to dryness *in vacuo*. Compound **2**·$3H_2O$ was obtained as a white powder (3.11 g, 85% yield) and was directly used in the next step. It was observed that the product gradually decomposes when stored for several days under ambient conditions (temperature, air). For long-term storage, this compound was dissolved in conc. aq. HCl (5 mL) and the excess HCl was removed on rotavapor resulting in a white powder with the formula **2**·4HCl·$3H_2O$ (4.26 g, 84%). Elemental analysis: **2**·$3H_2O$: found (calc. for $C_{18}H_{26}N_6·3H_2O$): C 56.69 (56.82), H 8.46 (8.48), N 21.98 (22.09); **2**·4HCl·$3H_2O$: found (calc. for $C_{18}H_{26}N_6·4HCl·4H_2O$): C 41.01 (41.08), H 6.95 (6.89), N 16.02 (15.97), Cl 26.87 (26.94).

Single crystals of **2**·4H_2O were obtained by a slow evaporation of wet CH_2Cl_2 solution. For a discussion of the solid-state structure, see below.

Synthesis of H_4pyta

The macrocyclic amine **2**·3H_2O prepared above (1.14 g, 3.0 mmol) was dissolved in anhydrous acetonitrile (200 mL) and fine-powdered anhydrous K_2CO_3 (2.54 g, 24 mmol, 8 equiv.) was added. A solution of ethyl bromoacetate (2.65 mL, 24 mmol, 8 equiv.) in dry acetonitrile (50 mL) was added dropwise to the stirred mixture over 15 min. With constant stirring, the suspension was heated to reflux for 6 h. After cooling, the mixture was filtered through a fine frit and the filtrate was evaporated to dryness *in vacuo*. The residue was dissolved in $CHCl_3$ (approx. 200 mL) and the solution was thoroughly extracted with water (4×100 mL). The organic phase was dried (Na_2SO_4), the solids filtered off and the filtrate was evaporated under reduced pressure to afford a yellow oil containing Et$_4$**L**. The crude product was dissolved in *i*PrOH (20 mL) and purified by a "flash" chromatography (silica gel, 4.2×15 cm, 80 g, *i*PrOH:MeOH 20:1). The fractions containing pure tetraethyl-ester were identified by MS, combined and concentrated under reduced pressure to give a yellow oil (1.53 g, 84 %) which solidified on standing.

^{1}H NMR (400 MHz, $CDCl_3$): δ 7.62 (t, 2H, $^3J_{HH}$ 7.6 Hz, H5), 7.17 (d, 4H, $^3J_{HH}$ 7.6 Hz, H4), 3.95 (s, 8H, H2), 3.91 (q, 8H, $^3J_{HH}$ 6.9 Hz, H8), 3.15 (s, 8H, H6), 2.83 (s, 8H, H1) 1.12 (t, 12H, $^3J_{HH}$ 7.0 Hz, H9). ^{13}C{^{1}H} NMR (100 MHz, $CDCl_3$): δ 171.8 (C7), 157.8 (C3), 138.3 (C5), 123.1 (C4), 60.8 (C_{ester}), 57.4 (C2), 56.0 (C6), 52.5 (C1), 14.1 (C_{ester}). MS (+) found (calc.): 671.55 (671.38 $[M+H]^+$); 693.51 (693.36 $[M+Na]^+$).

The ester was suspended in water (150 mL) and NaOH (0.46 g, 11.5 mmol, 5 equiv.) was added. The mixture was heated to reflux at 110 °C for 6 h. Then, the solution was stirred at room temperature for 48 h. The reaction mixture was concentrated *in vacuo* to ca 25 mL. The solution was applied onto a Dowex 50 Wx4 (100 mL) column in the H^+-form. The column was washed with water (approx. 150 mL) and the product was eluted with 50% aq. pyridine. The eluate was concentrated under reduced pressure. The residue – a yellowish oil – was dissolved in water (10 mL), the resulting mixture was filtered through a PVDF microfilter (0.22 μm) and the product was purified by a "flash" chromatography (C18-silica, 120 g, method G1). Fractions containing the pure product (UV, MS) were combined and concentrated *in vacuo*. The residue was dissolved in MeOH (50 mL), the product was precipitated by the addition of acetone (150 mL) utilizing sonification (5 min) and the solid was filtered off. The solids was dried by dispersing in Et_2O (10 mL), filtration and drying on the air to give H_4pyta·4HCl·4H_2O as an off-white powder (1.84 g, 79 %; purity >95 % by HPLC-UV/vis-MS, method G2). Elemental analysis: found (calc. for $C_{26}H_{34}N_6O_8$·4HCl·4H_2O): C 39.67 (40.22), H 5.61 (5.97), N 10.39 (10.82), Cl 17.9 (18.26). This form was used for the preparation of the Ln(III) complexes.

To prepare the stock solutions of H_4pyta in zwitterionic form for potentiometry, kinetic and some NMR measurements, the H_4pyta hydrochloride (~1 g) was dissolved in water, the slightly yellow solution was decolourised with charcoal. The filtered colourless solution was applied onto Dowex 50 Wx4 (100 mL) column. The column was washed with water (~200 mL) until a neutral pH of the eluate and the product was eluted with 50% aq. pyridine. The pyridine eluate was concentrated under reduced pressure. The residue was dissolved in water (10 mL) and the product was crystallised on the zwitterionic form by layering the solution with acetone. The crystalline material was collected by filtration and dried in the air (H_4pyta·4H_2O, purity >98 % by HPLC-UV/vis-MS, method G2). Elemental analysis: found (calc. for $C_{26}H_{34}N_6O_8$·4H_2O): C 55.71 (55.91), H 6.49 (6.14), N 15.01 (15.05).

Single crystals of H_4pyta·$8H_2O$ were obtained by diffusion of acetone vapours into a diluted aqueous solution of zwitterionic H_4pyta. Single crystals of the ligand hydrochloride, (H_8pyta)Cl_4·$3H_2O$, were prepared by diffusion of acetone vapours into a solution of H_4pyta in 5% aq. HCl. For a discussion of the solid-state structures, see below.

^{1}H NMR (400 MHz, D_2O, pD 5.9): δ 8.10 (t, 2H, $^3J_{HH}$ 7.8 Hz, H5), 7.60 (d, 4H, $^3J_{HH}$ 7.8 Hz, H4), 4.48 (s, 8H, H2), 3.53 (s, 8H, H6), 3.52 (s, 8H, H1). ^{13}C{^{1}H} NMR (100 MHz, D_2O, pD 5.9): δ 173.9 (C7), 152.2 (C3), 142.3 (C5), 125.7 (C4), 58.1 (C2), 56.9 (C6), 51.9 (C1). MS (+) found (calc.): 559.22 (559.25 $[M+H]^+$), 581.22 (581.23 $[M+Na]^+$). Analytical HPLC-UV/Vis-MS (C18, method G2): 1.10 min.

Synthesis of the Ln(III) complexes

The H_4pyta·4HCl·$4H_2O$ (62 mg, 0.08 mmol) and the $LnCl_3$ hydrate (1.1 equiv.) were dissolved in deionized water (15 mL) and 1 M aq. NaOH was slowly added dropwise until pH ~6.5 was reached. The reaction mixture was stirred at 60 °C overnight. Then pH was re-adjusted to 6.5 and the mixture was shortly heated. The procedure was repeated until the pH remained stable. The excess of Ln(III) was precipitated on addition of aq. NaOH to pH > ~10. The precipitated $Ln(OH)_3$ was filtered off using a syringe PVDF microfilter (0.22 μm). The reaction mixture was analysed using HPLC-UV/Vis-MS (quantification via the UV detection at 266 nm, regardless of the isomer, Methods G2 and G3) to get *22*/*21* isomer abundance (Figures 3, S1 and S2; Table S5). Retention times of each isomer monotonously increase for smaller Ln(III) ions probably pointing to a more compact and, thus, more hydrophobic structures for the smaller ions. Reaction mixtures containing pure *22* isomer (La–Nd, *22* isomer) and those containing significant majority of one isomer (*22* isomer for Sm–Tb; *21* isomer for Yb and Lu; see Figure 3) were precipitated as hydrochloride salts after slight acidification of the solutions to pH 3–4 by a slow addition of acetone. The precipitate was filtered off and washed with acetone. The solids were dried on the air, and checked by HPLC to determine a possible presence of the minor isomer. and the isolated complexes were further characterised by MS and ^{1}H NMR (Tables S5 and S6, Figure S3). The ^{1}H NMR spectra of the Ln(III) complexes matched the published data.[3] These solids contained only one isomer which was the major one in the original solution.

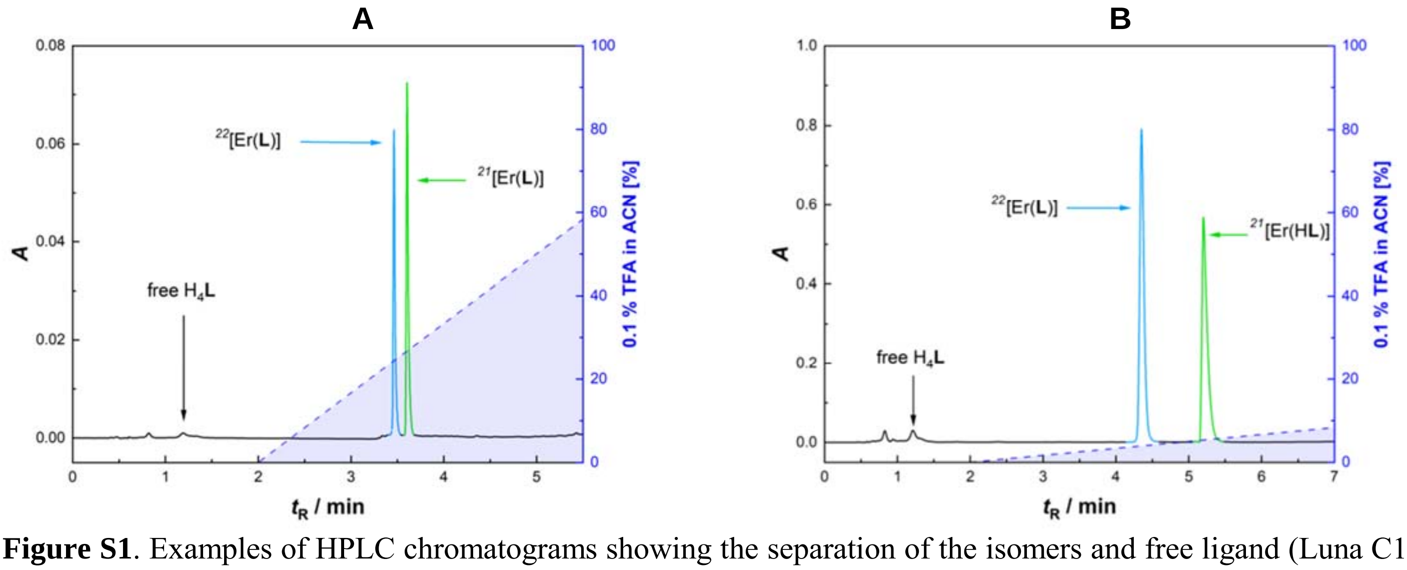

Figure S1. Examples of HPLC chromatograms showing the separation of the isomers and free ligand (Luna C18-AQ, Phenomenex, 5×25 mm). **A**: method G2; **B**: method G3.

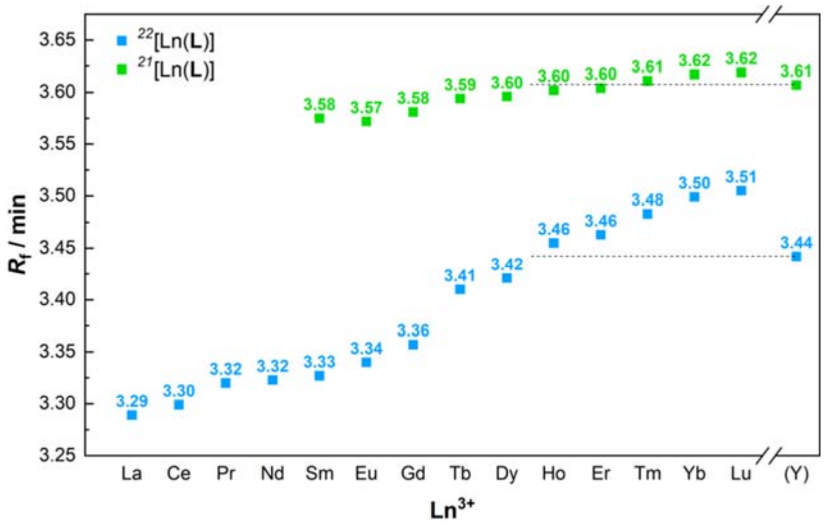

Figure S2. The HPLC retention times of the prepared complexes; Method G2 (for the conditions, see the description of the HPLC measurements). The data points for the *22* isomers are in **blue** and the data points for the *21* isomers are in **green**.

Table S5. Analytical HPLC-UV-MS characterisation of the isomers of Ln(III)–H_4pyta complexes. The MS signals had expected isotopic patterns (only masses of ions containing the most abundant isotopes are given). **L** = $(pyta)^{4-}$

Ln^{3+}	MS(+) found (calc.)[a]	MS(−) found (calc.)[b]	^{22}[Ln(**L**)]$^-$ Anal. HPLC (G2)	^{21}[Ln(**L**)]$^-$ Anal. HPLC (G2)
La	695.4 (695.13)	921.4 (921.10)<br>1035.3 (1035.10)	3.29 min	–[c]
Ce	696.5 (696.13)	922.3 (922.10)<br>1036.3 (1036.10)<br>1150.3 (1150.09)	3.30 min	–[c]
Pr	697.4 (697.14)	923.4 (923.11)<br>1037.4 (1037.10)	3.32 min	–[c]
Nd	700.4 (700.14)	926.3 (926.11)<br>1040.4 (1040.10)	3.32 min	–[c]
Sm	708.5 (708.15)	934.3 (934.12)<br>1048.3 (1048.11)	3.33 min	3.58 min
Eu	709.5 (709.15)	935.2 (935.12)<br>1049.4 (1049.11)	3.34 min	3.57 min
Gd	714.4 (714.15)	940.3 (940.12)<br>1054.3 (1054.12)	3.36 min	3.58 min
Tb	715.3 (715.15)	1055.3 (1055.12)<br>1168.9 (1169.11)	3.41 min	3.59 min
Dy	720.3 (720.16)	946.3 (946.13)<br>1060.8 (1060.12)<br>1173.9 (1173.13)	3.42 min	3.60 min
Ho	721.3 (721.16)	947.4 (947.13)<br>1061.4 (1061.12)	3.46 min	3.60 min
Er	724.0 (724.16)	949.8 (950.13)<br>1063.4 (1063.12)	3.46 min	3.60 min
Tm	725.3 (725.16)	951.4 (951.13)<br>1065.9 (1065.13)	3.48 min	3.61 min
Yb	730.2 (730.17)	not detected	3.50 min	3.62 min
Lu	731.3 (731.17)	not detected	3.51 min	3.62 min
Y	645.4 (645.15)	871.3 (871.10)<br>985.2 (985.10)	3.44 min	3.61 min

[a]MS(+): $[Ln(H_2\mathbf{L})]^+$. [b]MS(−): $[Ln(H_2\mathbf{L})+2CF_3CO_2]^-$, $[Ln(H_3\mathbf{L})+3CF_3CO_2]^-$ and/or $[Ln(H_4\mathbf{L})+4CF_3CO_2]^-$, respectively. [c]The *21* isomer was not observed.

Table S6. The ^{1}H NMR signals of selected ^{22}[Ln(pyta)]$^-$ complexes (D_2O, pD 5.9 (Ce, Eu) or 2.9 (Er), 25 °C, 300 MHz); 8 signals are present (D_2 molecular symmetry). Because of the C_1 molecular symmetry of the ^{21}[Ln(pyta)]$^-$ complexes, they exhibit up to 30 signals which are not fully resolved and, therefore, examples are not listed.

Complex	^{1}H NMR, δ_H, ppm
^{22}[Ce(**L**)]$^-$	15.50 (s, 4H), 13.55 (s, 4H), 12.45 (s, 4H), 8.84 (d, 4H, H_{py}, $^3J_{HH}$ 8.9 Hz), 8.05 (pseudo-t, 2H, $H_{py\text{-}4}$, $^3J_{HH}$ 7.6 Hz), 7.71 (d, 4H_{py}, $^3J_{HH}$ 7.9 Hz), 2.43 (d, 4H, $^2J_{HH}$ 7.9 Hz), −7.24 (s, 4H)
^{22}[Eu(**L**)]$^-$	15.96 (s, 4H), 4.13 (pseudo-t, 2H, $H_{py\text{-}4}$, $^3J_{HH}$ 8.0 Hz), 1.66 (s, 4H), 0.19 (d, 4H, H_{py}, $^3J_{HH}$ 10.9 Hz), −1.79 (s, 4H), −5.92 (s, 4H), −12.69 (s, 4H), −17.29 (s, 4H)
^{22}[Er(**L**)]$^-$	51.7 (s, 4H), 45.9 (s, 4H), 22.5–19.8 (m), 19.8–15.5 (m), 8.3 (s, 4H), −43.2 (s, 4H), −76.4 (s, 4H), −112.5 (s, 4H)

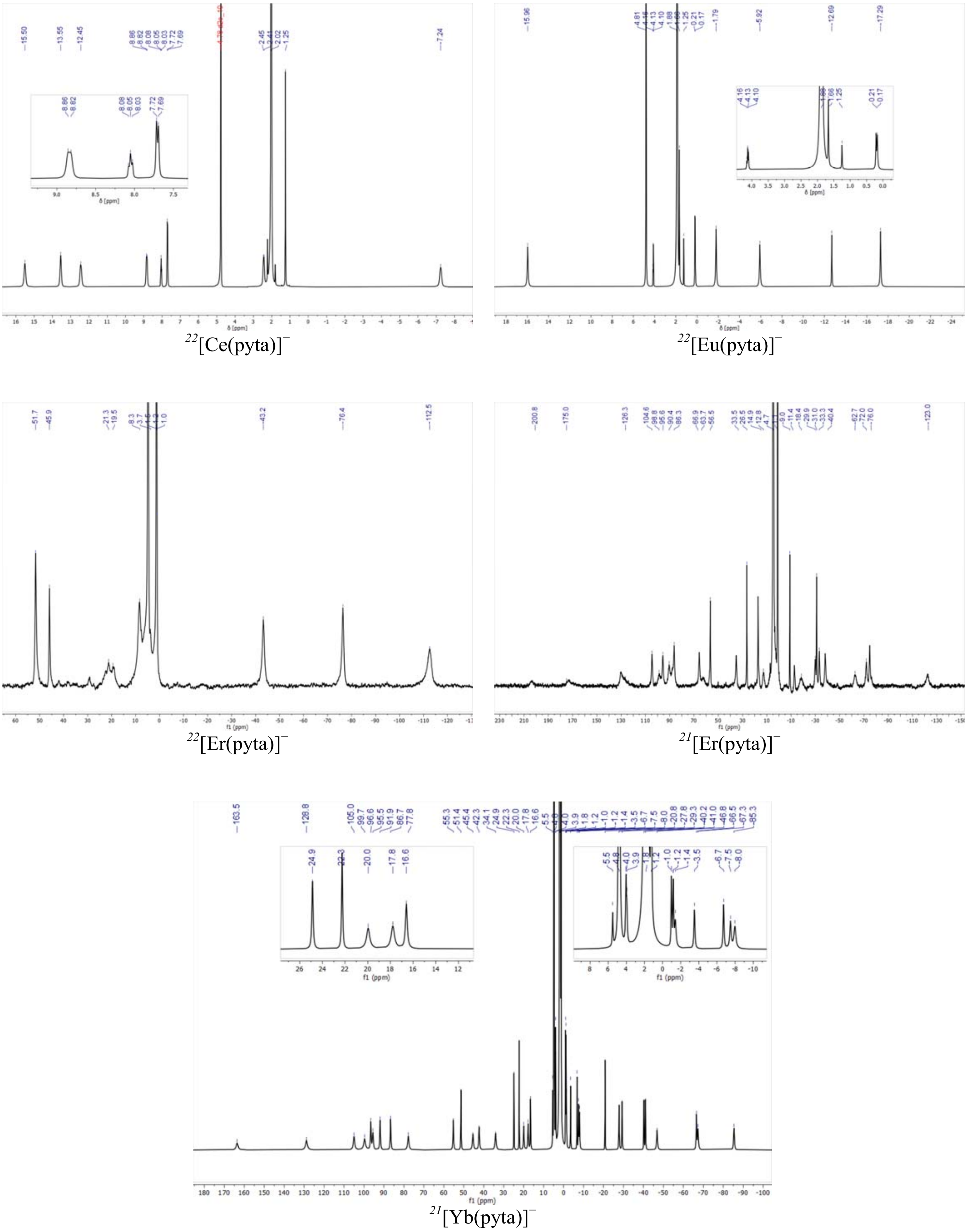

Figure S3. Selected ^{1}H NMR spectra of pure isomers of the $[Ln(pyta)]^-$ complexes (D_2O, pD ~5.9–6.0 (Ce, Eu, Yb) or ~2.9 (Er), 300 MHz).

Preparative HPLC separation of the isomers

In the case of the Dy(III)–Tm(III) and Y(III) complexes, both *22* and *21* isomers were present in the reaction mixtures in significant amounts. A simple precipitation of the isomer mixtures solution led to mixtures of the isomers in the solid state. Therefore, the isomers were separated by preparative RP-HPLC (C18-AQ silica, 250×21.2 mm, 100 Å, 0.1% aq. TFA:ACN, method G4). The reaction mixtures were evaporated to dryness and samples (~50 mg) were loaded onto the column. Fractions from all separations containing pure isomers were combined and volatiles were partially evaporated *in vacuo* at a low temperature (t <30 °C) to avoid re-isomerization of the pure isomers in acidic solution (presence of TFA). The concentrated solutions were diluted with water (final volume ~250 mL, final pH ~3) and lyophilised to give trifluoroacetate salts of isomers as fluffy solids. The products were characterised by MS and ^{1}H NMR (for example, see Figure S3). In the solid phase, the isomers of all complexes are fully stable and do not undergo isomerisation. In aqueous solutions, the complexes slowly isomerise (weeks), depending on the pH of the solutions – the isomerisation is faster in acid solutions (see below).

Mutual isomerisation of the complexes

To study mutual isomer transformation, isomers of $[Er(pyta)]^{-}$ were selected as the equilibrium mixture contains both isomers with a similar abundance (Figure 3). The isolated solid isomers (~20 mg) were dissolved in aq. buffer solutions (1.5 mL; *N,N,N*-trimethyl-glycine (TMG), pH 2; or 3-(*N*-morpholino)-propane-sulfonic acid (MOPS), pH 7; buffer concentrations were 0.1 M) and isomerisation was monitored at 25 °C using HPLC. Samples were taken off in the appropriate time points and were immediately analysed using HPLC-UV/Vis-MS (C18-AQ silica gel, column 4.6×50 mm, particle size 2.7 µm, mobile phase 0.1% aq. TFA:ACN, gradient G3). The kinetic traces are shown in Figure S4. The isomerisation proceeds slowly and the equilibrium was reached after approximately 300 h at pH 2. However, the process was significantly slower at higher pH and the equilibrium was not reached at pH 7 even after ~500 h. At pH 7, complex $^{22}[Er(pyta)]^{-}$ almost did not undergo isomerisation (~2 % of $^{21}[Er(pyta)]^{-}$ was present at the end of the experiments). On the contrary, the complex $^{21}[Er(pyta)]^{-}$ somewhat isomerises to the $^{22}[Er(pyta)]^{-}$ complex, but very slowly.

Starting isomer	pH 2	pH 7
22	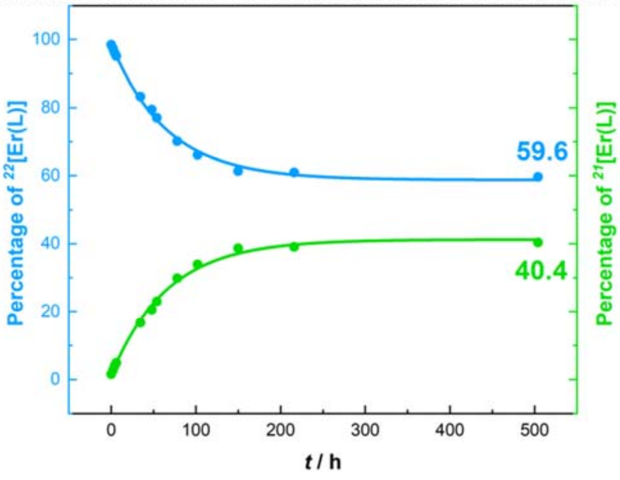	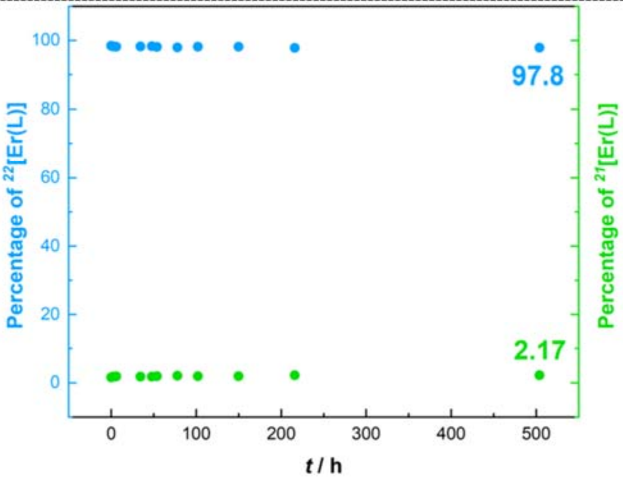
21	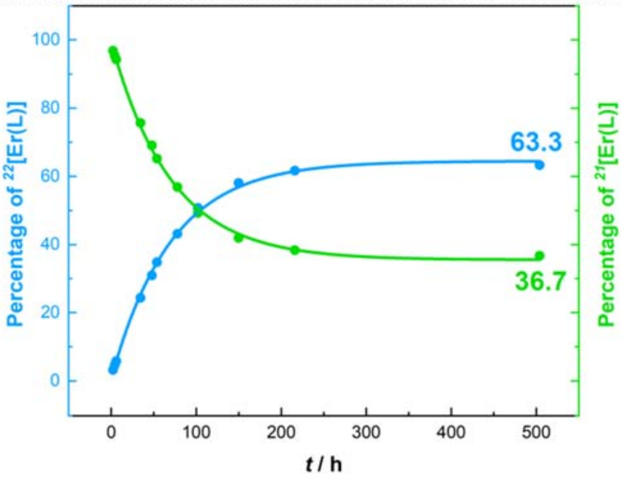	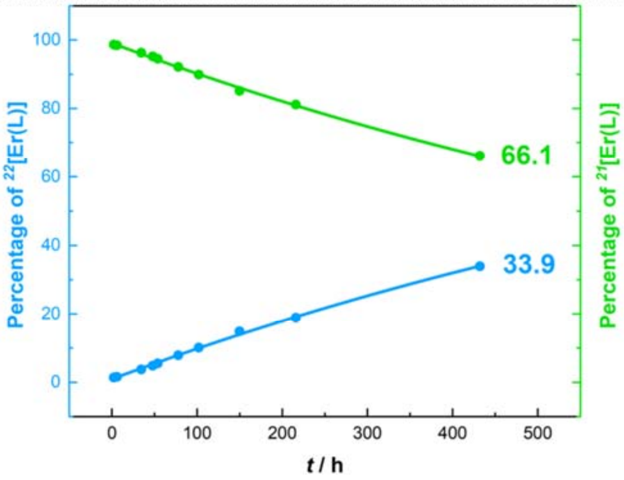

Figure S4. Time course of mutual isomerisation *22* (top) and *21* (bottom) isomers of the Er(III)–H_4pyta complex at pH 2 (left) and 7 (right) at 25 °C. The data points for the *22* isomers are in **blue**, and the data points for the *21* isomers are in **green**. The curves are used to guide the eyes.

Solid-state structures – X-ray diffraction

Preparations of single crystals of 18-py$_2$N$_4$·4H$_2$O, H$_4$pyta·8H$_2$O and (H$_8$pyta)Cl$_4$·3H$_2$O suitable for X-ray diffraction studies are reported in the Syntheses part (see above).

The single crystals of the complexes were prepared in the following ways:

(*i*) The mother liquor after precipitation of the pure *22*-Eu(III) complex was evaporated to dryness *in vacuo*. The residue was dissolved in a minimum amount of water, the solution was carefully overlaid with the same volume of acetone and the mixture was left to crystallise at –5 °C (freezer). After several days, the crystallisation produced several crystals of ^{21}[Eu(Hpyta)]·3H$_2$O.

(*ii*) The pure solid *22*-Eu(III) complex was dissolved in a minimum amount of water, the pH was increased to ~6 by diluted aq. NaOH and the solution was carefully overlaid with the same volume of acetone. The mixture was left to crystallise at room temperature in a closed vessel. Crystals with composition Na^{22}[Eu(pyta)]·13.5H$_2$O were obtained.

(*iii*) The solid *22*-Pr(III) complex was dissolved in a minimum amount of water and the solution was acidified by diluted aq. HCl to pH ~0. A slow diffusion of acetone vapours at room temperature over several days produced crystals of ^{22}[Pr(H$_2$pyta)]Cl·5H$_2$O.

(*iv*) To get single crystals of the triprotonated complex, the solid *22*-Pr(III) complex was dissolved in ~3 M aq. HCl and the solution was cooled to 0 °C. A slow diffusion of acetone vapours at 0–5 °C (fridge; to slow down any decomplexation process) over several weeks produced two differently shaped crystals with compositions ^{22}[Pr(H$_3$pyta)]Cl$_2$·3H$_2$O (major part) and [{Pr(H$_2$O)$_5$}$_2$(H$_4$pyta)]Cl$_6$·9H$_2$O (minor part).

The selected crystals were mounted on a glass fibre in a random orientation and the diffraction data were collected by Bruker D8 VENTURE Duo diffractometer at 120 K with a micro-focus sealed tube using Cu-K$_\alpha$ radiation (λ 1.54178 Å) for the crystal of (H$_8$pyta)Cl$_4$·3H$_2$O or using Mo-K$_\alpha$ radiation (λ 0.71073 Å) for all other structures. Data were analysed using the SAINT software package (SAINT V8.40B, Bruker AXS Inc., 2019). Data were corrected for absorption effects using the multi-scan method (SADABS).[4] All structures were solved by direct methods (SHELXT2018)[5] and refined using full-matrix least-squares techniques (SHELXL2017).[6]

In general, all non-hydrogen atoms were refined anisotropically. The hydrogen atoms were localised in the electron density map; however, those bound to the carbon atoms were placed in theoretical positions using U_{eq}(H) = 1.2 U_{eq}(C) to keep the number of parameters low and only the hydrogen atoms bound to heteroatoms (N, O) were fully refined. Some hydrogen atoms belonging to the O–H or N–H groups were fixed in original or theoretical positions if their full refinement led to unrealistically short or long bonding distances. For an overview of experimental crystallographic data see Table S7. Structural data for the organic molecules, mainly the geometry of selected hydrogen bonds relevant for discussing the protonation scheme (see below) is compiled in Table S8. Selected structural parameters for complexes are listed in Table S9.

In the crystal structure of 18-py$_2$N$_4$·4H$_2$O, the parent polyazamacrocycle molecule possesses C_{2h} symmetry, so one-quarter of the formula unit forms a structurally independent part with two halves of water molecules. All heteroatom-attached hydrogen atoms were fully refined. The molecular structure of 18-py$_2$N$_4$ is shown in Figure S5.

In the crystal structure of the zwitterionic form of the ligand, H$_4$pyta·8H$_2$O, two independent ligand molecules form the asymmetric part of the unit cell. In the structure, a large disorder between water molecules of crystallization was found which could not be reliably modelled and, thus, Platon SQUEEZE[7] was used to subtract the corresponding

electron density. It gave 32 water molecules per unit cell, corresponding to 8 water molecules per ligand molecule. The total number of water molecules roughly corresponded to the number obtained if the disorder was modelled using a large number of partly occupied water molecules. Both independent ligand molecules have very similar geometry (as reflected also by a similar intramolecular hydrogen bond system, see Table S8) and, thus, the molecular structure of only one of them is shown in Figure S6.

In the crystal structure of $(H_8pyta)Cl_4{\cdot}3H_2O$, one-half of the formula unit forms the structurally independent part. The ligand molecule possesses the centre of symmetry. Similarly to the previous structure, a disorder of water molecules of crystallization was found and it was treated using Platon SQUEEZE.[7] It gave 12 water molecules per unit cell, corresponding to 3 molecules per ligand molecule. The total number of water molecules roughly corresponded to the number obtained if the disorder was modelled using a large number of partly occupied water molecules. Molecular structure of the $(H_8pyta)^{4+}$ cation is shown in Figure S7.

In the crystal structure of ^{21}[Eu(Hpyta)]·3H_2O, two structurally independent complex molecules were present together with 6 molecules of crystallization. So, formally, the independent part is to double the formula unit. Both complex molecules have very similar geometries (Table S9). A slight disorder was found in both complex molecules. In the first one, one of the non-coordinated carboxylate oxygen atoms was split into two positions. In the other molecule, one whole pendant arm was found disordered, including the pivot nitrogen atom. The occupancies of the individual positions were 60:40%. Molecular structure of one of the independent molecules is shown in the main text (Figure 2) and here (Figure S8) with the numbering scheme. Figure 9 shows a part of the crystal packing of ^{21}[Eu(Hpyta)]·3H_2O.

In the crystal structure of Na^{22}[Eu(pyta)]·13.5H_2O, several water molecules of crystallization were found disordered. The occupancies of some of them were fixed to reach similar thermal factors of all residues and total occupancy of one molecule over several very close positions. The hydrogen atoms belonging to such water molecules could not be located in the electron density map. Molecular structure of the $[Eu(pyta)]^{-}$ anion is shown in the main text (Figure 2) and here (Figure S8) with the numbering scheme. Figure 10 shows a connection of two neighbouring units through coordination to the Na(I) ion.

In the crystal structure of $^{22}[Pr(H_2pyta)]Cl{\cdot}5H_2O$, the structurally independent part one corresponds to the formula unit. A large disorder between water molecules of crystallization was observed. Positions of the chloride anions were also non-fully occupied. Therefore, some of the water molecules were treated by Platon SQUEEZE.[7] The chloride anion was modelled disordered over several positions. However, the protonation state of the complex fragment was unambiguously proved as hydrogen atoms belonging to the carboxylate moieties could be fully refined. Molecular structure of the $^{22}[Pr(H_2pyta)]^{+}$ complex cation is shown in Figure S11.

In the crystal structure of $^{22}[Pr(H_3pyta)]Cl_2{\cdot}3H_2O$, one-half of the formula unit forms the structurally independent part. The complex molecule poses a two-fold symmetry axis. Molecular structure of the $^{22}[Pr(H_3pyta)]^{2+}$ complex cation is shown in Figure S12. One of the pendant arms in the independent part is fully protonated whereas the proton on the other pendant arm is half-occupied and serves as a hydrogen-bond bridge with the symmetry-related pendant arm from the neighbouring molecule. So, one proton is split over two close positions, each half belonging to different neighbouring molecule. It leads to a chain of complex molecules connected by hydrogen bonds; a part of the chain is shown in Figure S13. Such symmetrical sharing of the proton in the intermolecular hydrogen bond system

leads to the unusual triple protonation state even in the case of the crystallographic C_2 symmetry of the complex species.

In the crystal structure of $[\{Pr(H_2O)_5\}_2(H_4pyta)]Cl_6{\cdot}9H_2O$, one-half of the formula unit forms the structurally independent part. The ligand molecule possesses a centre of symmetry. Several disordered water molecules were found. They were treated split into several positions; hydrogen atoms of such water molecules could not be localised. The coordination sphere of the Pr(III) ion is formed by oxygen atoms of the carboxylate pendant arms of three ligand molecules – one is coordinated in the κ-*O*,*O*' mode, other two are bound in a monodentate fashion. It leads to a complicated polymer structure, a part of which is shown in Figure S14.

Table S7. Crystal parameters obtained by single-crystal X-ray diffraction of 18-py$_2$N$_4$·4H$_2$O, H$_4$pyta·8H$_2$O, (H$_8$pyta)Cl$_4$·3H$_2$O, ^{21}[Eu(Hpyta)]·3H$_2$O, Na^{20}[Eu(pyta)]·13.5H$_2$O, ^{22}[Pr(H$_2$pyta)]Cl·5H$_2$O, ^{22}[Pr(H$_3$pyta)]Cl$_2$·3H$_2$O and [{Pr(H$_2$O)$_5$}$_2$(H$_4$pyta)]Cl$_6$·9H$_2$O (**L** = (pyta)$^{4-}$).

Compound	18-py$_2$N$_4$·4H$_2$O	H$_4$**L**·8H$_2$O	(H$_8$**L**)Cl$_4$·3H$_2$O	^{21}[Eu(H**L**)]·3H$_2$O	Na^{22}[Eu(**L**)]·13.5H$_2$O	^{22}[Pr(H$_2$**L**)]Cl·5H$_2$O	^{22}[Pr(H$_3$**L**)]Cl$_2$·3H$_2$O	[{Pr(H$_2$O)$_5$}$_2$(H$_4$**L**)]Cl$_6$·9H$_2$O
Formula	$C_{18}H_{34}N_6O_4$	$C_{26}H_{50}N_6O_{16}$	$C_{26}H_{44}Cl_4N_6O_{11}$	$C_{26}H_{37}EuN_6O_{11}$	$C_{26}H_{57}EuN_6NaO_{21.5}$	$C_{26}H_{42}ClN_6O_{13}Pr$	$C_{26}H_{39}Cl_2N_6O_{11}Pr$	$C_{26}H_{72}Cl_6N_6O_{27}Pr_2$
M_w	398.51	702.72	758.47	761.57	972.72	823.01	823.44	1395.41
Crystal system	orthorhombic	triclinic	monoclinic	monoclinic	triclinic	monoclinic	monoclinic	triclinic
Space group	*Pbam*	*P*−1	*C*2/*c*	*P*2$_1$/*n*	*P*−1	C2/*c*	*C*2/*c*	*P*−1
a / Å	21.9337(5)	12.6858(5)	22.1760(8)	11.1441(15)	9.8498(8)	19.8316(16)	16.5314(6)	9.9445(6)
b / Å	5.42370(10)	12.9121(4)	12.8958(5)	37.436(5)	14.4593(10)	19.2254(14)	19.3002(7)	11.2448(5)
c / Å	9.0915(2)	20.6862(8)	12.8625(5)	14.4231(19)	15.2054(13)	19.0053(15)	9.6305(4)	13.1854(8)
α / °	90	93.5600(10)	90	90	71.824(3)	90	90	94.789(2)
β / °	90	96.977(2)	109.2360(10)	90.594(4)	84.744(3)	95.647(3)	93.3480(10)	109.861(2)
γ / °	90	90.5620(10)	90	90	85.628(3)	90	90	99.596(2)
U / Å^3	1081.54(4)	3356.3(2)	3473.0(2)	6016.80(14)	2046.3(3)	7211.01(10)	3067.5(2)	1351.64(13)
Z	2	4	4	8	2	8	4	1
Unique refl.	1307	15433	3420	13869	9459	8277	3520	6196
Obsd. refl.	1254	12164	3207	12612	9120	7530	3441	4985
$R(I>2\sigma(I))$	0.0341	0.0543	0.0457	0.0631	0.0397	0.0549	0.0426	0.0480
R'(all data)	0.0356	0.0678	0.0479	0.0707	0.0410	0.0607	0.0439	0.0659
$wR(I>2\sigma(I))$	0.0903	0.1440	0.1439	0.1647	0.1084	0.1318	0.1228	0.1193
wR'(all data)	0.0938	0.1523	0.1463	0.1726	0.1103	0.1358	0.1237	0.1300
CCDC ref. no.	2422578	2422576	2422574	2422579	2422580	2422577	2422575	2422573

Table S8. Geometric parameters of selected hydrogen bonds in the solid-state structures of macrocycles.

d(D–H)$^{\#}$ / Å	d(H···A)$^{\#}$ / Å	d(D···A) / Å	∠ (D–H···A)$^{\#}$ / °
	18-py$_2$N$_4$·4H$_2$O		
d(N4–H41) = 0.89(1)	d(H41···N1) = 2.45(1)	d(N4···N1) = 2.822(1)	∠ (N4–H41···N1) = 105.1(9)
	H$_4$**L**·8H$_2$O, molecule A		
d(N4A–H41) =1.00	d(H41···N1A) = 2.32	d(N4A···N1A) =2.799(2)	∠ (N4A–H41···N1A) =108.1
	d(H41···O511) = 1.89	d(N4A···O511) =2.833(2)	∠ (N4A–H41···O511) =157.1
d(N7A–H71) =1.00	d(H71···N10A) = 2.38	d(N7A···N10A) =2.815(2)	∠ (N7A–H71···N10A) =105.1
	d(H71···O611) = 1.94	d(N7A···O611) =2.870(2)	∠ (N7A–H71···O611) =152.7
d(N13A–H131) = 1.00	d(H131···O611) = 2.00	d(N13A···O611) =2.973(2)	∠ (N13A–H131···O611) =162.2
d(N16A–H161) = 1.00	d(H161···O511) = 1.95	d(N16A···O511) =2.906(2)	∠ (N16A–H161···O511) =159.5
	H$_4$pyta·8H$_2$O, molecule B		
d(N4B–H45) = 1.00	d(H45···N1B) = 2.32	d(N4B···N1B) = 2.794(2)	∠ (N4B–H45···N1B) = 107.6
	d(H45···O515) = 1.86	d(N4B···O515) = 2.805(2)	∠ (N4B–H45···O515) = 156.9
d(N7B–H75) = 1.00	d(H75···N10B) = 2.32	d(N7B···N10B) = 2.785(3)	∠ (N7B–H75···N10B) = 107.5
	d(H75···O615) = 1.88	d(N7B···O615) = 2.812(2)	∠ (N7B–H75···O615) = 154.3
d(N13B–H135) = 1.00	d(H135···O615) = 1.91	d(N13B···O615) = 2.880(2)	∠ (N13B–H135···O615) = 161.6
d(N16B–H165) = 1.00	d(H165···O515) = 2.00	d(N16B···O515) = 2.965(2)	∠ (N16B–H165···O515) = 161.4
	(H$_8$pyta)Cl$_4$·3H$_2$O		
d(N4–H41) = 0.96	d(H41···N1) = 2.52	d(N4···N1) = 2.904(1)	∠ (N4–H41···N1) = 103.9
d(N7–H71) = 0.96	d(H71···N1$^{\$}$) = 2.50	d(N7···N1$^{\$}$) = 2.861(1)	∠ (N7–H71···N1$^{\$}$) = 102.0

$^{\#}$ If no estimated standard deviations are given, the position of the hydrogen atom was fixed. $^{\$}$ Symmetry-related atom: $-x+1/2$, $-y+3/2$, $-z+1$.

Table S9. Selected structural parameters found for the ^{21}[Eu(Hpyta)], ^{22}[Eu(pyta)]$^{-}$, ^{22}[Pr(H_2pyta)]$^{+}$ and ^{22}[Pr(H_3pyta)]$^{2+}$ complex species in the solid-state structures of ^{21}[Eu(Hpyta)]·3H_2O, Na^{22}[Eu(pyta)]·13.5H_2O, ^{22}[Pr(H_2pyta)]Cl·5H_2O and ^{22}[Pr(H_3pyta)]Cl_2·3H_2O, respectively.

Parameter	^{21}[Eu(Hpyta)]·3H_2O Molecule A	^{21}[Eu(Hpyta)]·3H_2O Molecule B	Na^{22}[Eu(pyta)]·13.5H_2O	^{22}[Pr(H_2pyta)]Cl·5H_2O	^{22}[Pr(H_3pyta)]Cl_2·3H_2O[$]
Coordination bonds, Å					
Ln1–N1(py)[@]	2.569(6)	2.581(6)	2.581(3)	2.602(5)	2.613(4)
Ln1–N4(am)[@]	2.641(7)	2.642(7)	2.640(3)	2.668(5)	2.683(4)
Ln1–N7(am)[@]	2.688(8)	2.654(7)	2.650(3)	2.684(5)	2.683(4)[$]
Ln1–N10(py)[@]	2.553(7)	2.563(8)	2.597(3)	2.617(5)	2.613(4)[$]
Ln1–N13(am)[@]	2.631(8)	2.603(8)	2.683(3)	2.681(5)	2.689(4)[$]
Ln1–N16(am)[@]	2.688(7)	2.698(16)[¥]	2.682(3)	2.683(5)	2.689(4)[$]
Ln1–O311(pendant on N4)	2.340(6)	2.335(7)	2.498(3)	2.594(4)	2.645(4)[$]
Ln1–O411(pendant on N7)	2.331(7)	2.339(7)	2.545(3)	2.532(4)	2.645(4)[$]
Ln1–O511(pendant on N13)	2.327(6)	2.353(7)	2.470(3)	2.547(4)	2.518(3)[$]
Ln1–O611(pendant on N16)	–	–	2.460(3)	2.564(4)	2.518(3)[$]
Angles, °					
∠ N1(py)-Ln-N10(py)	148.0(2)	147.2(3)	179.28(10)	179.30(17)	179.64(16)[$]
∠ py1–py2 planes[£]	59.1(3)	60.7(3)	21.4(2)	18.83(17)	19.4(2)
C–O bond lengths, Å					
Coordinated oxygen atoms					
C31–O311	1.269(11)	1.260(12)[§]	1.286(4)	1.244(7)	1.251(6)[$]
C41–O411	1.273(11)	1.298(12)[§]	1.274(5)	1.264(7)	1.251(6)[$]
C51–O511	1.278(11)	1.265(12)[§]	1.258(5)	1.250(7)	1.248(5)[$]
C61–O611	–	–	1.259(5)	1.235(7)	1.248(5)[$]
Non-coordinated oxygen atoms					
C61–O611	1.286(14)*	1.310(19)*[¥§]	–	–	–
C31–O312	1.296(17)	1.241(12)[§]	1.233(5)	1.281(7)*	1.272(6)[$]
C61–O412	1.238(12)	1.212(13)[§]	1.253(5)	1.251(8)	1.272(6)[$]
C41–O512	1.255(11)	1.233(13)[§]	1.251(5)	1.274(6)	1.278(5)[$*&]
C51–O612	1.227(13)	1.224(22)[¥§]	1.258(5)	1.287(7)*	1.278(5)[$*&]

[@] N(py) – nitrogen atom of pyridine unit, N(am) – nitrogen atom of aliphatic amino group. [£] py = pyridine. [$] The molecule has C_2-symmetry, the symmetry axis connects the centres of the CH_2–CH_2 bonds of the ethylenediamine fragments and passes through the central Pr^{3+} ion (corresponding transformation # = $-x + 1, y, -z + ½$); atoms corresponding to labels used in the Table: N7(am) = N4#, N10(py) = N1#, N13(am) = N7#, N16(am) = N7, O311 = O211, O411 = O211#, O511 = O211#, O611 = O311, O312 = O212, O412 = O212#, O512 = O312#, O612 = O312. * Protonated oxygen atom. [&] Partially (half) occupied hydrogen atom due to crystallographic symmetry. [§] Labelling of the oxygen atoms of the molecule B: O311 = O315, O411 = O415, O511 = O515, O611 = O615, O312 = O316, O412 = O416, O512 = O516, O612= O616. [¥] Position of the disordered atom with higher occupancy.

Table S9. Selected structural parameters found for the [21][Eu(Hpyta)], [22][Eu(pyta)]$^{-}$, [22][Pr(H_2pyta)]$^{+}$ and [22][Pr(H_3pyta)]$^{2+}$ complex species in the solid state structures of [21][Eu(Hpyta)]·$3H_2O$, Na[22][Eu(pyta)]·13.5H_2O, [22][Pr(H_2pyta)]Cl·5H_2O and [22][Pr(H_3pyta)]Cl_2·3H_2O, respectively – continuation.

Parameter	[21][Eu(Hpyta)]·3H_2O Molecule A	[21][Eu(Hpyta)]·3H_2O Molecule B	Na[22][Eu(pyta)]·13.5H_2O	[22][Pr(H_2pyta)]Cl·5H_2O	[22][Pr(H_3pyta)]Cl_2·3$H_2O$$^{\$}$
Distances/deviations from mean N_6-plane, Å					
N1(py)–N_6-plane$^{@£}$	−0.187(6)	−0.285(8)$^{¥}$	0.015(3)	0.000(4)	−0.001(3)
N4(am)–N_6-plane$^{@£}$	−0.897(6)	−0.847(7)$^{¥}$	−0.923(3)	−0.889(4)	−0.890(3)
N7(am)–N_6-plane$^{@£}$	1.337(6)	1.362(7)$^{¥}$	0.910(3)	0.883(4)	0.890(3)
N10(py)–N_6-plane$^{@£}$	−0.527(6)	−0.514(7)$^{¥}$	0.018(2)	0.006(4)	0.001(3)
N13(am)–N_6-plane$^{@£}$	−0.439(6)	−0.496(8)$^{¥}$	−0.915(2)	−0.886(4)	−0.888(3)
N16(am)–N_6-plane$^{@£}$	0.712(6)	0.781(8)$^{¥}$	0.896(3)	0.886(4)	0.888(3)
Ln1–N_6-plane$^{£}$	0.301(3)	0.275(4)$^{¥}$	0.0099(13)	−0.010(2)	0
Macrocycle torsion angles, °					
C18–N1–C2–C3	178.4(8)	178.7(9)	177.0(4)	177.9(5)	174.2(4)
N1–C2–C3–N4	43.2(1.1)	38.5(1.2)	29.3(5)	30.9(7)	30.7(6)
C2–C3–N4–C5	−173.1(7)	−171.2(8)	−164.4(3)	−167.6(5)	−166.8(4)
C3–N4–C5–C6	69.8(9)	71.4(9)	83.7(4)	81.6(6)	82.9(5)
N4–C5–C6–N7	63.6(1.0)	65.1(1.0)	56.6(5)	56.8(7)	55.0(7)
C5–C6–N7–C8	82.5(9)	81.6(9)	82.8(4)	83.6(6)	82.9(5)
C6–N7–C8–C9	−99.9(9)	−100.6(9)	−166.2(3)	−171.1(5)	−166.8(4)
N7–C8–C9–N10	−31.1(1.1)	−29.6(1.2)	31.3(5)	41.3(8)	30.7(6)
C8–C9–N10–C11	−168.5(8)	−168.3(8)	175.0(3)	177.3(6)	174.2(4)
C9–N10–C11–C12	173.6(8)	175.4(8)	176.0(3)	175.2(5)	175.8(4)
N10–C11–C12–N13	−22.1(1.0)	−25.4(1.1)	31.0(5)	21.8(8)	36.0(5)
C11–C12–N13–C14	167.5(7)	168.7(8)	−165.7(3)	−163.0(5)	−169.2(3)
C12–N13–C14–C15	−172.0(7)	−170.9(8)	82.6(4)	82.7(6)	82.9(5)
N13–C14–C15–N16	63.6(1.0)	61.6(1.2)$^{¥}$	57.4(4)	57.0(7)	57.7(7)
C14–C15–N16–C17	82.0(9)	78.3(1.2)$^{¥}$	83.6(4)	82.3(6)	82.9(5)
C15–N16–C17–C18	−164.4(7)	−160.8(1.0)$^{¥}$	−166.1(3)	−166.7(5)	−169.2(3)
N16–C17–C18–N1	36.7(1.2)	40.5(1.5)$^{¥}$	31.7(5)	30.7(8)	36.0(5)
C17–C18–N1–C2	168.5(8)	172.4(1.0)	174.3(4)	178.1(5)	175.8(4)

$^{@}$ N(py) – nitrogen atom of pyridine unit, N(am) – nitrogen atom of aliphatic amino group. $^{£}$ N_6-plane = the mean plane defined by positions of all six macrocycle nitrogen atoms (Shelxl MPLA command). $^{\$}$ The molecule has C_2-symmetry, the symmetry axis connects the centres of the CH_2–CH_2 bonds of the ethylenediamine fragments and passes through the central Pr^{3+} ion (corresponding transformation # = $-x + 1, y, -z + ½$); atoms corresponding to labels used in the Table: C6 = C5$^{\#}$, N7 = N4$^{\#}$, C8 = C3$^{\#}$, C9 = C2$^{\#}$, N10 = N1$^{\#}$, C11 = C9$^{\#}$, C12 = C8$^{\#}$, N13 = N7$^{\#}$, C14 = C6$^{\#}$, C15 = C6, N16 = N7, C17 = C8, C18 = C9. $^{¥}$ Calculated for the position of disordered atom N16 with higher occupancy.

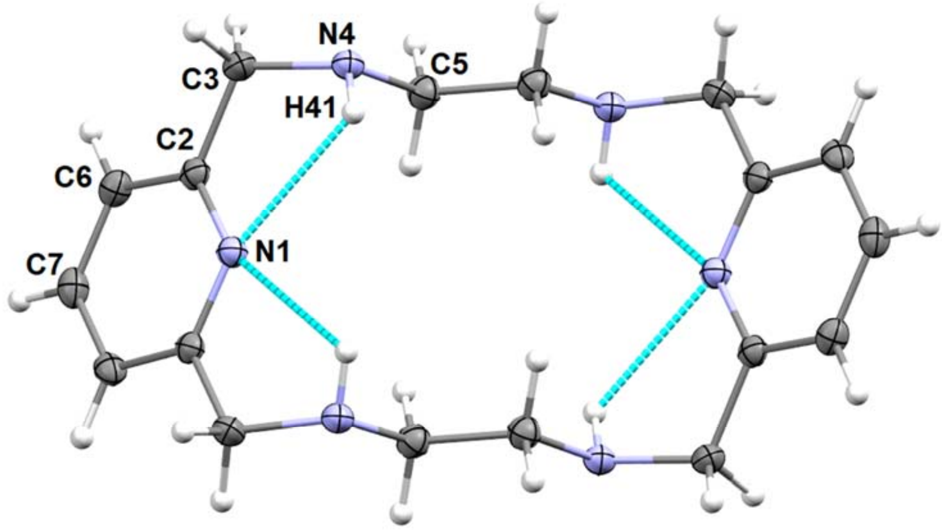

Figure S5. Molecular structure of 18-py$_2$N$_4$ found in the crystal structure of 18-py$_2$N$_4$·4H$_2$O. Only atoms belonging to the structurally independent quarter of the C_{2h}-symmetric molecule are labelled. Intramolecular hydrogen bonds are shown in turquoise.

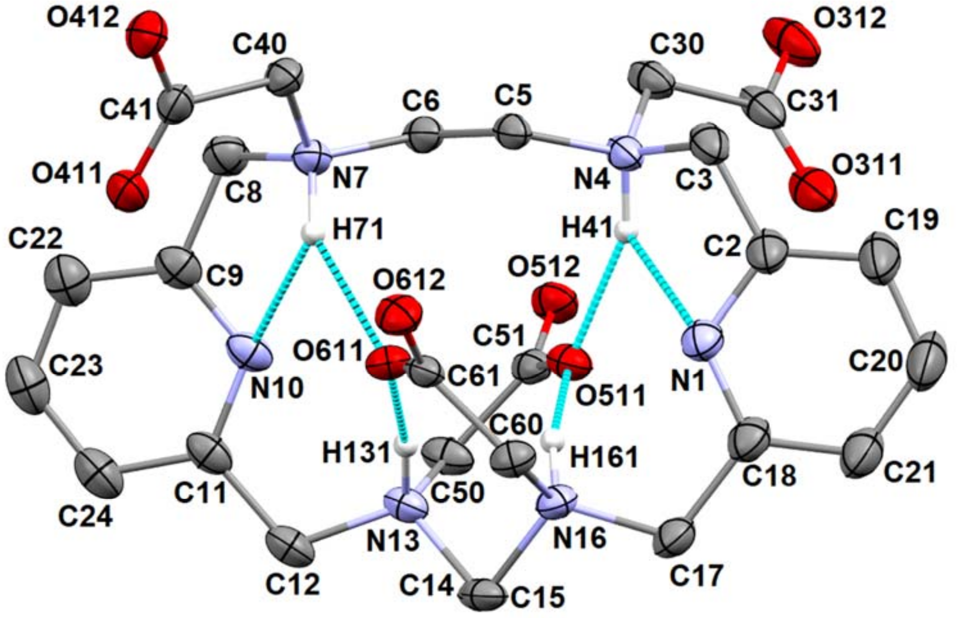

Figure S6. Molecular structure of one of the independent molecules of H$_4$pyta found in the crystal structure of H$_4$pyta·8H$_2$O. Carbon-bound hydrogen atoms are omitted for the sake of clarity. Intramolecular hydrogen bonds are shown in turquoise.

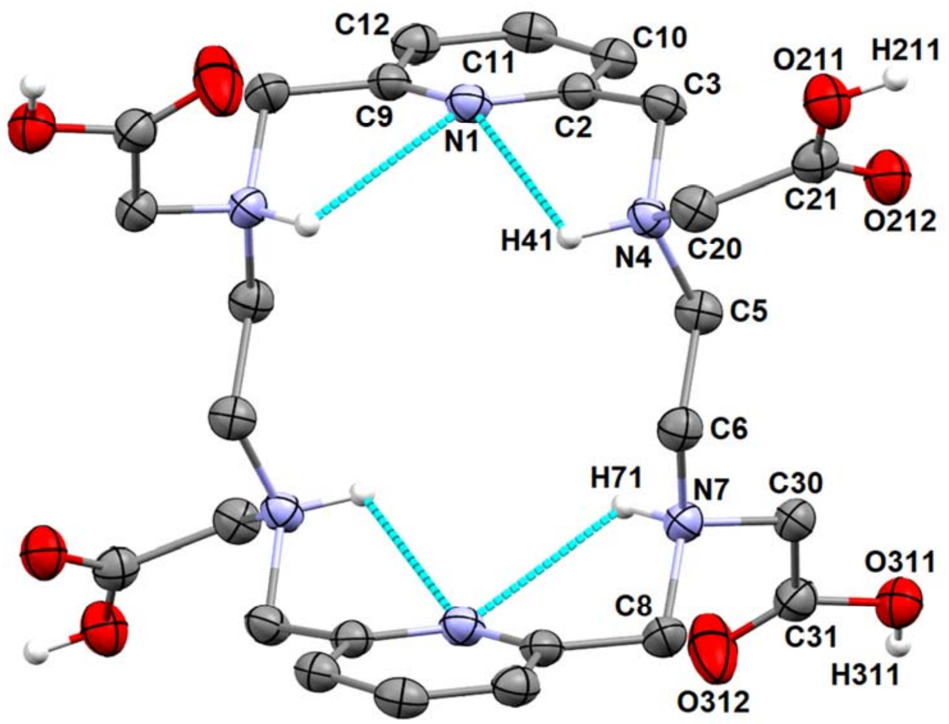

Figure S7. Molecular structure of $(H_8\mathbf{L})^{4+}$ cation found in the crystal structure of $(H_8\mathbf{L})Cl_4{\cdot}3H_2O$. Only atoms belonging to the structurally independent half of the centrosymmetric molecule are labelled. Carbon-bound hydrogen atoms are omitted for the sake of clarity. Intramolecular hydrogen bonds are shown in turquoise.

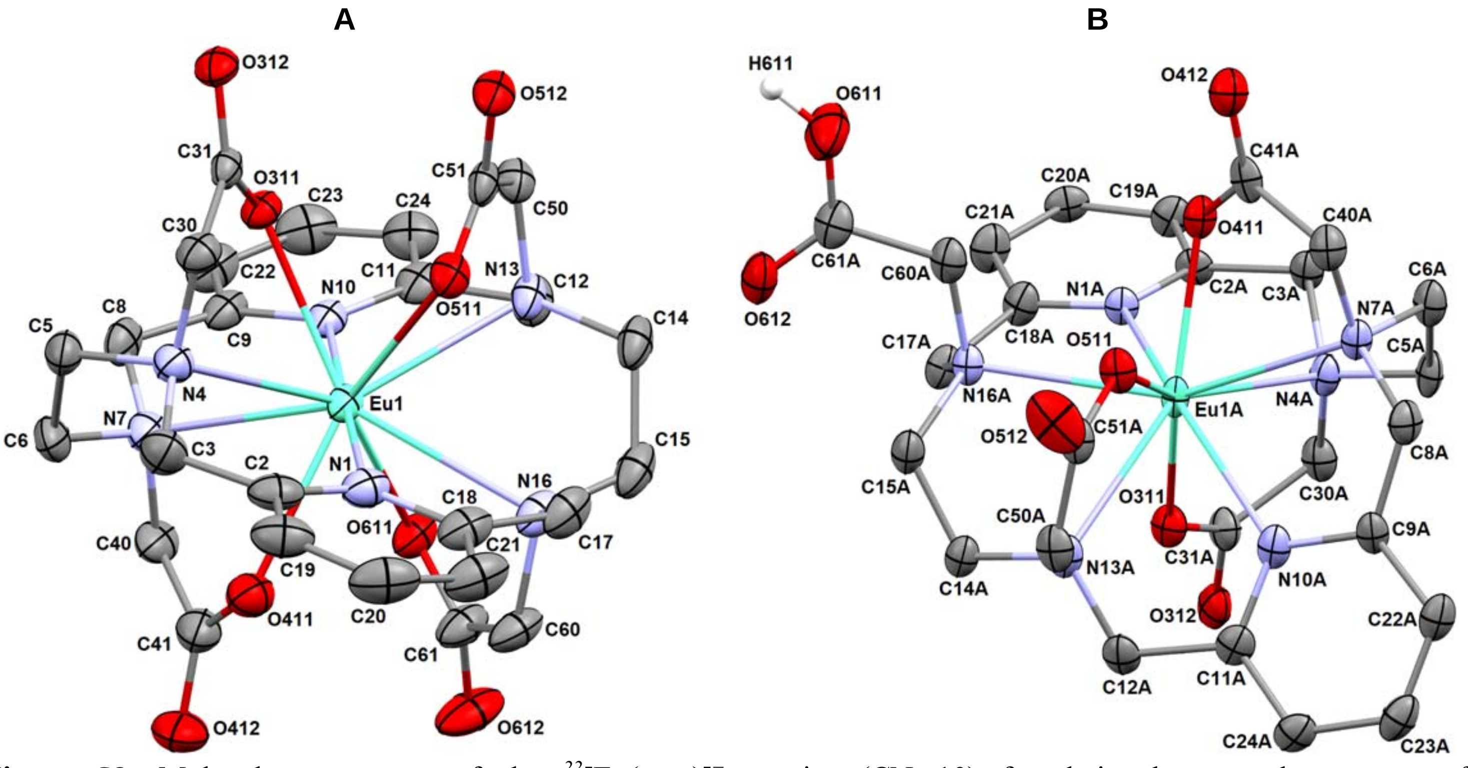

Figure S8. Molecular structures of the $^{22}[Eu(pyta)]^{-}$ species (CN 10) found in the crystal structure of $Na^{22}[Eu(pyta)]{\cdot}13.5H_2O$ (**A**) and one of two structurally independent $^{21}[Eu(Hpyta)]$ molecules (CN 9) present in the crystal structure of $^{21}[Eu(Hpyta)]{\cdot}3H_2O$ (**B**) with the atom numbering scheme. Carbon-bound hydrogen atoms are omitted for clarity.

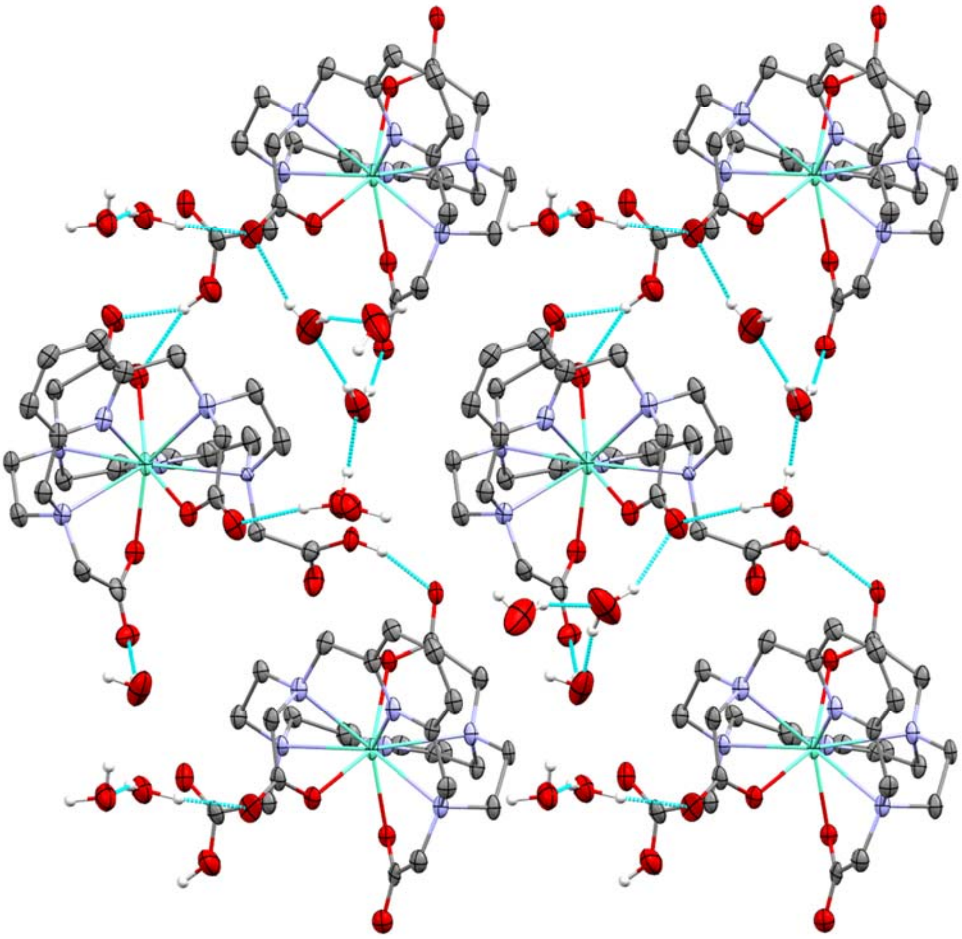

Figure S9. A part of the crystal packing found in the crystal structure of [21][Eu(Hpyta)]·3H_2O showing an intermolecular hydrogen bond system. Carbon-bound hydrogen atoms are omitted for clarity. Colour code: **Eu: light green**, **O: red**, **N: blue**, **C: dark grey**, **H: white**. Only positions of disordered atoms with higher occupancy are shown. Intermolecular hydrogen bonds are shown in turquoise.

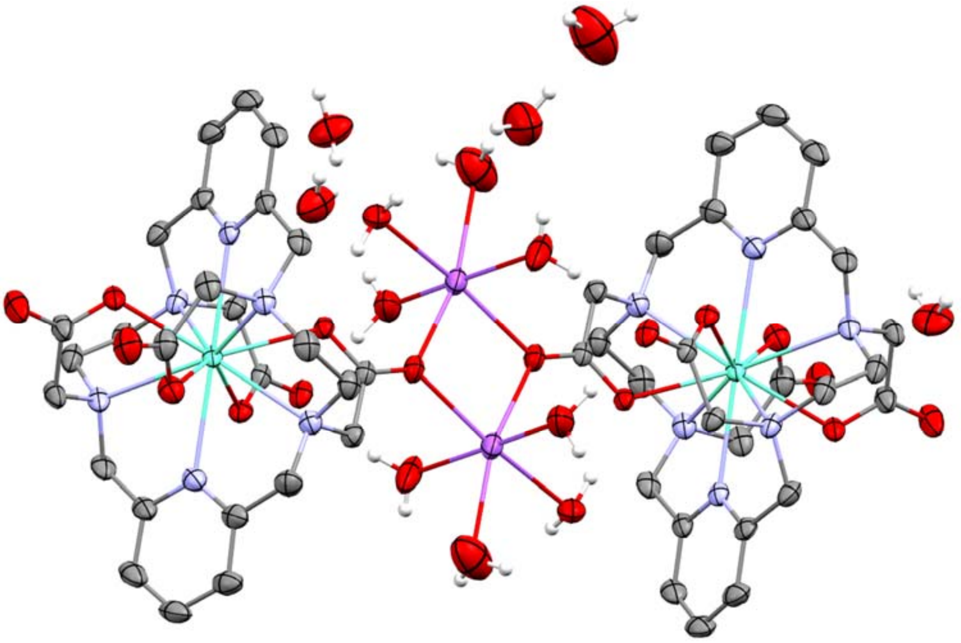

Figure S10. A part of the crystal packing found in the crystal structure of Na[22][Eu(pyta)]·13.5H_2O. Carbon-bound hydrogen atoms are omitted for clarity. Colour code: **Eu: light green**, **Na: purple**, **O: red**, **N: blue**, **C: dark grey**, **H: white**. Some disordered water molecules of crystallisation are not shown.

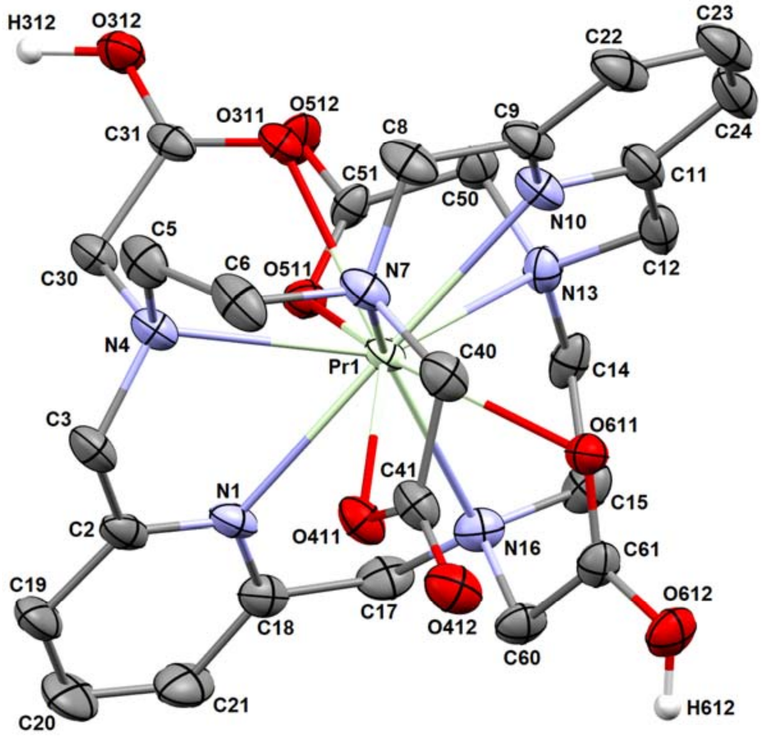

Figure S11. Molecular structure of the ^{22}[Pr(H_2pyta)]$^+$ cation found in the crystal structure of ^{22}[Pr(H_2pyta)]Cl·5H_2O. Carbon-bound hydrogen atoms are omitted for the sake of clarity.

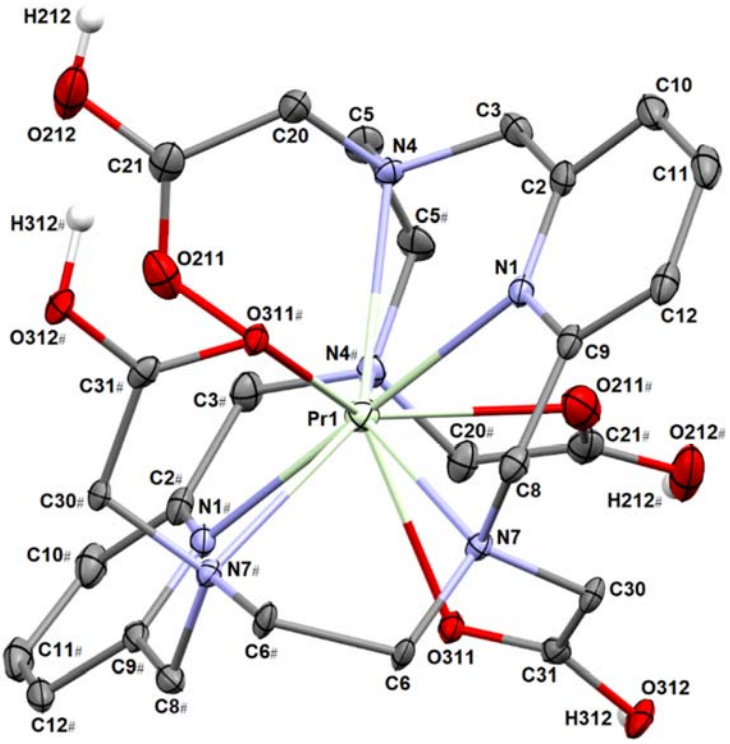

Figure S12. Molecular structure of the ^{22}[Pr(H_3pyta)]$^{2+}$ cation found in the crystal structure of ^{22}[Pr(H_3pyta)]Cl_2·3H_2O. Carbon-bound hydrogen atoms are omitted for the sake of clarity. The C_2-symmetry-related atoms are labelled by "#", the axis passes through the centres of the C5–C5# and C6–C6# bonds. The hydrogen atom H312 is half-occupied due to symmetry reasons.

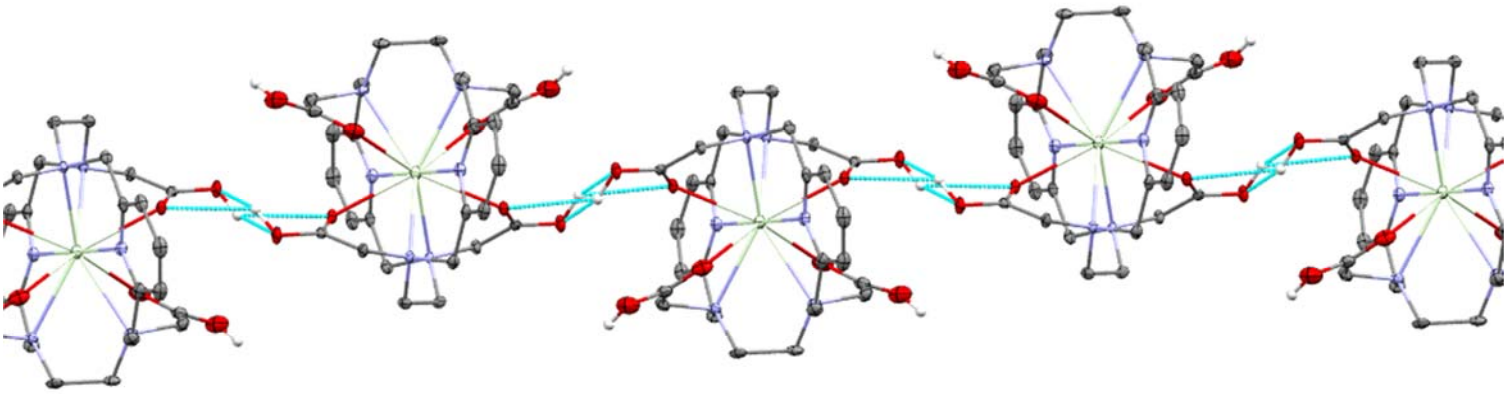

Figure S13. A part of the hydrogen-bonded chain {^{22}[Pr(H_3pyta)]$^{2+}$}$_n$ found in the crystal structure of ^{22}[Pr(H_3pyta)]Cl_2·3H_2O. Carbon-bound hydrogen atoms are omitted for the sake of clarity. Intermolecular hydrogen bonds are shown in turquoise; the corresponding hydrogen atom is split over two close centrosymmetry-related positions, each belonging to one of the neighbouring complex molecules. Colour code: **Pr: light green**, **O: red**, **N: blue**, **C: dark grey**, **H: white**.

Together with crystals of [22]$[Pr(H_3pyta)]Cl_2·3H_2O$, other crystals of different shapes were observed (crystallization at pH below ~0). The X-ray analysis revealed composition $[\{Pr(H_2O)_5\}_2(H_4pyta)]Cl_6·9H_2O$ where Pr(III) ions are located out of the ligand cage; the material is probably a product of a slow decomplexation at such low pH during a long crystallization time. The Pr(III) ions are coordinated by three carboxylate groups of three different ligand molecules – two carboxylate pendants are coordinated by one oxygen atom and the last one is coordinated in κ-*O*,*O′* fashion. No nitrogen atom is coordinated. All aliphatic amino groups of the macrocycle are protonated but the pyridine moieties are not protonated, consistently with the protonation scheme proposed for the free ligand (see above). Thus, the compound can be considered an *out-of-cage* 2D-coordination polymer. The coordination sphere of Pr(III) is closed by five water molecules to reach CN 9. Part of the solid-state structure is shown in Figure S14.

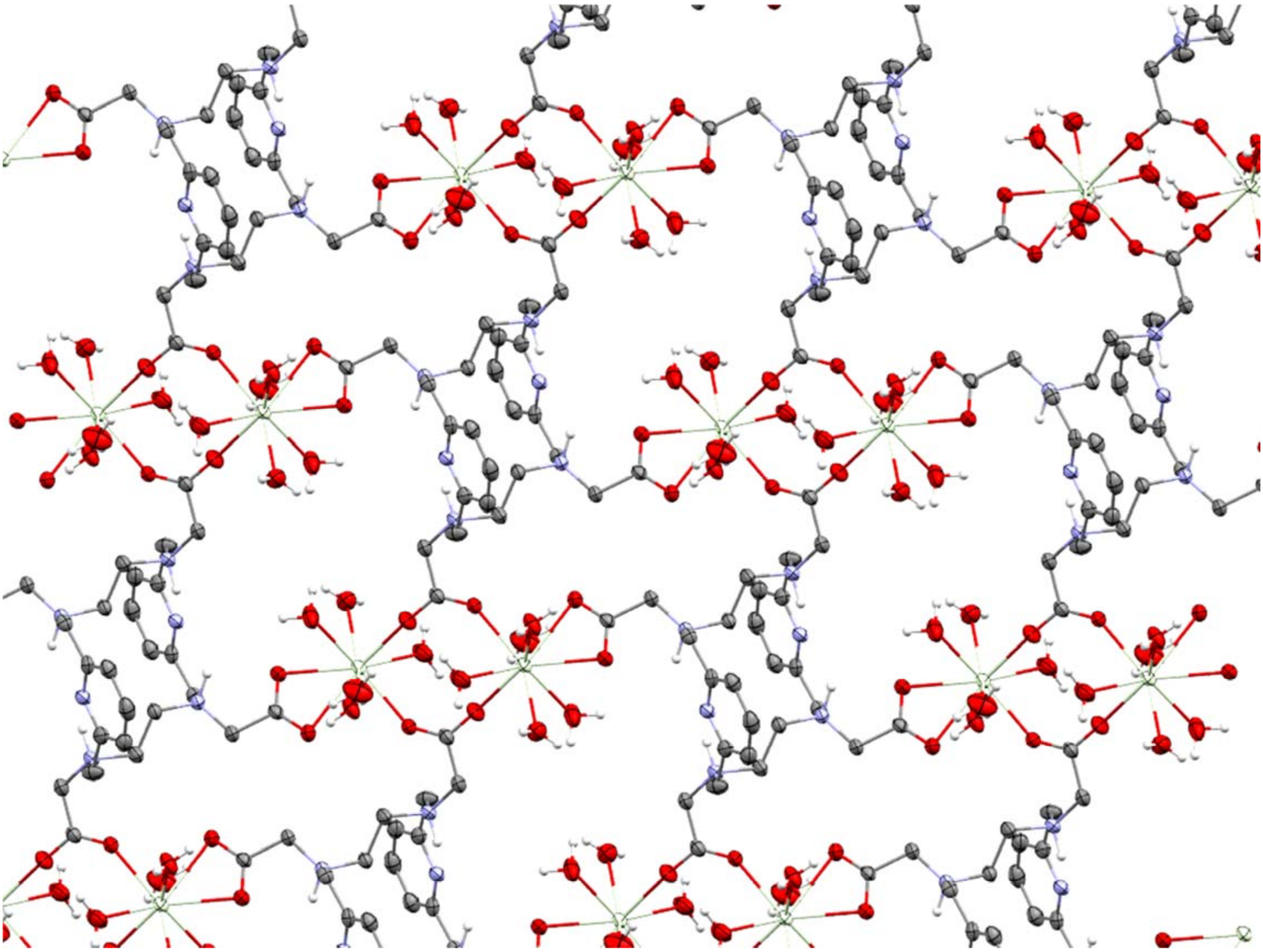

Figure S14. A part of the 2D-polymeric structure found in the crystal structure of $[\{Pr(H_2O)_5\}_2(H_4pyta)]Cl_6·9H_2O$. Non-coordinated water molecules and carbon-bound hydrogen atoms are omitted for the sake of clarity. Colour code: **Pr: light green**, **O: red**, **N: blue**, **C: dark grey**, **H: white**.

Equilibrium studies

Determination of protonation and stability constants

Potentiometry (0.1 M $(NMe_4)Cl$, 25.0 °C, pK_w 13.81) was performed according to the previously published procedures; further details on the preparation of stock solutions and chemicals, equipment, electrode system calibration, titration procedures and data treatment are described in refs.[8,9]. Calibration titrations of strong acid (HCl) with a strong base ($[NMe_4]OH$) were run before each titration of ligand or metal-ion-ligand systems to get calibration–titration pairs used in the data fitting. Protonation constants of H_4pyta were determined with c_L = 0.004 M, starting volume 5 mL, stream of Ar pre-saturated with water vapour, pH ranges 1.4–11.7 and 1.9–12.1, four parallel titrations in each range, 70 points per titration, calibration titrations in the same pH ranges. Full Ln(III) complexation was a slow process and, therefore, stability constants of Ln(III)–H_4pyta complexes were obtained by the "out-of-cell" ("batch") titrations as described previously:[10] starting volume 1 mL, each 1-mL solution is one titration point, $c_L \sim c_{Ln}$ = 0.004 M, 0.97 equiv. of Ln(III), pH range 1.6–4.5, two parallel titrations, ~28 points per titration, equilibration time 10 d. Times necessary to reach the equilibrium were determined by HPLC in independent experiments. The titration data were treated using OPIUM program.[11] Full sets of the determined overall constants $\log\beta_{hml}$ (with their standard deviations given directly by the program) are shown in Tables S10 and S11. Stability constants of Ln(III)-hydroxido complexes were retrieved from the literature.[12] The equilibrium constants are concentration constants. Throughout the paper, pH means $-\log[H^+]$. Figures S15 and S19 show distribution diagrams of the ligand and its complexes, respectively.

The NMR titration of the ligand to estimate protonation scheme (Figure S16) was performed at a ligand concentration of 0.03 M in water. The pH was adjusted by the addition of aq. LiOH or aq. HCl. The pH was determined using a calibrated combined electrode. Ionic strength was not controlled. The NMR data were collected with pre-saturation of the water signal, and with an insert tube containing D_2O (with 0.1% *t*BuOH) and NMR standard at 25 °C. The UV-Vis titration (Figure S18) was carried out in a pH range with a ligand concentration of 100 µM and pH was adjusted by the addition of aq. LiOH or aq. HCl. The pH was determined using a calibrated combined electrode. Ionic strength was not controlled. Spectra were recorded on a Specord 50 Plus system (Analytic Jena) in the range 220–300 nm.

The presence of the *22*/*21* isomers in the equilibrated solutions/titration points was determined by analytical HPLC. For Eu(III), Tb(III) and Lu(III), the minor isomer was present in the "titration points" with maximal abundance ~5% and, thus, the measured stability constants can be considered as stability constants of the *22* (Eu, Tb) and *21* (Lu) isomers of the complexes. "Titration points" of the Er(III)–H_4pyta system contained various amounts of each isomer in the solutions (Figure S20) and the equilibrium "constants" cannot be considered as correct ones for any isomer.

Table S10. Overall protonation, $\beta_{\mathrm{H}h\mathrm{L}}$, and consecutive protonation, $\log K_a$, constants of H_4pyta (25 °C, I = 0.1 M $(NMe_4)Cl$); errors in parenthesis are given directly by the fitting code. Comparison of $\log K_a$ of other ligands (25 °C).

h in	$\beta_{\mathrm{H}h\mathrm{L}}$	$\log K_a$					
$\beta_{\mathrm{H}h\mathrm{L}}$	H_4pyta	H_4pyta		H_4dota[b]	H_2macropa[c]	H_2py-macromonopa[d]	H_2py_2-macrodipa[e]
1	9.369(9)	9.37	9.37[a]	12.9	7.41	7.20	7.58
2	18.141(6)	8.77	8.81[a]	9.72	6.85	6.54	6.48
3	23.808(9)	5.67	5.80[a]	4.62	3.32	3.17	3.52
4	28.38(1)	4.57	4.71[a]	4.15	2.36	2.31	2.60
5	31.12(1)	2.74		2.29	1.69	1.69	2.10
6	32.88(1)	1.76		1.34			
7	33.92(3)	1.05					
Σ2N[f]		18.14	18.18[a]	22.6	14.26	13.74	14.06

[a] Ref.13 (I = 0.1 M KCl). [b] Ref.14 (I = 0.1 M $(NMe_4)Cl$). [c] Ref.15 (I = 0.1 M KCl). [d] Ref.16 (I = 0.1 M KCl). [e] Ref.17 (I = 0.1 M KCl). [f] Σ2N is "overall" basicity of the ring amino groups ($\log K_1 + \log K_2$).

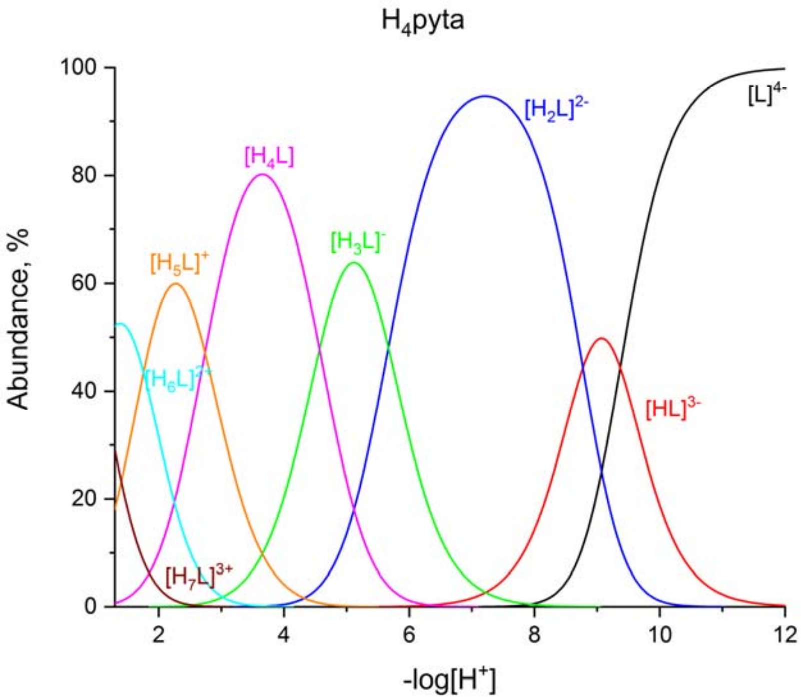

Figure S15. Distribution diagram of H_4pyta.

Ligand protonation scheme

According to the ^{1}H NMR titration curve (Figure S16), the protonation scheme shown in Figure S17 can be suggested. The first two protons are attached to the ethylene-diamine nitrogen atoms, as documented by a large change of the ^{1}H NMR shift of the ethylene hydrogen atoms. The double-protonated species is probably well stabilised by intramolecular hydrogen bonds within the ethylene-diamine fragments and with carboxylate pendant arms, similar to double-protonated species of tetraazamacrocycles, cyclen or cyclam derivatives. The abundance of such stabilised species in a neutral solution reaches ~100 % (Figure S15). Next two protons are bound to the other two amino groups of the ethylene-diamine fragments. It is reflected by a similar large change in the ^{1}H NMR shift of the ethylene

hydrogen atoms to that observed for the first two protonations. It leads to a rearrangement of the intramolecular hydrogen bonds and the pyridine nitrogen atoms are probably involved as acceptors in newly formed hydrogen bonds (chemical shifts of the pyridine hydrogen atoms as well as of the lutidine-like methylene groups are also affected). The same protonation was found in the solid-state structure of the zwitterionic form of the ligand (H_4pyta·$4H_2O$; see above, Figure S6). Next protonations occur dominantly on the carboxylate oxygen atoms, leading to a re-distribution of the hydrogen bonds as one/two/three carboxylic group(s) are not involved in the hydrogen-bond system (chemical shifts of the ethylene-diamine fragment and acetates are influenced). Direct protonation of the pyridine nitrogen atoms probably does not occur even in strong acidic solutions. It is consistent with the protonation of $(H_8\text{pyta})^{4+}$ cation found in the solid-state structure of $(H_8\text{pyta})Cl_4·3H_2O$ where four ethylene-diamine amino groups and oxygen atoms of four carboxylic groups are protonated (see above, Figure S6).

The re-arrangements of the hydrogen bond system during successive protonation of the ligand connected with higher/smaller involvement of the pyridine aromatic fragment is also supported by changes in UV absorption of the pyridine fragment (Figure S18).

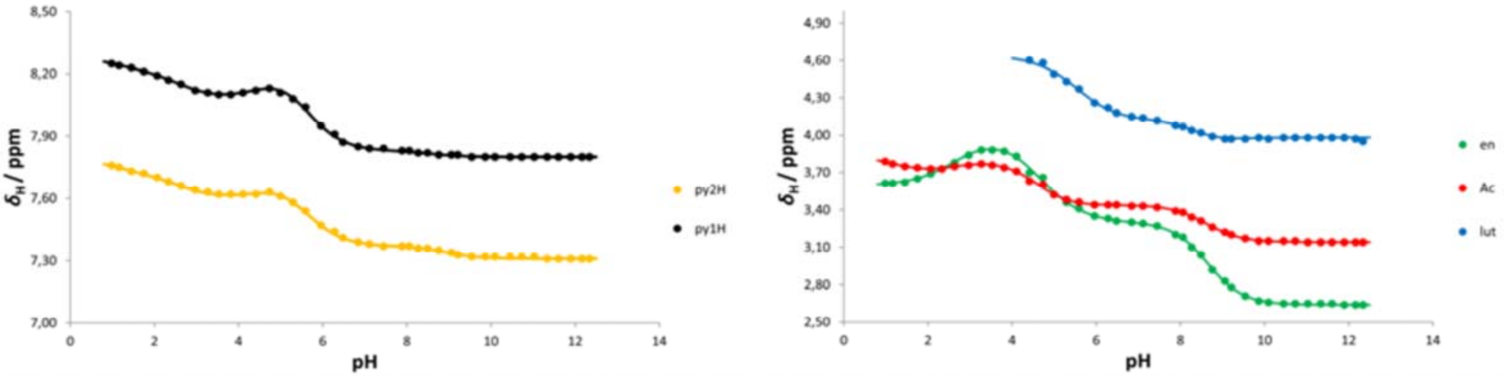

Figure S16. The ^{1}H NMR titration of H_4pyta (~10 mM ligand in H_2O, D_2O insert, water signal pre-saturation, pH adjusted by aq. HCl or aq. LiOH, 25 °C, no control of ionic strength, electrode system calibrated by standard buffer solutions).

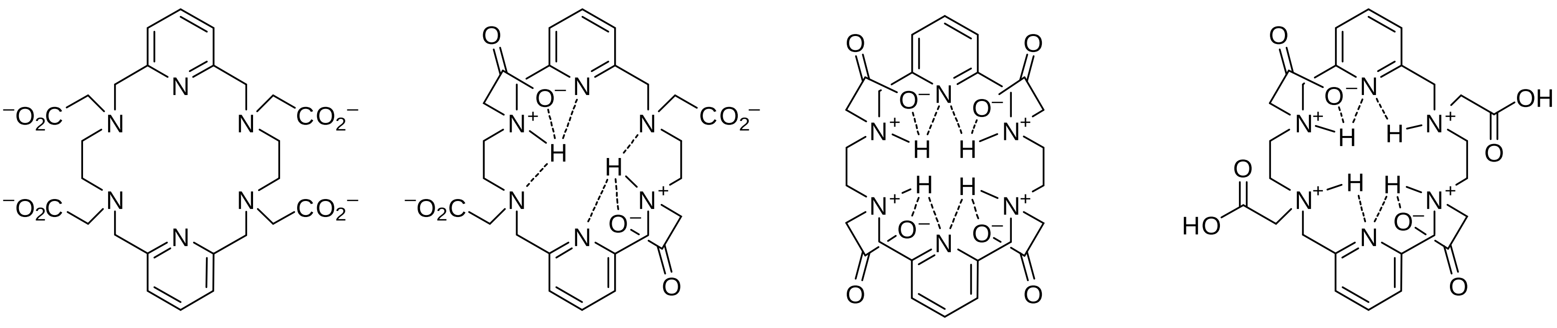

pyta^{4-} H$_2$pyta^{2-} H$_4$pyta H$_6$pyta^{2+}

Figure S17. Suggested protonation scheme of H_4pyta.

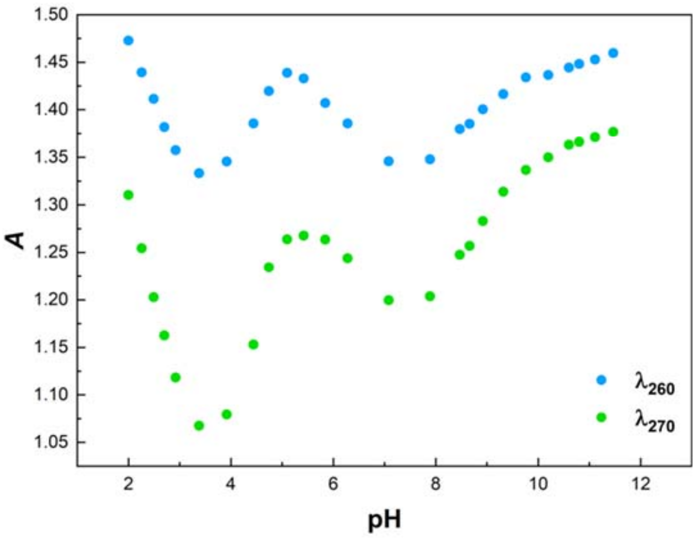

Figure S18. The UV titration of H_4pyta in water followed on absorption bands of the pyridine rings at 260 / 270 nm (100 μM ligand in H_2O, pH adjusted by diluted aq. HCl or aq. LiOH, 25 °C, no control of ionic strength, electrode system calibrated by standard buffer solutions).

Table S11. Overall, $\beta_{\mathrm{H}h\mathrm{ML}}$ (h = 0,1), and derived, $\log K_\mathrm{a}$ (*in italics*), stability constants of Ln(III)–H_4pyta complexes (25 °C, 0.1 M $(NMe_4)Cl$); errors in parenthesis are given directly by the fitting code.

Constant	Ln(III)						
	La	Ce	Nd	Eu	Tb	Er	Lu
β_{ML}	24.78(2)	25.67(3)	25.87(3)	26.23(2)	25.60(3)	"24.13(5)" [a]	23.15(6)
$\log K_{\mathrm{ML}}$	24.78	25.67	25.87	26.23	25.60	"24.13"	23.15
β_{HML}	26.49(6)	27.84(4)	28.58(2)	28.40(3)	28.44(2)	"27.50(2)" [a]	27.27(1)
$\log K_{\mathrm{HML}}$	*1.71*	*2.18*	*2.70*	*2.17*	*2.84*	*"3.37"*	*4.13*

[a] In the equilibrated solutions, different amounts of the *22* and *21* isomers were present at various pH (HPLC, Figure S20).

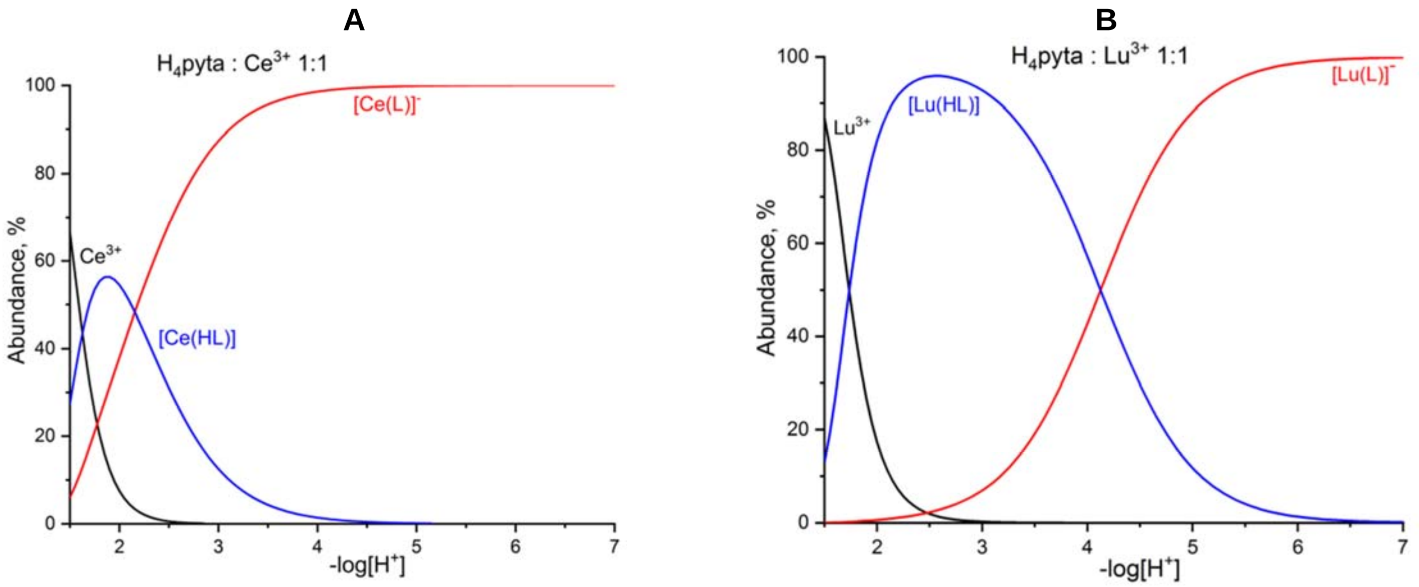

Figure S19. Distribution diagrams of Ce(III) (*22* isomer) (**A**) and Lu(III) (*21* isomer) (**B)** complexes of H_4pyta.

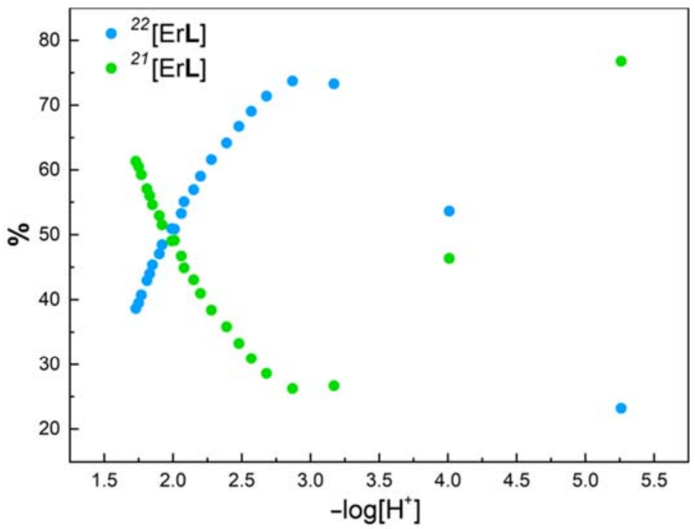

Figure S20. Abundance of the *22* and *21* isomers in the Er(III)–H_4pyta system in the equilibrated solutions at different pH during the "out-of-cell" potentiometric titration, as determined by HPLC.

Protonation constants of the pre-formed complexes

The pre-formed Ce(III) and Lu(III) complexes in solution were obtained by mixing a known amount of the ligand stock solution (~5% molar excess) with a known amount of $LnCl_3$ stock solutions in a glass ampule and a slow portion-wise addition (~30 min) of known amount of standard $(NMe_4)OH$ solution (~3.8 equiv., near to full neutralisation of the ligand amount, pH ~6) under Ar. The ampules were flame-sealed and left at room temperature for 7 days. To ensure the full complexation of the metal ions, the sealed ampules were finally heated at 90 °C overnight. The ampules were opened, the presence of the single isomer (100% *22* isomer for Ce(III) and >97% *21* isomer for Lu(III)) was confirmed by HPLC and aliquots of the solutions of the Ln(III) complexes were transferred into a titration vessel. Water and standardized HCl and $(NMe_4)Cl$ solutions were added (to reach a pH ~1.4 and I = 0.1 M $(H,NMe_4)Cl$ in the final solution, starting volume 5 mL, complex concentration ~0.003 M). These solutions were immediately titrated with a standardized $(NMe_4)OH$ solution up to pH ~7 (until the appearance of a light precipitate) at 25.0 °C. Three titrations were performed, each consisting of around 40 data points. The titration data were treated with the OPIUM[11] program. The electrode system was calibrated by strong acid-strong base calibration titration in the pH range of 1.4–11.7. Due to the kinetic inertness of the pre-formed complexes, they do not decompose in the acid solutions during the time scale of the titration (~20 min in the acidic solutions). The determined overall/stepwise protonation constants of the complexes are given below.

Protonation constants of the pre-formed [Ln(pyta)]$^-$ complexes (I = 0.1 M $(H,NMe_4)Cl$, 25 °C).

Ce(III): logβ_1 2.03(1) and logβ_2 3.769(8) *i.e.* logK_1 2.03 and logK_2 1.74.

Lu(III): logβ_1 3.60(1) and logβ_2 5.16(1) *i.e.* logK_1 3.60 and logK_2 1.56.

Kinetic measurements

UV-vis spectrophotometry

The measured samples had molar concentration of the complexes ~0.1 mM. Dissociation kinetics was followed at temperatures of 25, 50, 60, 70, 80 or 90 ± 0.1 °C in the aq. $HClO_4$ or HCl media of different acidities (c_{H+} = 0.5–5.0 M, I = 5.0 M (H,Na)ClO_4 or (H,Na)Cl). Into a quartz cuvette with a Teflon stopper, distilled water (143 µl), a stock aq. $NaClO_4$ or NaCl (5.905 M; appropriate volume to finally achieve a total volume of 0.990 ml after adding the stock aq. $HClO_4$ or HCl) and a calculated amount of the stock aq. $HClO_4$ or HCl (5.905 M) were pipetted. This solution was pre-heated to the desired temperature for at least 3 min. Subsequently, an aqueous solution of the $[Ln(pyta)]^-$ (0.01 M, 10 µl; prepared from pure isomers or mixture of the isomers) was added. The reaction mixture was quickly and thoroughly shaken, and, after reaching the temperature of the experiment, UV-vis spectra were measured at time intervals, or change in absorbance at a given wavelength was monitored, for at least three half-lives. Dead time in the individual experiments was short enough and can be neglected.

Acid-assisted decomposition of the Ce(III)–H_4dota (LMCT band at 266 nm) and La(III)/Ce(III)–H_2macropa (pyridine absorption band at 276 nm) complexes was also investigated spectrophotometrically. The complex concentration in the sample solution was ~1.0 mM. Measured solutions of the complexes were prepared using the analogous procedure as described above for the H_4pyta complexes.

The observed rate dissociation constants (${}^{d}k_{obs}$) were obtained by fitting (least-squares method) experimentally measured absorbances at various time points according to Equation S1 where ${}^{d}k_{obs}$ represents the observed rate constant of the decomplexation reaction and A_t, A_0 and A_1 are absorbances at time t, and at the beginning and the end of the reaction, respectively.

$$A_t = A_1 + (A_0 - A_1) \cdot e^{{}^{d}k_{obs} \cdot t} \qquad \text{(S1)}$$

In the case of extended measurements (hours), absorption UV-vis spectra were recorded at suitable time intervals. For short-time measurements, the change in absorbance was monitored at a single wavelength. Individual time changes in absorbance were evaluated at wavelengths 260, 266, and/or 276 (where the largest change in absorbance occurred).
It was also possible to perform kinetic measurements directly on a mixture of both *22*/*21* isomers. Initially, under milder conditions (50 °C), dissociation of the *21* isomer occurs while the amount of the *22* isomer in the mixture remains almost unchanged. The results of the dissociation measurements of these isomers in their mixture were consistent with the results of the dissociation measurements of the pure isomers. For the sake of correctness, results from the decomplexation of the pure isomers are always reported.

To get information about trends along whole lanthanide series, the decomposition of the Y(III) and all Ln(III) complexes was followed in 5.0 M $HClO_4$ at 90 °C. Examples of the spectrophotometric kinetic data are shown in Figures S21 and S22 and the results are presented in Table S12. However for the smaller Ln(III) complexes and UV-Vis measurements, intermittent checks of the studied solutions by HPLC showed that the decomplexation is accompanied by isomerisation of the complexes. The isomerisation was significantly suppressed at 50 °C.

Decomplexation rates for selected Ln(III)–H_4pyta complexes and the $[Ce(dota)]^-$ complex at different temperatures are presented in Table S14 with examples of the data in Figure S25. Decomplexation rates for selected Ln(III)–H_4pyta complexes at different solution acidities are given in Table S15 with examples of the data in Figure S26.

HPLC

To validate the UV-Vis decomplexation experiments, the decomplexation of selected complexes was also followed by HPLC. As the method allows the separation and quantification of both *22* and *21* isomers, it can be also used for the mixture of the isomers obtained directly from the complex synthesis. To follow the course of decomplexation reactions by HPLC-UV/Vis-MS, the experiments were run under identical conditions as those with UV-vis measurements. The samples of the solutions for decomplexations were prepared as above (final volume 1 mL) in vials which were placed into an oil bath (50 and 90 °C). Aliquots were taken from the reaction mixture (containing one or both isomers) at appropriate time intervals by hand and were analysed as quickly as possible by HPLC-UV/Vis-MS (C18-AQ silica gel, column 4.6×50 mm, particle size 2.7 μm, mobile phase 0.1% (v/v) aq. TFA:ACN, method G3). The identification of individual reaction components/products was carried out using mass spectrometry. Amounts of the free ligand and the individual isomers were determined by integrating the peak areas in chromatograms (absorbance at a wavelength of 266 nm). The data were evaluated according to Equations S2 and S3 (AUC_t, AUC_0 and AUC_1 are areas under curve at time t, and at the beginning and the end of the reaction, respectively), examples of the experimental data are shown in Figure S23.

$$AUC_t = AUC_1 + (AUC_0 - AUC_1) \cdot e^{\,{}^{d}k_{obs} \cdot t} \qquad \text{(S2)}$$

$$\text{Normalisation: } AUC_{t;\,\text{free H4L}} + AUC_{t;\,220} + AUC_{t;\,211} = 100\ \% \qquad \text{(S3)}$$

The data obtained by spectrophotometry and HPLC agreed within the experimental errors (Figure S24, Table S13). Dead time for the first sample to be manually injected into the HPLC system was about 2 min.

Evaluation of the decomplexation kinetic data

The kinetic inertness of complexes with macrocyclic ligands is mostly assessed through decomposition of these complexes in strongly acidic environments where, thermodynamically, these complexes cannot exist. This decomposition is usually acid-catalysed and involves several steps, including protonation of the ligand in the complexes. The mechanism typically consists of at least two pathways.[18] The first one is decomposition of a species without proton involvement (*i.e.* the less protonated species). The other pathway is decomposition of a species being more protonated, Equations S4 and S5 (charges of the complexes are omitted).

$$[\text{Ln}(\text{H}_x\textbf{L})] \xrightarrow{{}^{d}k_0} \text{Ln}^{3+} + \text{H}_x\textbf{L} \qquad \text{(S4)}$$

$$[\text{Ln}(\text{H}_x\textbf{L})] \underset{\text{H}^+}{\overset{K_\text{H}}{\rightleftharpoons}} [\text{Ln}(\text{H}_{x+1}\textbf{L})] \xrightarrow{{}^{d}k_1} \text{Ln}^{3+} + \text{H}_{x+1}\textbf{L} \qquad \text{(S5)}$$

In the Equations, ${}^{d}k_0$ represents a rate constant of spontaneous dissociation of the thermodynamically stable species and K_H is a protonation constant of the thermodynamically stable species. This protonation initiates proton-induced decomplexation with a rate constant ${}^{d}k_1$. This mechanism is described by Equation S6 where ${}^{d}k_{obs}$ is the observed rate constant of the complex decomposition.

$$^{d}k_{obs} = \frac{^{d}k_0 + {}^{d}k_1 \cdot K_H \cdot [H^+]}{1 + K_H \cdot [H^+]} = \frac{^{d}k_0 + {}^{d}k_H \cdot [H^+]}{1 + K_H \cdot [H^+]} \qquad \text{(S6)}$$

The term ${}^{d}k_1 \cdot K_H$ can be simplified into a rate constant ${}^{d}k_H = {}^{d}k_1 \cdot K_H$. Contribution of the term $K_H \cdot [H^+]$ in the denominator is very small if value of the protonation constant K_H is very low (i.e. protonation takes place in a very acidic solution) and, thus, it can be neglected relative to the unity. Since overall molar concentration of protons in the reaction is high and is determined by the analytical concentration of the used $HClO_4$ or HCl, Equation S6 can be simplified to form Equation S7.

$${}^{d}k_{obs} = {}^{d}k_0 + {}^{d}k_H \cdot c_{H+} \qquad \text{(S7)}$$

For decomplexation of the $[Ln(pyta)]^-$ complexes not disturbed by the isomerisation, a linear dependence of ${}^{d}k_{obs}$ on proton concentration was observed and, therefore, Equation S7 was used to evaluate the decomplexation data of the $[Ln(pyta)]^-$ complexes as a function of the concentration of the mineral acids.

Values of ${}^{d}k_0$ and ${}^{d}k_H$ and activation parameters of ${}^{d}k_{obs}$ for the selected Ln(III)–H_4pyta complexes and the $[Ce(dota)]^-$ complex are listed in Tables S14 and S15. Decomplexation data for the $[Ce(dota)]^-$ and $[La(macropa)]^+$ complexes at various solution acidities are given in Table S16.

Table S12. Observed decomplexation rate constants ${}^{d}k_{obs}$ and decomplexation half-lives ${}^{d}\tau_{½}$ of Ln(III)–H_4pyta complexes (5.0 M $HClO_4$, 90 °C, UV-Vis). The individual ${}^{d}k_{obs}$ are values obtained from three independent measurements with different batches of stock solutions. The decomplexation half-life ${}^{d}\tau_{½}$ is an average of three corresponding values calculated from these three ${}^{d}k_{obs}$. Decomplexations disturbed by isomerisation between the *22* and *21* isomers (Dy→Lu and Y) are grey-highlighted.

Complex	${}^{d}k_{obs}$ {1st}, s^{-1}	${}^{d}k_{obs}$ {2nd}, s^{-1}	${}^{d}k_{obs}$ {3rd}, s^{-1}	${}^{d}\tau_{½}$, min
^{22}[La(pyta)]$^-$	$5.05(18)\cdot10^{-3}$	$5.32(3)\cdot10^{-3}$	$4.82(9)\cdot10^{-3}$	2.29
^{22}[Ce(pyta)]$^-$	$1.20(1)\cdot10^{-3}$	$1.18(2)\cdot10^{-3}$	$1.29(2)\cdot10^{-3}$	9.39
^{22}[Pr(pyta)]$^-$	$2.87(2)\cdot10^{-4}$	$2.97(6)\cdot10^{-4}$	$2.71(5)\cdot10^{-4}$	40.6
^{22}[Nd(pyta)]$^-$	$1.35(3)\cdot10^{-4}$	$1.40(2)\cdot10^{-4}$	$1.49(4)\cdot10^{-4}$	81.6
^{22}[Sm(pyta)]$^-$	$9.38(2)\cdot10^{-5}$	$9.66(1)\cdot10^{-5}$	$8.91(3)\cdot10^{-5}$	124
^{22}[Eu(pyta)]$^-$	$7.34(3)\cdot10^{-5}$	$7.54(2)\cdot10^{-5}$	$7.16(3)\cdot10^{-5}$	157
^{22}[Gd(pyta)]$^-$	$8.42(7)\cdot10^{-5}$	$8.27(6)\cdot10^{-5}$	$7.75(3)\cdot10^{-5}$	142
^{22}[Tb(pyta)]$^-$	$1.81(1)\cdot10^{-4}$	$1.85(1)\cdot10^{-4}$	$1.98(1)\cdot10^{-4}$	61.6
^{22}[Dy(pyta)]$^-$	$3.75(1)\cdot10^{-4}$	$3.71(1)\cdot10^{-4}$	$3.94(3)\cdot10^{-4}$	30.8
^{22}[Ho(pyta)]$^-$	$5.62(2)\cdot10^{-4}$	$5.37(1)\cdot10^{-3}$	$5.92(6)\cdot10^{-4}$	20.5
^{22}[Er(pyta)]$^-$	$1.27(4)\cdot10^{-3}$	$1.18(4)\cdot10^{-3}$	$1.13(1)\cdot10^{-3}$	10.2
^{22}[Tm(pyta)]$^-$	$4.31(6)\cdot10^{-3}$	$4.50(1)\cdot10^{-3}$	$4.64(2)\cdot10^{-3}$	2.58
^{21}[Ho(pyta)]$^-$	$2.93(10)\cdot10^{-2}$	$2.58(4)\cdot10^{-2}$	$2.51(6)\cdot10^{-2}$	0.44
^{21}[Er(pyta)]$^-$	$3.28(2)\cdot10^{-2}$	$3.20(2)\cdot10^{-2}$	$3.01(1)\cdot10^{-2}$	0.37
^{21}[Tm(pyta)]$^-$	$3.88(1)\cdot10^{-2}$	$3.77(2)\cdot10^{-2}$	$4.00(1)\cdot10^{-2}$	0.30
^{21}[Yb(pyta)]$^-$	$4.31(3)\cdot10^{-2}$	$4.25(4)\cdot10^{-2}$	$3.98(2)\cdot10^{-2}$	0.28
^{21}[Lu(pyta)]$^-$	$4.33(1)\cdot10^{-2}$	$4.54(6)\cdot10^{-2}$	$4.18(6)\cdot10^{-2}$	0.27
^{22}[Y(pyta)]$^-$	$7.78(5)\cdot10^{-4}$	$7.89(6)\cdot10^{-4}$	$7.72(2)\cdot10^{-4}$	14.8
^{21}[Y(pyta)]$^-$	$3.02(5)\cdot10^{-2}$	$3.04(2)\cdot10^{-2}$	$3.07(3)\cdot10^{-2}$	0.38

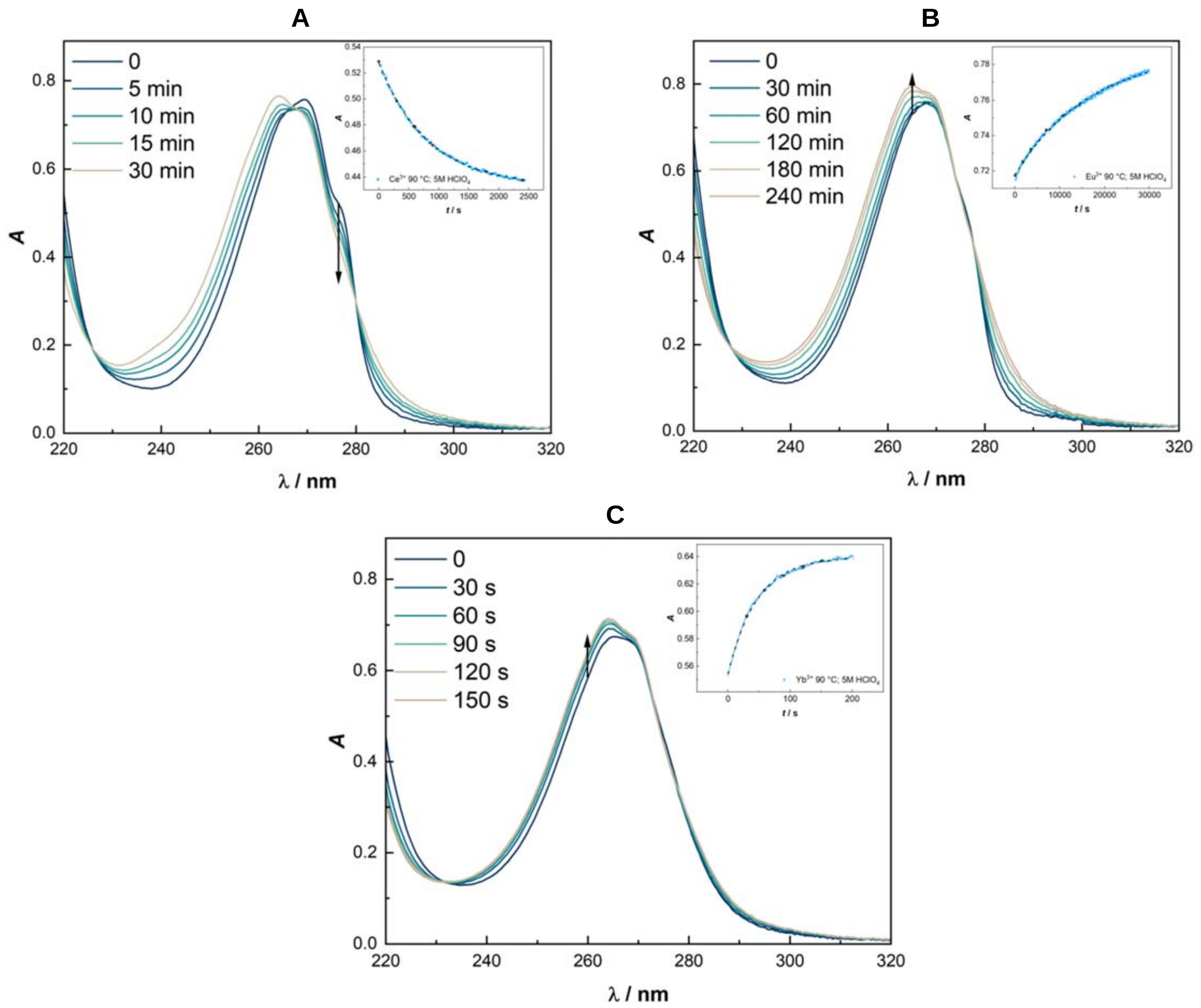

Figure S21. Examples of spectral changes in the course of the decomplexation reactions (5.0 M $HClO_4$, 90 °C): ^{22}[Ce(pyta)]$^{-}$ (**A**), ^{22}[Eu(pyta)]$^{-}$ (**B**) and ^{21}[Yb(pyta)]$^{-}$ (**C**) complexes. <u>Insets:</u> Time dependence of absorbance at a wavelength highlighted by the arrow.

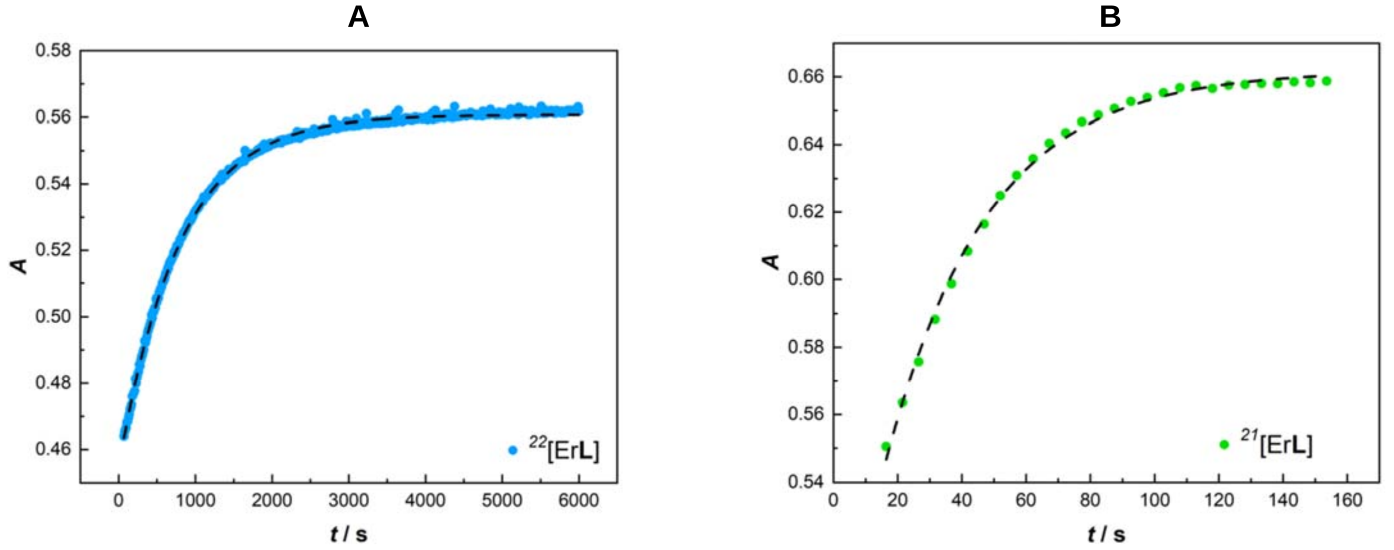

Figure S22. Examples of change in absorbance (at 260 nm) in the course of decomplexation reactions of ^{22}[Er(pyta)]$^{-}$ (**A**) and ^{21}[Er(pyta)]$^{-}$ (**B**) complexes (5.0 M $HClO_4$, 90 °C, measurement time interval 5 s).

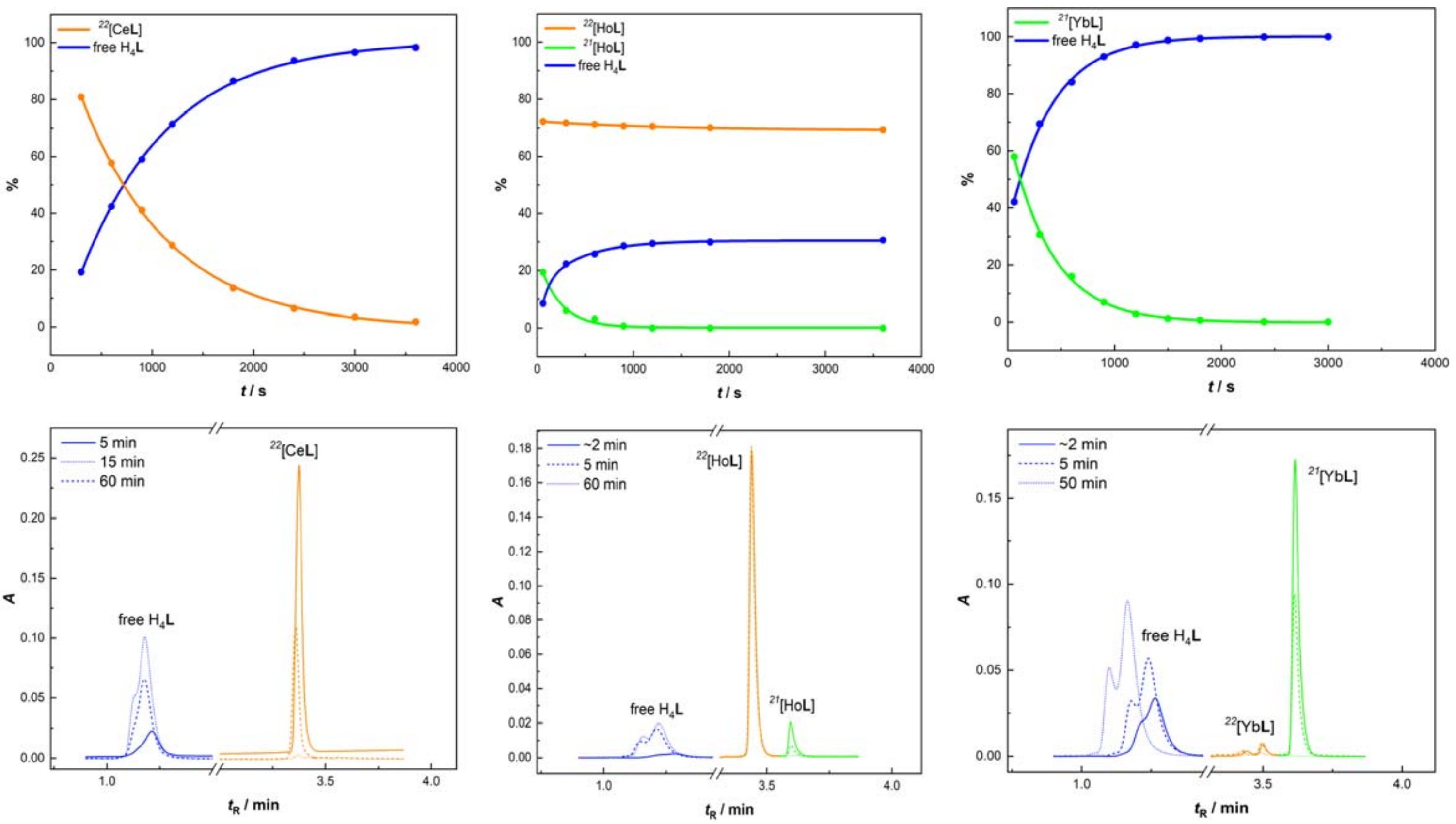

A B C

Figure S23. Changes of spectral intensities (top) and chromatograms (bottom) in the course of the decomplexation reaction of the complexes in 5.0 M $HClO_4$: **A**: ^{22}[Ce(pyta)]$^{-}$ at 90 °C; **B**: ^{22}[Ho(pyta)]$^{-}$ / ^{21}[Ho(pyta)]$^{-}$ at 50 °C and **C**: ^{21}[Yb(pyta)]$^{-}$ at 50 °C. Decomplexation of the Ho(III) and Yb(III) complexes were measured on a mixture of the *22* and *21* isomers obtained directly from the synthesis (a dead-time for HPLC check after start of the experiments is about 2–3 min).

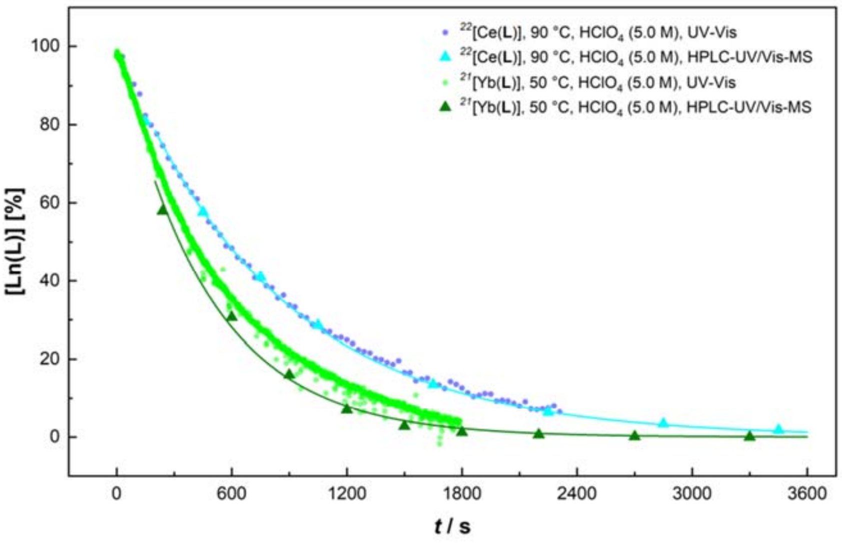

Figure S24. Experimentally determined abundances of the ^{22}[Ce(pyta)]$^{-}$ (**blue**, 90 °C) and ^{21}[Yb(pyta)]$^{-}$ (**green**, 50 °C) complexes in the course of the decomplexation reaction using UV-Vis spectroscopy (dots) and HPLC (triangles) (5.0 M $HClO_4$).

Table S13. Comparison of observed decomplexation rate constants ${}^{d}k_{obs}$ and half-lives $\tau_{½}$ of [Ln(pyta)]$^{-}$ complexes obtained by UV-Vis[a] and HPLC-UV/Vis-MS[b] in 5.0 M $HClO_4$ and at 50 or 90 °C.

	^{22}[Ce(pyta)]$^{-}$		^{21}[Yb(pyta)]$^{-}$	
Temperature	50 / *90* °C		50 °C	
Method	UV-Vis (276 nm)	HPLC-UV/Vis-MS	UV-Vis (260 nm)	HPLC-UV/Vis-MS
${}^{d}k_{obs}$ / s^{-1}	$0.03(1)\cdot10^{-3}$ / *$1.23(1)\cdot10^{-3}$*	$0.03(2)\cdot10^{-3}$ / *$1.21(2)\cdot10^{-3}$*	$2.73(1)\cdot10^{-3}$	$2.51(5)\cdot10^{-3}$
${}^{d}\tau_{½}$	~385 min = ~6.4 h / *9.4 min*	~385 min = ~6.4 h / *9.5 min*	4.2 min	4.6 min

	^{22}[Ho(pyta)]$^{-}$ [c,d]		^{21}[Ho(pyta)]$^{-}$ [d]	
Temperature	50 °C		50 °C	
Method	UV-Vis (260 nm)	HPLC-UV/Vis-MS	UV-Vis (276 nm)	HPLC-UV/Vis-MS
${}^{d}k_{obs}$ / s^{-1}	$0.02(2)\cdot10^{-3}$	$0.03(7)\cdot10^{-3}$	$2.11(5)\cdot10^{-3}$	$2.49(5)\cdot10^{-3}$
${}^{d}\tau_{½}$	~580 min = ~9.6 h	~385 min = ~6.4 h	5.5 min	4.6 min

[a]UV-vis measurements were carried out in a continuous mode. The observed dissociation constants ${}^{d}k_{obs}$ were obtained by fitting changes in absorbance as a function of time at the wavelength indicated in the parentheses. [b]The HPLC measurements were performed with manually-sampled aliquots. [c]Kinetic inertness of the ^{22}[Ho(pyta)]$^{-}$ complex at 90 °C (5.0 M $HClO_4$) expressed as ${}^{d}\tau_{½}$ was approx. 20 min (see Table S12); however, a partial isomerisation takes place. [d]Half-lives of the ^{22}[Er(pyta)]$^{-}$ and ^{21}[Er(pyta)]$^{-}$ complexes in 5.0 M $HClO_4$ and at 50 °C: ${}^{d}\tau_{½}$ = ~330 and 5.2 min, respectively (Table S15).

Table S14. Observed decomplexation rate constants ${}^{d}k_{obs}$ and corresponding conditional activation parameters of the selected Ln(III)–H_4pyta complexes and the $[Ce(H_2O)(dota)]^-$ complex at different temperatures (5.0 M $HClO_4$). Data at 25 °C were extrapolated from the temperature dependence. Data for the Er(III)– and Yb(III)–H_4pyta complexes (grey-highlighted) are distorted by the mutual *22*/*21* isomerisation (its extent varies with temperature).

T / K	^{22}[La(**L**)]$^-$	^{22}[Ce(**L**)]$^-$	^{22}[Eu(**L**)]$^-$	^{22}[Er(**L**)]$^-$	^{21}[Er(**L**)]$^-$	^{21}[Yb(**L**)]$^-$	[Ce(dota)]$^-$ [a]
363.15	$5.05(18)\cdot10^{-3}$	$1.23(1)\cdot10^{-3}$	$7.34(3)\cdot10^{-5}$	$1.13(1)\cdot10^{-3}$	$3.16(7)\cdot10^{-2}$	$4.18(1)\cdot10^{-2}$	$3.90(3)\cdot10^{-1}$
353.15	$2.31(4)\cdot10^{-3}$	$0.59(3)\cdot10^{-3}$	$4.35(2)\cdot10^{-5}$	$0.46(1)\cdot10^{-3}$	$1.73(4)\cdot10^{-2}$	$2.19(1)\cdot10^{-2}$	$2.20(1)\cdot10^{-1}$
343.15	$0.98(2)\cdot10^{-3}$	$0.20(1)\cdot10^{-3}$	$1.67(2)\cdot10^{-5}$	$0.20(2)\cdot10^{-3}$	$1.06(1)\cdot10^{-2}$	$1.17(4)\cdot10^{-2}$	$1.08(1)\cdot10^{-1}$
333.15	$0.42(1)\cdot10^{-3}$	$0.08(1)\cdot10^{-3}$	$1.00(7)\cdot10^{-5}$	$0.08(2)\cdot10^{-3}$	$4.71(1)\cdot10^{-3}$	$6.89(1)\cdot10^{-3}$	$7.61(4)\cdot10^{-2}$
323.15	$0.17(1)\cdot10^{-3}$	$0.03(1)\cdot10^{-3}$	$0.47(1)\cdot10^{-5}$	$0.03(1)\cdot10^{-3}$	$2.21(1)\cdot10^{-3}$	$2.73(1)\cdot10^{-3}$	$3.67(4)\cdot10^{-2}$
Parameter							
$\Delta H^{\#}$ / kJ mol^{-1}	80.0(6)	89.2(2.3)	65.2(3.8)	–	–	–	50.3(1.5)
$\Delta S^{\#}$ / J K^{-1} mol^{-1}	–70.2(1.8)	–56.4(6.8)	–146(11)	–	–	–	–117(4)
$E^{\#}$ / kJ mol^{-1}	84.0(6)	86.2(4.9)	67.2(6.2)	–	–	–	57.0(1.3)
"${}^{d}k_{obs}$" (25 °C), s^{-1}	$1.18\cdot10^{-5}$	$2.43\cdot10^{-6}$	$5.85\cdot10^{-7}$	–	–	–	$6.30\cdot10^{-3}$
${}^{d}\tau_{1/2}$ (25 °C), hours	~16	~79	~330	–	–	–	~0.03 (~1.8 min)

[a]The 1 mM complex concentration was used for the experiments.

Table S15. Observed decomplexation rate constants ${}^{d}k_{obs}$ of the Ce(III)–, Eu(III)–, Er(III)– and Yb(III)–H_4pyta complexes of at different acidities (I = 5.0 M (H,Na)ClO_4 or 5.0 M (H,Na)Cl in blue, 90 °C). Data for the Er(III)– and Yb(III)–H_4pyta complexes (grey-highlighted) are distorted by the mutual *22*/*21* isomerisation (its extent varies with the acid concentrations).

c_{HClO4}	^{22}[Ce(**L**)]$^-$	^{22}[Ce(**L**)]$^-$ (blue)	^{22}[Eu(**L**)]$^-$	^{22}[Er(**L**)]$^-$	^{22}[Er(**L**)]$^-$ [a]	^{21}[Er(**L**)]$^-$ [a]	^{21}[Er(**L**)]$^-$	^{21}[Yb(**L**)]$^-$	^{21}[Yb(**L**)]$^-$ (blue)
5.0 M	$1.23(1)\cdot10^{-3}$	$3.28(4)\cdot10^{-3}$	$7.34(3)\cdot10^{-5}$	$1.13(1)\cdot10^{-3}$	$3.48(5)\cdot10^{-5}$	$2.21(1)\cdot10^{-3}$	$3.16(7)\cdot10^{-2}$	$2.18(1)\cdot10^{-2}$	$3.18(4)\cdot10^{-3}$
4.0 M	$1.11(2)\cdot10^{-3}$	$3.15(6)\cdot10^{-3}$	$7.84(16)\cdot10^{-5}$	$1.22(2)\cdot10^{-3}$	$3.14(4)\cdot10^{-5}$	$2.10(1)\cdot10^{-3}$	$1.56(6)\cdot10^{-2}$	$2.07(1)\cdot10^{-2}$	$1.14(1)\cdot10^{-2}$
3.0 M	$0.89(2)\cdot10^{-3}$	$3.06(4)\cdot10^{-3}$	$7.23(9)\cdot10^{-5}$	$2.10(9)\cdot10^{-3}$	$2.53(6)\cdot10^{-5}$	$1.96(1)\cdot10^{-3}$	$1.68(3)\cdot10^{-2}$	$2.84(1)\cdot10^{-2}$	$1.58(1)\cdot10^{-2}$
2.0 M	$0.79(2)\cdot10^{-3}$	$2.90(3)\cdot10^{-3}$	$7.24(4)\cdot10^{-5}$	$2.80(7)\cdot10^{-3}$	$2.23(4)\cdot10^{-5}$	$1.83(1)\cdot10^{-3}$	$2.17(3)\cdot10^{-2}$	$2.90(1)\cdot10^{-2}$	$2.45(1)\cdot10^{-2}$
1.0 M	$0.68(1)\cdot10^{-3}$	$2.79(4)\cdot10^{-3}$	$6.67(3)\cdot10^{-5}$	$3.50(9)\cdot10^{-3}$	$1.91(4)\cdot10^{-5}$	$1.73(1)\cdot10^{-3}$	$3.96(1)\cdot10^{-2}$	$4.95(6)\cdot10^{-2}$	$3.87(4)\cdot10^{-2}$
0.5 M	$0.63(1)\cdot10^{-3}$	$2.69(4)\cdot10^{-3}$	–	$6.38(6)\cdot10^{-3}$	$1.78(2)\cdot10^{-5}$	$1.65(1)\cdot10^{-3}$	$3.32(1)\cdot10^{-2}$	$4.72(3)\cdot10^{-2}$	$5.69(6)\cdot10^{-2}$
Parameter									
${}^{d}k_0$ / s^{-1}	$5.4(2)\cdot10^{-4}$	$2.7(1)\ 10^{-3}$	$6.5(2)\cdot10^{-5}$		$1.52(7)\cdot10^{-5}$	$1.59(1)\cdot10^{-3}$			
${}^{d}k_H$ / dm^3 mol^{-1} s^{-1}	$1.3(1)\cdot10^{-4}$	$1.3(1)\ 10^{-4}$	$2.7(7)\cdot10^{-6}$		$3.86(24)\cdot10^{-6}$	$1.24(1)\cdot10^{-4}$			

[a]Measured at 50 °C; complex isomerisation is suppressed at this temperature.

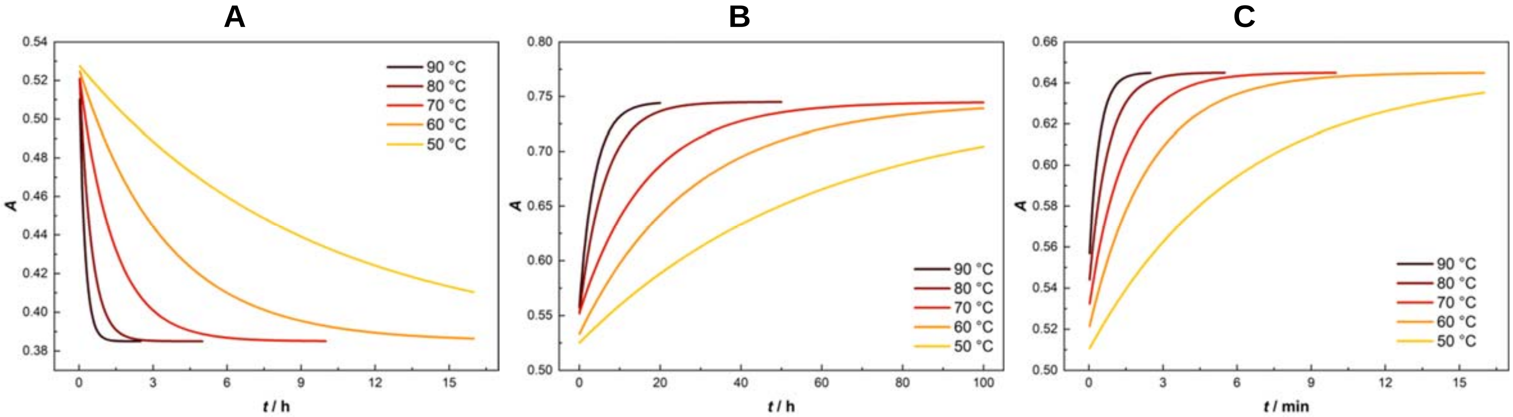

Figure S25. Fits of decomplexation data for the ^{22}[Ce(pyta)]$^{-}$ (**A**) ^{220}[Eu(pyta)]$^{-}$ (**B**) and ^{211}[Yb(pyta)]$^{-}$ (data are distorted by the isomerisation, **C**) complexes at different temperatures (5.0 M $HClO_4$) according to Equation S1.

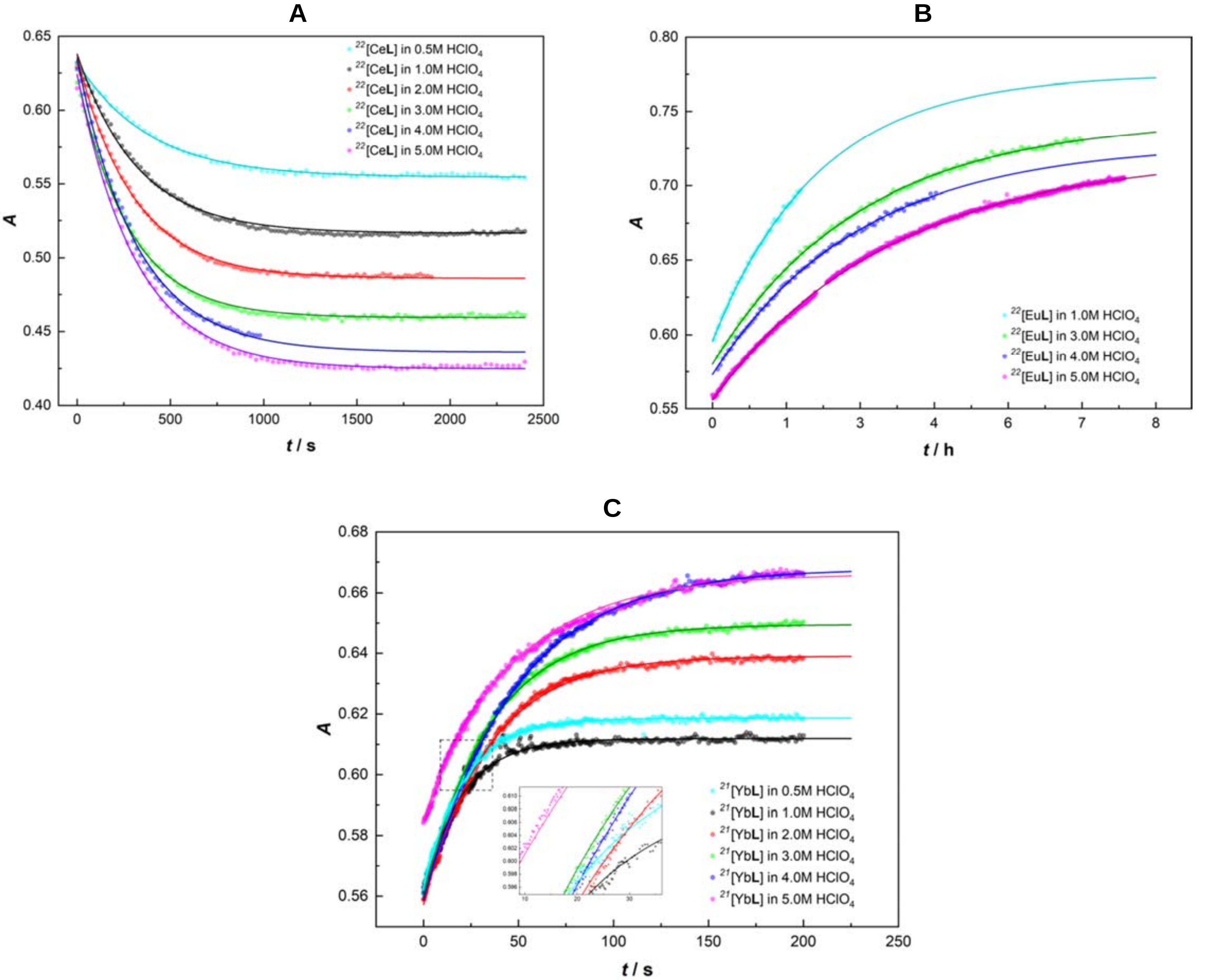

Figure S26. Decomplexation data for the ^{22}[Ce(pyta)]$^{-}$ (LMCT band; **A**); ^{22}[Eu(pyta)]$^{-}$ (pyridine band; **B**) and ^{21}[Yb(pyta)]$^{-}$ (pyridine band; data are distorted by isomerisation; **C**) complexes at different acidities (I = 5.0 M (H,Na)ClO_4, 90 °C); fits according to Equation S1.

Table S16. Decomplexation data for $[Ce(H_2O)(dota)]^-$ (1.0–5.0 M $HClO_4$, I = 5.0 (H,Na)ClO_4, 50 °C) and $[La(macropa)]^+$ (0.1–1.0 M $HClO_4$, I = 5.0 (H,Na)ClO_4, 25 °C) complexes. Fits according to Equation S7.

c_{HClO4}	$[Ce(dota)]^-$, ${}^{d}k_{obs}$	c_{HClO4}	$[La(macropa)]^+$, ${}^{d}k_{obs}$
5.0 M	$36.7(4)\cdot10^{-3}$	1.0 M	$62.6(7)\cdot10^{-3}$
4.0 M	$32.1(2)\cdot10^{-3}$	0.75 M	$50.2(1)\cdot10^{-3}$
3.0 M	$27.0(1)\cdot10^{-3}$	0.50 M	$38.3(3)\cdot10^{-3}$
2.0 M	$19.9(1)\cdot10^{-3}$	0.25 M	$23.2(1)\cdot10^{-3}$
1.0 M	$14.7(1)\cdot10^{-3}$	0.10 M	$16.5(1)\cdot10^{-3}$
${}^{d}k_0$ / s^{-1}	$9.16(80)\cdot10^{-3}$	${}^{d}k_0$ / s^{-1}	$11.2(10)\cdot10^{-3}$
${}^{d}k_H$ / dm^3 mol^{-1} s^{-1}	$5.63(24)\cdot10^{-3}$	${}^{d}k_H$ / dm^3 mol^{-1} s^{-1}	$51.9(16)\cdot10^{-3}$
${}^{d}\tau_{½}$ (1 M / 5 M acid)	47 s / 19 s	${}^{d}\tau_{½}$ (1 M acid)	11 s

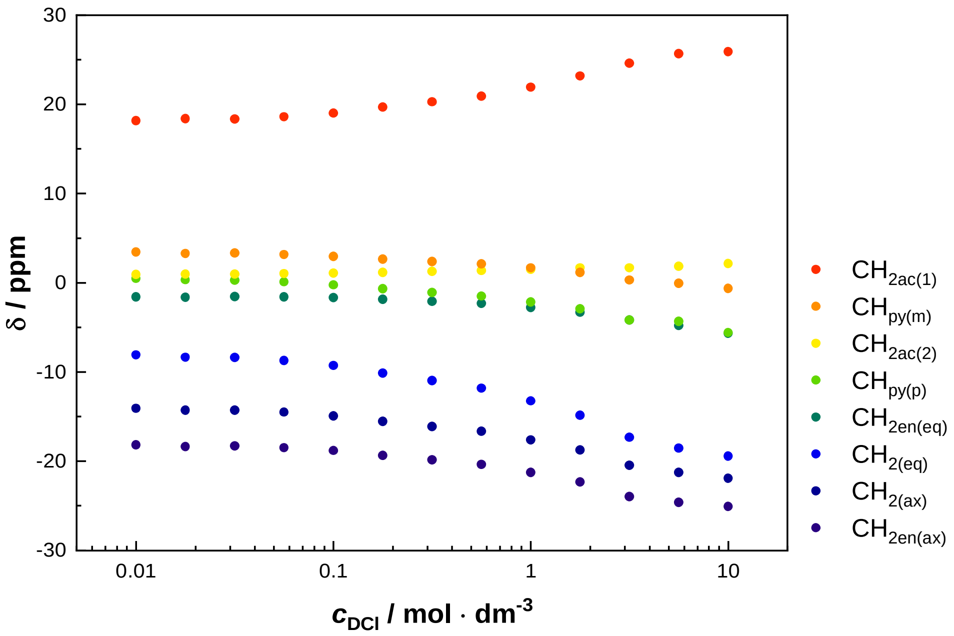

Figure S27. The ^{1}H NMR titration of the [22]$[Eu(pyta)]^-$ complex in acidic solutions (D_2O, c_{DCl} from ~0.01 M to ~10 M, room temperature, no control of ionic strength). Fit of the curves with OPIUM[11] led to protonation constants logK_a with very approximate values –0.4 and –0.3. The proton assignment was done according to the data in ref. 3.

References

1. E. P. Papadopoulos, A. Jarrar, C. H. Issidorides, *J. Org. Chem.* **1966**, *31*, 615–616.
2. (*a*) K. Abid, D. E. Fenton, *Inorg. Chim. Acta*, **1984**, *95*, 119–125. (*b*) L. Rothermel, L. Miao, A. L. Hill, S. C. Jackets, *Inorg. Chem.* **1992**, *31*, 4854–4859.
3. L. Valencia, J. Martinez, A. Macías, R. Bastida, R. A. Carvalho, C. F. G. C. Geraldes, *Inorg. Chem.* **2002**, *41*, 5300–5312.
4. L. Krause, R. Herbst-Irmer, G. M. Sheldrick, D. Stalke, *J. Appl. Cryst.* **2015**, *48*, 3–10.
5. (*a*) G. M. Sheldrick, *SHELXT2018/2. Program for Crystal Structure Solution from Diffraction Data*, University of Göttingen, Göttingen, 2018; (*b*) G. M. Sheldrick, *Acta Crystallogr. Sect. A* **2008**, *A64*, 112–122.
6. (*a*) C. B. Hübschle, G. M. Sheldrick and B. Dittrich, *ShelXle: a Qt graphical user interface for SHELXL*, University of Göttingen, Göttingen, **2014**. (*b*) C. B. Hübschle, G. M. Sheldrick, B. Dittrich, *J. Appl. Cryst.* **2011**, *44*, 1281–1284. (*c*) G. M. Sheldrick, *Acta Crystallogr. Sect. C* **2015**, *C71*, 3–8. (*d*) G. M. Sheldrick, *SHELXL-2017/1. Program for Crystal Structure Refinement from Diffraction Data*, University of Göttingen, Göttingen, **2017**.
7. (*a*) A. L. Spek, *PLATON. A Multipurpose Crystallographic Tool*, Utrecht University, Utrecht, **2005**; (*b*). A. L. Spek, *Acta Crystallogr. Sect. C* **2015**, *C71*, 9–18.
8. V. Kubíček, J. Havlíčková, J. Kotek, G. Tircsó, P. Hermann, É. Tóth, I. Lukeš, *Inorg. Chem.* **2010**, *49*, 10960–10969.
9. M. Försterová, I. Svobodová, P. Lubal, P. Táborský, J. Kotek, P. Hermann, I. Lukeš, *Dalton Trans.* **2007**, 535–549.
10. J. Notni, P. Hermann, J. Havlíčková, J. Kotek, V. Kubíček, J. Plutnar, N. Loktionova, P. J. Riss, F. Rösch, I. Lukeš, *Chem. Eur. J.* **2010**, *16*, 7174–7185.
11. (*a*) M. Kývala, I. Lukeš, *International Conference Chemometrics '95*, Pardubice, Czech Republic, **1995**, p. 63; (*b*) M. Kývala, P. Lubal, I. Lukeš, *IX. Spanish-Italian and Mediterranean Congress on Thermodynamics of Metal Complexes (SIMEC 98)*, Girona, Spain, **1998**. The full version of the OPIUM program is available (free of charge) on http://www.natur.cuni.cz/_kyvala/opium.html.
12. (*a*) A. E. Martell, R. M. Smith, *Critical Stability Constants*, Vols. 1–6, Plenum Press, New York, **1974–1989**; (*b*) *NIST Standard Reference Database 46 (Critically Selected Stability Constants of Metal Complexes)*, Version 7.0, **2003**; (*c*) C. F. Baes, Jr., R. E. Mesmer, *The Hydrolysis of Cations*, Wiley, New York, **1976**.
13. L. Miao, D. Bell, G. L. Rothremel, Jr., L. H. Bryant, Jr., P. M. Fitzsimmons, S. C. Jackels, *Supramol. Chem.* **1996**, *6*, 365–373.
14. M. Pniok, V. Kubíček, J. Havlíčková, J. Kotek, A. Sabatie-Gogová, J. Plutnar, S. Huclier-Markai, P. Hermann, *Chem. Eur. J.* **2014**, *20*, 7944–7955.
15. A. Roca-Sabio, M. Mato-Iglesias, D. Esteban-Gómez, É. Tóth, A. Blas, C. Platas-Iglesias, T. Rodríguez-Blas, *J. Am. Chem. Soc.* 2009, *131*, 3331–3341.
16. A. Hu, E. Aluicio-Sarduy, V. Brown, S. N. MacMillan, K. V. Becker, T. E. Barnhart, V. Radchenko, C. F. Ramogida, J. W. Engle, J. J. Wilson, *J. Am. Chem. Soc.* 2021, *143*, 10429−10440.
17. A. Hu, M. E. Simms, V. Kertesz, J. J. Wilson, N. A. Thiele, *Inorg. Chem.* **2022**, *61*, 12847–12855.
18. E. Brücher, *Top. Curr. Chem.* **2002**, *221*, 103–122.
